# Supplementary material for: Improved integration of single-cell transcriptome and surface protein expression by LinQ-View
Source: Cell Rep Methods. 2021 Jul 23;1(4):100056. doi: 10.1016/j.crmeth.2021.100056 (PMC9017149; doi:10.1016/j.crmeth.2021.100056)
Supplement: Document S1. Figures S1–S7 and Tables S1–S7 [file mmc1.pdf]

Cell Reports Methods, Volume *1*

Supplemental information

Improved integration of single-cell transcriptome and surface protein expression by LinQ-View

Lei Li, Haley L. Dugan, Christopher T. Stamper, Linda Yu-Ling Lan, Nicholas W. Asby, Matthew Knight, Olivia Stovicek, Nai-Ying Zheng, Maria Lucia Madariaga, Kumaran Shanmugarajah, Maud O. Jansen, Siriruk Changrob, Henry A. Utset, Carole Henry, Christopher Nelson, Robert P. Jedrzejczak, Daved H. Fremont, Andrzej Joachimiak, Florian Krammer, Jun Huang, Aly A. Khan, and Patrick C. Wilson

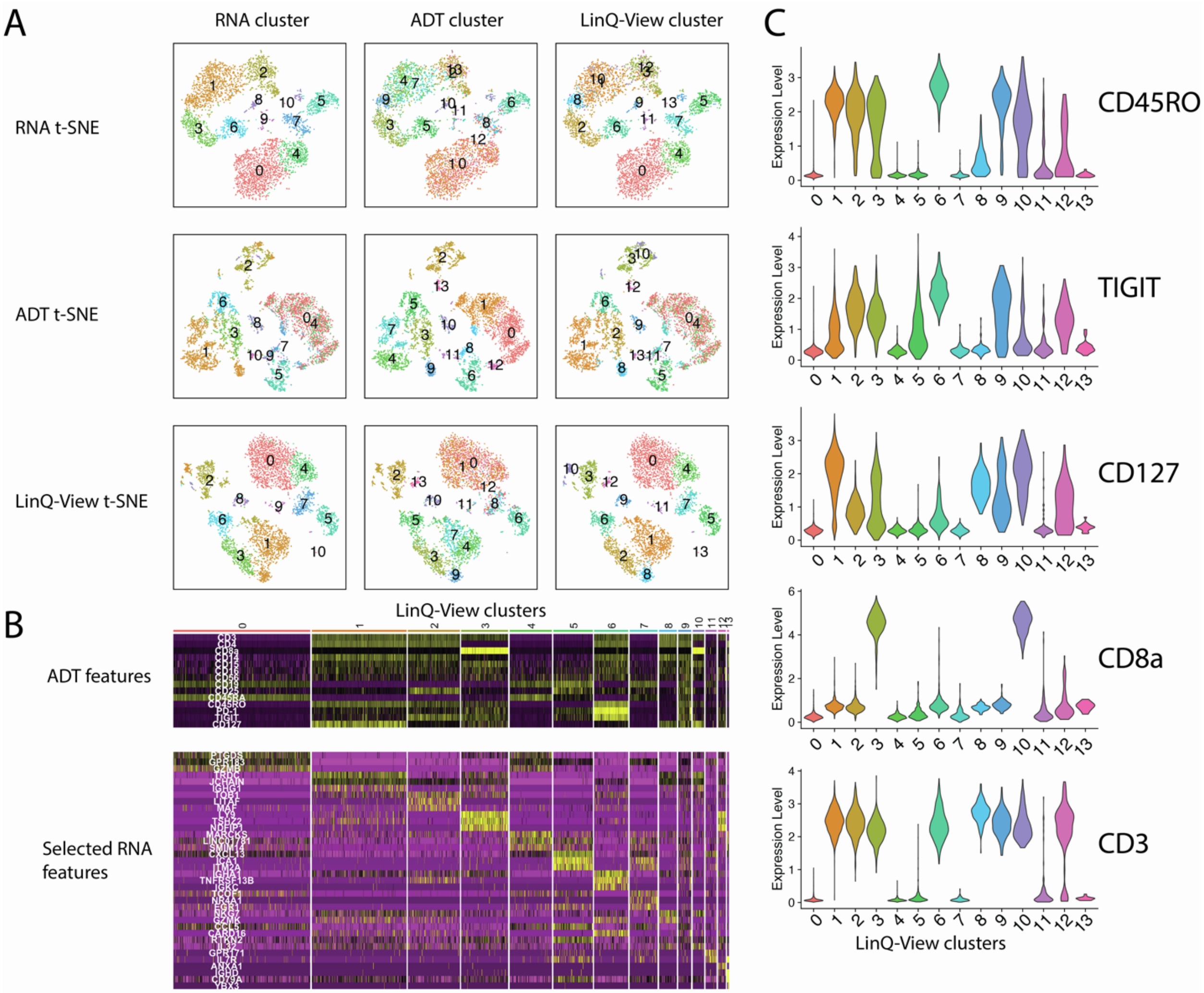

Figure S1. **LinQ-View analysis achieves better cell clustering then conventional unimodal methods by integrating both transcriptome and surface protein signals (Dataset 4), related to Figure 4**. (**A**) All combinations with three different clustering sources and three different cell embeddings are shown in a 3X3 grid. Those clusters were identified using Seurat 3 under a resolution = 0.6 for all three clustering sources (RNA, ADT and LinQ-View). Cells in RNA cluster 1 were divided into two clusters (1, 8, 10) by the LinQ-View method, and cells in RNA cluster 2 were divided into three clusters (3, 12) by the LinQ-View method. LinQ-View clusters 8 can be identified using RNA by increasing resolution to 1.2, whereas LinQ-View cluster 10 and 12 cannot be distinguished by RNA. (**B**) Gene expression profiles on both surface protein level and transcriptome level for cell clusters based on RNA+ADT using LinQ-View. (**C**) Protein expression levels for cell groups identified by LinQ-View method. Cluster 10 has distinct CD8a expression from cluster 1 and 8, cluster 3 and 12 have distinct expression of CD8a protein.

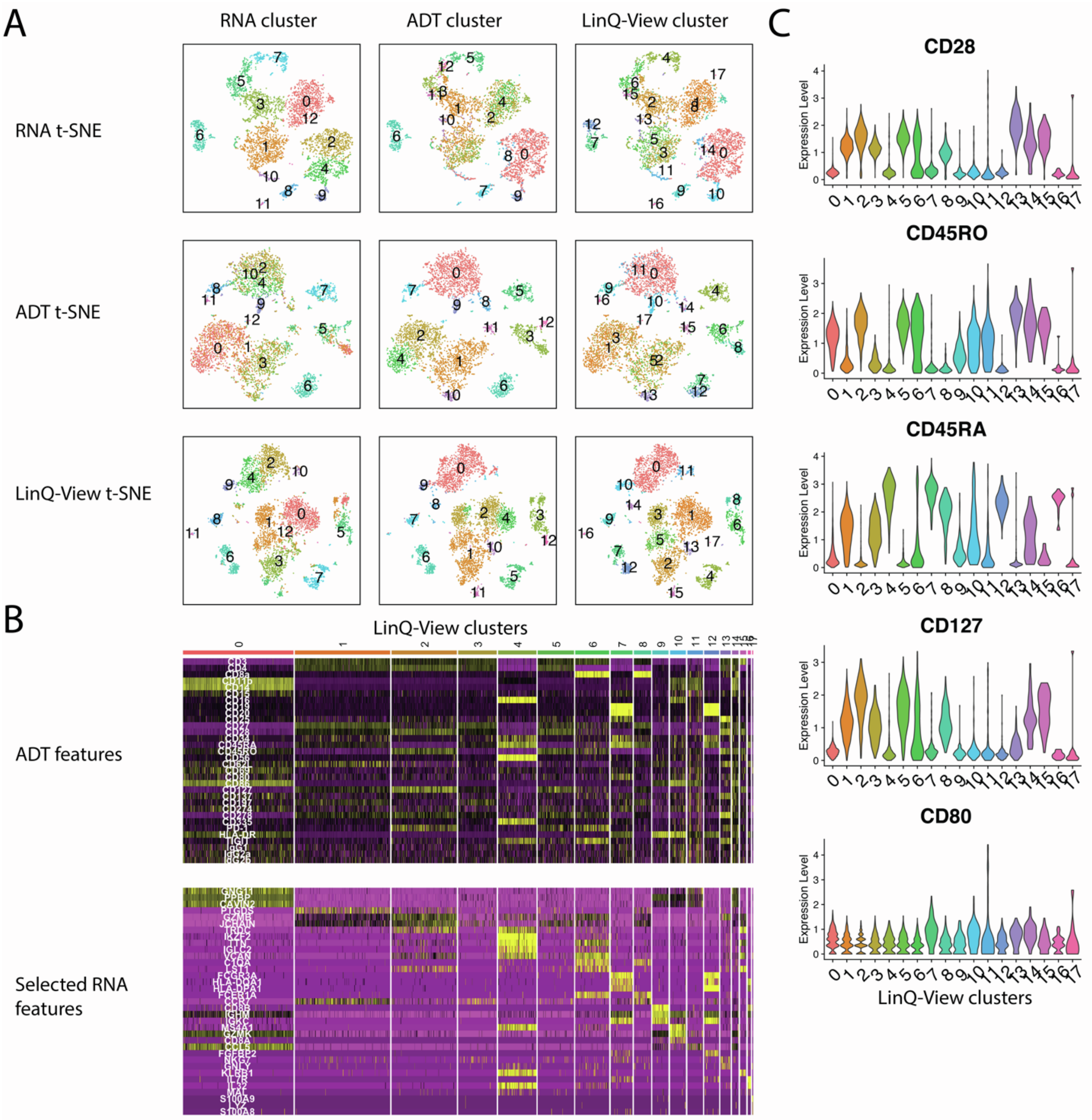

Figure S2. LinQ-View achieves better cell clustering then conventional unimodal methods by integrating both transcriptome and surface protein signals (Dataset 5) , related to Figure 4. (**A**) All combinations with three different clustering sources and three different cell embeddings are shown in a 3X3 grid. Those clusters were identified using Seurat 3 under a resolution = 0.6 for all three clustering sources (RNA, ADT and LinQ-View). As indicated by red and blue circles, cells in RNA cluster 1 were divided into two clusters (3, 5) by the LinQ-View method. (**B**) Gene expression profiles on both surface protein level and transcriptome level for cell clusters based on RNA+ADT using LinQ-View. (**C**) Protein expression levels for cell groups identified by LinQ-View method. Cluster 3 and 5 have distinct expression of CD45RO and CD45RA protein.

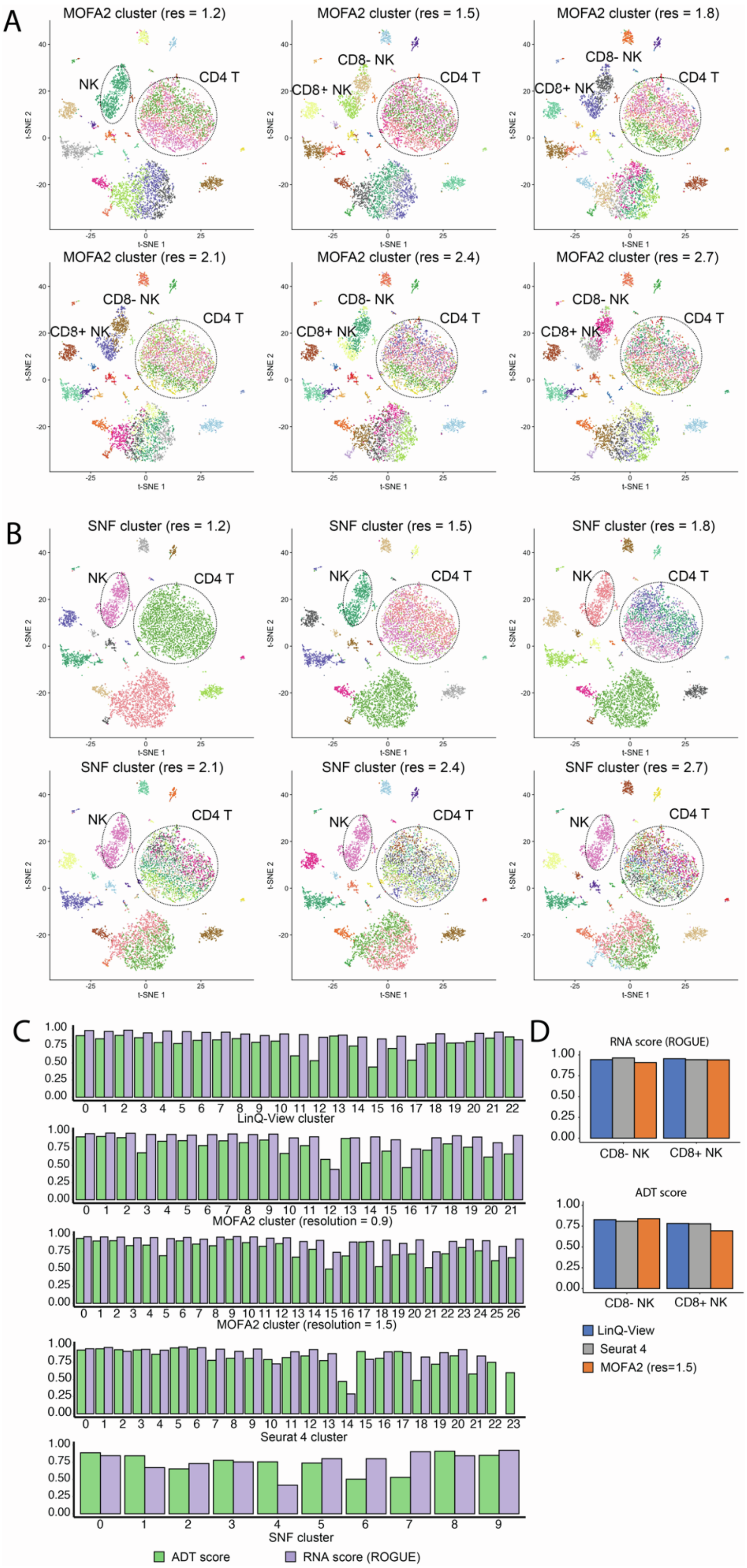
A
MOFA2 cluster (res = 1.2)
MOFA2 cluster (res = 1.5)
MOFA2 cluster (res = 1.8)
MOFA2 cluster (res = 2.1)
MOFA2 cluster (res = 2.4)
MOFA2 cluster (res = 2.7)
NK
CD4 T
CD8- NK
CD8+ NK
t-SNE 1
t-SNE 2
B
SNF cluster (res = 1.2)
SNF cluster (res = 1.5)
SNF cluster (res = 1.8)
SNF cluster (res = 2.1)
SNF cluster (res = 2.4)
SNF cluster (res = 2.7)
C
LinQ-View cluster
MOFA2 cluster (resolution = 0.9)
MOFA2 cluster (resolution = 1.5)
Seurat 4 cluster
SNF cluster
ADT score
RNA score (ROGUE)
D
RNA score (ROGUE)
ADT score
CD8- NK
CD8+ NK
LinQ-View
Seurat 4
MOFA2 (res=1.5)

Figure S3. **Comparing four existing multimodal methods, related to Figure 6.** (**A**) Performance of "MOFA2" method on distinguishing $CD4^+$ T-cell subgroups and NK cell subgroups under a series of increased resolution. (**B**) Performance of "SNF" method on distinguishing $CD4^+$ T-cell subgroups and NK cell subgroups under a series of increased resolution. (**C**) Purity scores among four multimodal clustering methods. (**D**) Comparison of purity scores in distinguishing NK cells among three multimodal methods.

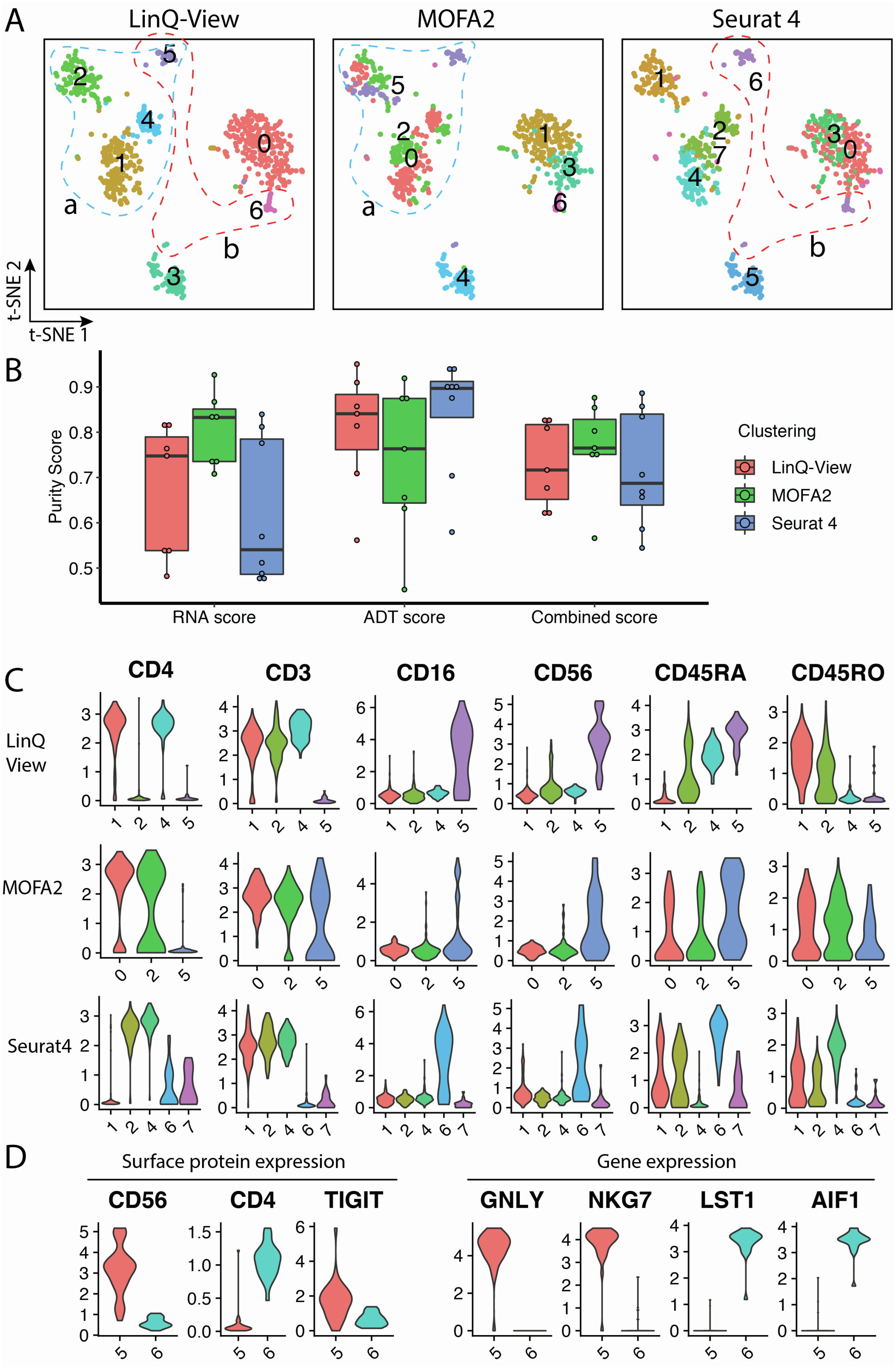
A
LinQ-View
MOFA2
Seurat 4
t-SNE 2
t-SNE 1
B
Purity Score
RNA score
ADT score
Combined score
Clustering
LinQ-View
MOFA2
Seurat 4
C
CD4
CD3
CD16
CD56
CD45RA
CD45RO
LinQ View
MOFA2
Seurat4
D
Surface protein expression
Gene expression
CD56
CD4
TIGIT
GNLY
NKG7
LST1
AIF1

Figure S4. Comparison of cell heterogeneity identification among LinQ-View, MOFA2, and Seurat 4 on dataset 2, related to Figure 6. We compared the LinQ-View method with two existing multimodal methods: MOFA2 and Seurat 4. All methods were applied on a public CITE-seq benchmark dataset (Dataset 2). Resolution for Louvain clustering was set to 0.6. (A) Clustering results of three multimodal methods. All clustering was visualized on the same (LinQ-View) t-SNE embedding. Two groups of cells that will be further discussed were indicated by dashed lines and labels. (B) Purity scores of cell clusters generated by three methods. (C) Comparison of clusters of the group “a” cells generated by LinQ-View, MOFA2, and Seurat4 on the expression of six ADT markers. (D) Comparison of clusters of group “b” cells generated by LinQ-View, MOFA2, and Seurat4 on the expression of three ADT markers and four gene markers.

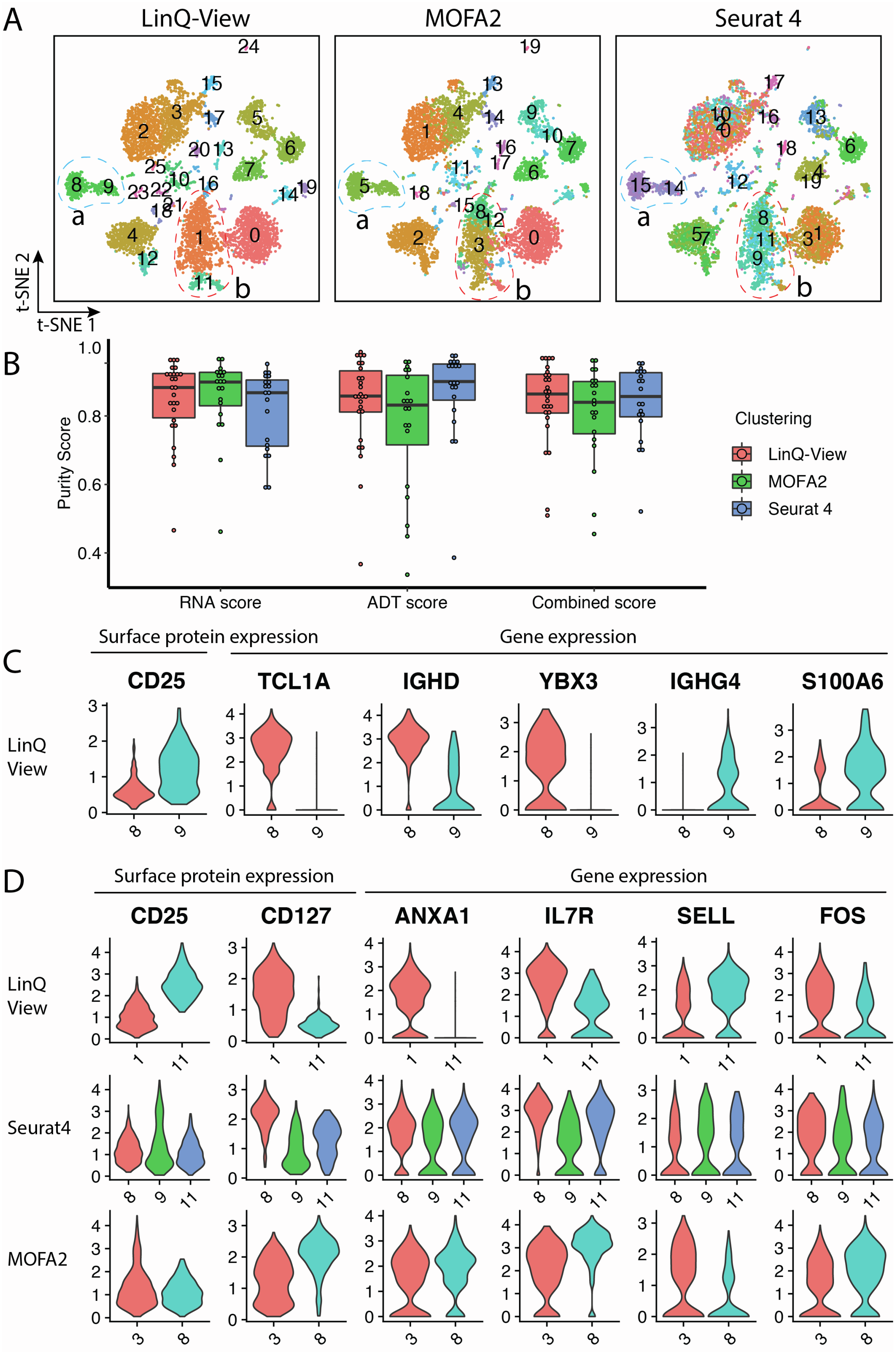
A
LinQ-View
MOFA2
Seurat 4
t-SNE 2
t-SNE 1
B
Purity Score
RNA score
ADT score
Combined score
Clustering
LinQ-View
MOFA2
Seurat 4
C
Surface protein expression
Gene expression
CD25
TCL1A
IGHD
YBX3
IGHG4
S100A6
LinQ View
D
Surface protein expression
Gene expression
CD25
CD127
ANXA1
IL7R
SELL
FOS
LinQ View
Seurat4
MOFA2

Figure S5. Comparison of cell heterogeneity identification among LinQ-View, MOFA2, and Seurat 4 on dataset 3, related to Figure 6. We compared the LinQ-View method with two existing multimodal methods: MOFA2 and Seurat 4. All methods were applied on a public CITE-seq benchmark dataset (Dataset 3). Resolution for Louvain clustering was set to 0.8. (A) Clustering results of three multimodal methods. All clustering was visualized on the same (LinQ-View) t-SNE embedding. Two groups of cells that will be further discussed were indicated by dashed lines and labels. (B) Purity scores of cell clusters generated by three methods. (C) Comparison of clusters of the group “a” cells generated by LinQ-View, MOFA2, and Seurat4 on the expression of one ADT marker and five gene markers. (D) Comparison of clusters of group “b” cells generated by LinQ-View, MOFA2, and Seurat4 on the expression of two ADT markers and four gene markers.

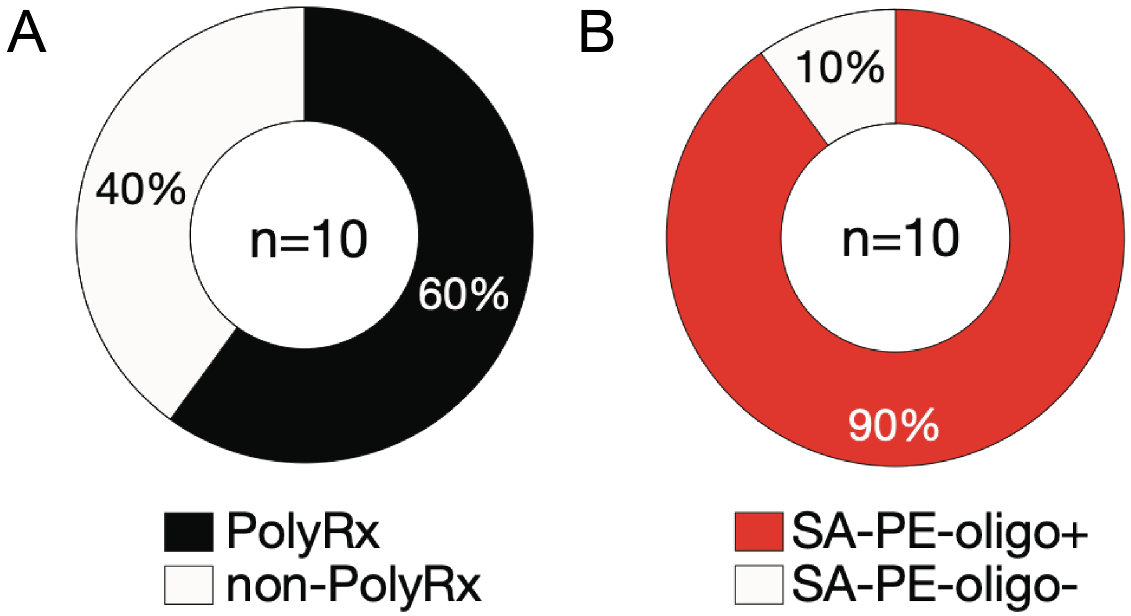

Figure S6. **Experimental characterization of non-specific nature of polyreactive B cells, related to Figure 7.** Monoclonal antibodies (mAbs) identified as multi-probe reactive were tested by ELISA against a panel of standardized antigens (dsDNA, insulin, flagellin, KLH, LPS and cardiolipin in a polyreactivity ELISA assay (A) as well as streptavidin-PE-oligo (SA-PE-oligo) used to conjugated the sorting probes (B). Numbers in the center of each pie chart indicate number of mAbs tested.

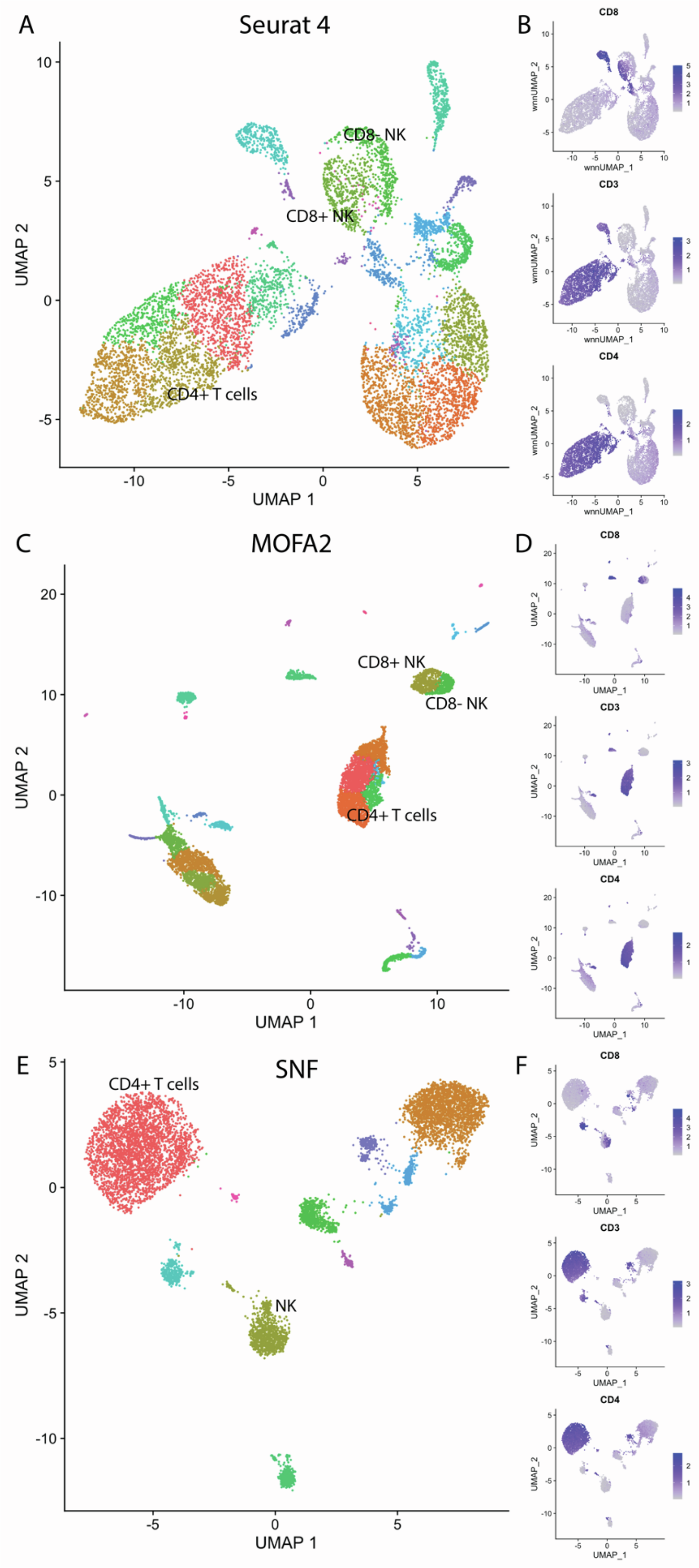
A
Seurat 4
CD8- NK
CD8+ NK
CD4+ T cells
UMAP 2
UMAP 1
B
CD8
CD3
CD4
wnnUMAP_2
wnnUMAP_1
C
MOFA2
CD8+ NK
CD8- NK
CD4+ T cells
D
E
SNF
CD4+ T cells
NK
F

Figure S7. Cell clusters generated by three existing multimodal methods, Seurat 4, MOFA2, and SNF, were visualized on their optimized UMAP embedding, related to Figure 6. (**A**) Cell subsets generated by Seurat 4 were visualized on wnnUMAP embedding. (**B**) Expression of CD8, CD3 and CD4 proteins on Seurat 4 wnnUMAP embedding. (**C**) Cell subsets generated by MOFA2 were visualized on UMAP embedding. (**D**) Expression of CD8, CD3 and CD4 proteins on MOFA2 UMAP embedding. (**E**) Cell subsets generated by SNF were visualized on UMAP embedding. (**F**) Expression of CD8, CD3 and CD4 proteins on SNF UMAP embedding.

Table S1. Assessing the quality of NK subsets generated by three different multimodal methods using RNA and ADT purity scores, related to Figure 6.

	Method	CD8+ NK	CD8- NK	Average
RNA score	LinQ-View	0.9552	0.9434	0.9493
	MOFA2(res=1.5)	0.9431	0.9646	0.9539
	Seurat4	0.9408	0.9082	0.9245
ADT score	LinQ-View	0.7822	0.8279	0.8051
	MOFA2(res=1.5)	0.6945	0.8390	0.7668
	Seurat4	0.7787	0.8089	0.7938

Table S2. Assessing the quality of CD4 T cell subsets generated by three different multimodal methods using RNA and ADT purity scores, related to Figure 6.

Method	RNA score	ADT score	Cluster ID
LinQ-View	0.971	0.8965	0
	0.9756	0.9005	2
MOFA2(res=1.5)	0.97	0.9463	0
	0.9679	0.908	1
	0.9649	0.9131	2
	0.9681	0.8847	10
Seurat4	0.9474	0.9273	0
	0.966	0.9467	1
	0.973	0.9584	5
	0.9628	0.9427	6
	0.7267	0.7903	10

Table S3. Top 20 gene markers identified among 5 cell subsets identified by Seurat4 from $CD4^+$ T cells. Gene markers were identified using "FindAllMarkers" function from the entire dataset, related to Figure 6.

**Gene**	**p_val**	**avg_log2FC**	**pct.1**	**pct.2**	**p_val_adj**	**Cluster**
IL7R	1.69E-194	1.276101	0.867	0.363	3.46E-190	0
LDHB	2.76E-175	1.094745	0.957	0.593	5.67E-171	0
IL32	9.81E-169	1.047846	0.902	0.412	2.01E-164	0
LTB	5.47E-158	1.022945	0.98	0.597	1.12E-153	0

ITM2A	2.15E-150	1.167231	0.786	0.368	4.40E-146	0
CD3E	3.52E-145	1.123032	0.78	0.358	7.21E-141	0
TRAC	8.33E-143	1.108343	0.768	0.339	1.71E-138	0
CD3D	7.51E-136	1.005698	0.855	0.43	1.54E-131	0
NOSIP	3.26E-133	1.045382	0.832	0.455	6.68E-129	0
TRBC2	1.25E-116	0.975419	0.858	0.496	2.55E-112	0
TXNIP	1.02E-103	0.857804	0.926	0.668	2.10E-99	0
SELL	6.87E-101	0.871167	0.853	0.526	1.41E-96	0
SOX4	1.22E-99	0.993059	0.68	0.336	2.51E-95	0
CD27	3.44E-88	0.926274	0.548	0.241	7.05E-84	0
LEF1	1.52E-86	0.924831	0.553	0.248	3.11E-82	0
CD3G	7.20E-86	0.886763	0.603	0.282	1.48E-81	0
PIK3IP1	1.28E-83	0.917302	0.517	0.227	2.62E-79	0
PRKCQ-AS1	1.14E-72	0.905752	0.45	0.194	2.34E-68	0
CAMK4	7.53E-67	0.862052	0.415	0.178	1.54E-62	0
MYC	6.25E-56	0.878191	0.393	0.179	1.28E-51	0
IL7R	2.72E-187	1.309592	0.884	0.368	5.58E-183	1
LDHB	5.83E-161	1.090877	0.964	0.598	1.20E-156	1
LTB	1.07E-154	1.099006	0.986	0.602	2.19E-150	1
ITM2A	1.60E-152	1.254165	0.817	0.37	3.28E-148	1
CD3D	3.28E-148	1.068008	0.898	0.431	6.72E-144	1
TRAC	9.55E-148	1.21783	0.803	0.341	1.96E-143	1
CD3E	3.18E-138	1.04337	0.82	0.359	6.52E-134	1
IL32	2.25E-137	1.024577	0.894	0.419	4.61E-133	1
TRBC2	1.49E-108	0.949362	0.879	0.498	3.06E-104	1
NOSIP	4.11E-108	0.960899	0.828	0.461	8.43E-104	1
LEF1	3.63E-92	0.973727	0.589	0.249	7.44E-88	1
CD27	3.19E-87	0.948812	0.571	0.243	6.53E-83	1
SELL	2.18E-85	0.865475	0.839	0.532	4.47E-81	1
CD3G	1.01E-84	0.909714	0.622	0.284	2.07E-80	1
OCIAD2	1.46E-77	0.917021	0.622	0.313	2.99E-73	1
CAMK4	3.68E-77	0.929688	0.455	0.176	7.55E-73	1
PIK3IP1	6.22E-75	0.869511	0.527	0.229	1.27E-70	1
SOX4	7.63E-71	0.884298	0.651	0.344	1.56E-66	1
CORO1B	7.60E-68	0.91084	0.603	0.321	1.56E-63	1
MYC	3.89E-56	0.870035	0.41	0.18	7.98E-52	1
RPL3	2.76E-104	0.848651	1	0.864	5.65E-100	5
LTB	1.44E-103	1.025716	0.993	0.614	2.96E-99	5
IL7R	1.04E-97	1.192832	0.824	0.389	2.12E-93	5

IL32	4.99E-94	0.977211	0.889	0.435	1.02E-89	5
LDHB	1.99E-93	1.000393	0.935	0.612	4.08E-89	5
TRAC	1.52E-89	1.14572	0.765	0.358	3.12E-85	5
CD3E	8.33E-89	1.117801	0.778	0.377	1.71E-84	5
TRBC2	3.12E-85	1.044435	0.88	0.511	6.39E-81	5
CD3D	7.14E-84	0.969653	0.857	0.449	1.46E-79	5
ITM2A	2.41E-77	1.009084	0.769	0.388	4.95E-73	5
SOX4	4.18E-63	1.01867	0.676	0.352	8.56E-59	5
TXNIP	7.69E-61	0.832261	0.909	0.681	1.58E-56	5
CD3G	7.62E-60	0.93437	0.613	0.295	1.56E-55	5
NOSIP	3.10E-59	0.901435	0.776	0.477	6.36E-55	5
OCIAD2	6.58E-53	0.902851	0.619	0.323	1.35E-48	5
CD27	5.55E-51	0.892827	0.541	0.256	1.14E-46	5
LEF1	5.66E-50	0.917793	0.543	0.263	1.16E-45	5
PCED1B-AS1	5.79E-45	0.846703	0.646	0.374	1.19E-40	5
PRKCQ-AS1	3.44E-39	0.86229	0.435	0.207	7.05E-35	5
RHOH	2.42E-36	0.897578	0.48	0.256	4.97E-32	5
IL7R	2.01E-93	1.184367	0.833	0.391	4.12E-89	6
LTB	1.15E-86	1.062477	0.967	0.618	2.37E-82	6
LDHB	1.04E-82	0.990395	0.935	0.614	2.13E-78	6
CD3E	1.85E-76	0.97756	0.787	0.379	3.79E-72	6
IL32	2.43E-76	0.989485	0.862	0.439	4.99E-72	6
TRAC	2.96E-76	1.101981	0.758	0.361	6.08E-72	6
CD3D	1.95E-74	0.979214	0.846	0.452	4.00E-70	6
CD3G	1.32E-65	0.984378	0.644	0.295	2.71E-61	6
TMEM66	4.00E-61	0.828388	0.919	0.723	8.20E-57	6
NOSIP	2.90E-60	0.917321	0.799	0.477	5.94E-56	6
ITM2A	8.02E-58	0.897442	0.744	0.392	1.65E-53	6
TRBC2	1.64E-57	0.860316	0.843	0.515	3.36E-53	6
LEF1	8.91E-57	0.985366	0.577	0.262	1.83E-52	6
TXNIP	4.93E-53	0.835354	0.892	0.684	1.01E-48	6
CD27	2.18E-50	0.919881	0.551	0.257	4.46E-46	6
SELL	6.24E-47	0.810074	0.799	0.547	1.28E-42	6
JUN	4.14E-42	0.946035	0.699	0.427	8.49E-38	6
TRAT1	3.11E-37	0.8267	0.335	0.132	6.38E-33	6
PIK3IP1	1.44E-35	0.868924	0.478	0.244	2.94E-31	6
CORO1B	1.87E-33	0.836868	0.561	0.335	3.84E-29	6
LTB	3.01E-65	1.009752	0.96	0.623	6.18E-61	10
CD3E	5.78E-64	0.978854	0.815	0.384	1.18E-59	10

IL7R	5.32E-63	0.991946	0.828	0.398	1.09E-58	10
LDHB	8.78E-63	0.873163	0.96	0.617	1.80E-58	10
IL32	4.29E-59	0.926231	0.876	0.445	8.79E-55	10
ITM2A	5.23E-55	0.946844	0.791	0.395	1.07E-50	10
TRAC	7.64E-49	0.824591	0.757	0.367	1.57E-44	10
TRBC2	2.45E-45	0.81613	0.873	0.519	5.02E-41	10
CD3D	3.32E-45	0.749844	0.852	0.458	6.81E-41	10
LEF1	5.96E-43	0.871769	0.59	0.266	1.22E-38	10
RP4-594I10.3	5.83E-39	0.912025	0.426	0.167	1.20E-34	10
SELL	2.98E-37	0.776717	0.82	0.549	6.11E-33	10
NOSIP	1.92E-36	0.770026	0.78	0.483	3.94E-32	10
SOX4	1.14E-34	0.830781	0.667	0.359	2.34E-30	10
PRKCQ-AS1	3.13E-32	0.819758	0.474	0.21	6.41E-28	10
CD27	3.69E-32	0.799766	0.54	0.262	7.57E-28	10
OXNAD1	2.23E-30	0.777623	0.397	0.165	4.57E-26	10
CAMK4	4.87E-30	0.767102	0.434	0.192	9.99E-26	10
PCED1B-AS1	7.36E-29	0.760012	0.661	0.379	1.51E-24	10
MAL	5.89E-26	0.748734	0.307	0.12	1.21E-21	10

Table S4. Gene markers identified among 5 cell subsets identified by Seurat4 from CD4$^+$ T cells. Gene markers were identified using "FindAllMarkers" functions from a subset of CD4$^+$ T cells that only contains the 5 clusters (Cluster 0, 1, 5, 6, and 10), related to Figure 6.

Gene	P value	avg_log2FC	pct.1	pct.2	P_val_adj	Cluster
HPGD	1.02E-06	0.3006893	0.206	0.132	2.09E-02	1
KLRB1	3.51E-03	-0.6330162	0.061	0.101	1.00E+00	5
HLA-DRA	1.39E-03	-0.5163531	0.1	0.155	1.00E+00	5
DUSP1	2.77E-04	-0.4573587	0.224	0.306	1.00E+00	5
FOS	3.83E-06	-0.4569581	0.331	0.443	7.84E-02	5
HPGD	6.74E-05	-0.3458329	0.094	0.163	1.00E+00	5
MOUSE-S100a4	2.31E-03	-0.3361525	0.248	0.32	1.00E+00	5
MOUSE-Anxa1	9.39E-05	-0.3332097	0.072	0.135	1.00E+00	5
ANXA1	6.62E-04	-0.287765	0.456	0.537	1.00E+00	5
CLIC1	9.74E-05	-0.2808579	0.15	0.231	1.00E+00	5
TMEM173	1.09E-04	-0.2604576	0.067	0.127	1.00E+00	5
MOUSE-Prdx1	9.43E-03	-0.2554909	0.069	0.106	1.00E+00	5
DEF6	1.46E-04	-0.2546802	0.12	0.197	1.00E+00	5
TCF25	1.74E-04	0.2733553	0.374	0.309	1.00E+00	5
CTD-3184A7.4	3.58E-03	0.2925256	0.194	0.152	1.00E+00	5
ZFP36L1	3.94E-05	-0.3063632	0.114	0.194	8.08E-01	6

TERF2IP	2.01E-04	-0.2876009	0.244	0.328	1.00E+00	6
YBEY	6.02E-04	-0.2689975	0.061	0.113	1.00E+00	6
ARID4A	5.90E-03	0.2536122	0.114	0.078	1.00E+00	6
GIMAP4	1.75E-03	0.2621582	0.358	0.299	1.00E+00	6
KIAA0020	1.60E-03	0.2626491	0.112	0.072	1.00E+00	6
HNRNPH3	1.94E-03	0.2679293	0.238	0.184	1.00E+00	6
WHSC1L1	8.85E-03	0.2696315	0.319	0.279	1.00E+00	6
YTHDC1	1.95E-03	0.2699329	0.189	0.137	1.00E+00	6
NELL2	1.71E-03	0.274412	0.185	0.135	1.00E+00	6
IFITM1	2.65E-04	0.2938378	0.64	0.606	1.00E+00	6
SRSF7	7.14E-04	0.2945516	0.343	0.278	1.00E+00	6
S100A10	2.10E-03	0.3119543	0.459	0.411	1.00E+00	6
PSMD5-AS1	1.13E-03	-0.359732	0.257	0.321	1.00E+00	10
MYC	7.50E-03	-0.3468814	0.339	0.384	1.00E+00	10
NFKBIZ	1.48E-03	-0.2993363	0.407	0.462	1.00E+00	10
CD3G	6.47E-04	-0.2734616	0.571	0.618	1.00E+00	10
SMC4	6.18E-03	-0.2664461	0.228	0.285	1.00E+00	10
SNRPF	4.39E-03	-0.253839	0.291	0.346	1.00E+00	10
HIF1A	3.15E-03	0.2506162	0.14	0.09	1.00E+00	10
HNRNPH1	3.70E-03	0.251075	0.312	0.238	1.00E+00	10
MOB1A	3.48E-04	0.2554952	0.209	0.136	1.00E+00	10
HERPUD1	1.27E-03	0.2600055	0.206	0.143	1.00E+00	10
GRN	9.70E-06	0.2653345	0.138	0.07	1.99E-01	10
RABGGTB	1.38E-04	0.2681924	0.127	0.071	1.00E+00	10
CYBA	1.34E-04	0.2691447	0.757	0.678	1.00E+00	10
NFKBIA	6.02E-03	0.2710587	0.513	0.439	1.00E+00	10
BTN3A1	5.63E-05	0.2739037	0.108	0.055	1.00E+00	10
RPIA	8.00E-04	0.2820089	0.267	0.192	1.00E+00	10
S100A11	3.01E-03	0.2890378	0.164	0.109	1.00E+00	10
CHCHD5	7.66E-05	0.2915036	0.209	0.131	1.00E+00	10
ANXA2	7.32E-04	0.2916279	0.108	0.062	1.00E+00	10
SRGN	2.76E-03	0.3200517	0.479	0.386	1.00E+00	10
MOUSE-Ctsl	7.15E-04	0.3236493	0.132	0.08	1.00E+00	10
CLIC1	1.75E-04	0.3239855	0.294	0.206	1.00E+00	10
MOUSE-Atp5b	7.44E-03	0.3581136	0.159	0.11	1.00E+00	10
MOUSE-Esd	6.64E-04	0.3631582	0.111	0.064	1.00E+00	10
MOUSE-Ranbp1	5.33E-03	0.3678064	0.132	0.087	1.00E+00	10
AP1S2	3.63E-03	0.3708234	0.254	0.193	1.00E+00	10
MOUSE-Tubb5	8.96E-04	0.3902903	0.193	0.128	1.00E+00	10

MOUSE-Ppp1ca	4.76E-05	0.3939693	0.14	0.077	9.76E-01	10
DUSP1	2.93E-04	0.3943928	0.36	0.282	1.00E+00	10
MOUSE-Cdk4	3.59E-03	0.3959323	0.127	0.08	1.00E+00	10
MOUSE-Bsg	1.25E-03	0.402012	0.209	0.142	1.00E+00	10
MOUSE-S100a10	5.08E-03	0.4590124	0.183	0.128	1.00E+00	10
MOUSE-Slc25a4	9.46E-04	0.4598929	0.161	0.103	1.00E+00	10
CD79B	2.23E-03	0.4696106	0.103	0.063	1.00E+00	10
MOUSE-Hsp90ab1	9.83E-04	0.4730225	0.177	0.117	1.00E+00	10
MOUSE-Cstb	1.34E-03	0.485548	0.138	0.086	1.00E+00	10
CTSS	1.83E-04	0.4864184	0.283	0.201	1.00E+00	10
MOUSE-Pkm	3.98E-04	0.4971615	0.124	0.072	1.00E+00	10
MOUSE-Eef1b2	1.11E-03	0.5054604	0.103	0.059	1.00E+00	10
HLA-DQB1	2.09E-15	0.5610536	0.106	0.026	4.29E-11	10
MOUSE-S100a11	1.50E-04	0.5639075	0.103	0.054	1.00E+00	10
MOUSE-Tmsb4x	6.76E-03	0.5825296	0.45	0.367	1.00E+00	10
MOUSE-Rps19	5.29E-05	0.5836074	0.122	0.064	1.00E+00	10
CD79A	3.72E-09	0.597609	0.101	0.036	7.64E-05	10
FCER1G	4.84E-04	0.5978362	0.183	0.119	1.00E+00	10
CST3	3.98E-03	0.6068051	0.201	0.143	1.00E+00	10
MOUSE-Tmsb10	8.72E-03	0.6252044	0.111	0.072	1.00E+00	10
MOUSE-Lgals1	4.26E-03	0.6288699	0.788	0.708	1.00E+00	10
MOUSE-Rplp2	1.32E-04	0.6352816	0.101	0.052	1.00E+00	10
MOUSE-S100a4	8.35E-03	0.6422301	0.368	0.299	1.00E+00	10
MOUSE-Anxa1	2.00E-06	0.6595185	0.201	0.113	4.10E-02	10
MOUSE-Cd63	1.14E-04	0.6966574	0.183	0.112	1.00E+00	10
MOUSE-Rpl26	4.84E-04	0.7055673	0.119	0.068	1.00E+00	10
HLA-DPA1	1.86E-07	0.7218892	0.108	0.046	3.81E-03	10
LGALS1	2.57E-03	0.7264715	0.172	0.12	1.00E+00	10
MOUSE-Rpl23	4.07E-03	0.7302428	0.148	0.098	1.00E+00	10
MOUSE-Rplp1	5.45E-03	0.7746256	0.138	0.091	1.00E+00	10
TYROBP	1.14E-05	0.7779309	0.23	0.141	2.33E-01	10
MOUSE-Rps15	2.97E-05	0.8176073	0.172	0.1	6.09E-01	10
MOUSE-Gm10260	5.06E-05	0.8242067	0.108	0.054	1.00E+00	10
HLA-DPB1	4.80E-15	0.8777517	0.108	0.028	9.84E-11	10
MOUSE-Rpl13a	2.25E-04	0.8910114	0.286	0.196	1.00E+00	10
HLA-DRB1	1.74E-11	0.935431	0.151	0.059	3.56E-07	10
PPBP	6.98E-07	0.9355044	0.108	0.047	1.43E-02	10
MOUSE-Rps11	3.99E-05	0.9652659	0.169	0.099	8.18E-01	10
MOUSE-Pfn1	7.98E-05	1.0513148	0.201	0.126	1.00E+00	10

CD74	1.60E-04	1.1090925	0.505	0.427	1.00E+00	10
LYZ	8.16E-03	1.2885235	0.325	0.266	1.00E+00	10
HLA-DRA	7.86E-09	1.3855268	0.238	0.132	1.61E-04	10

Table S5. Runtime (seconds) of all processing steps of LinQ-View on multiple single cell datasets, related to Figure 6.

**Dataset**	**Dataset name**	**Dataset1**	**Dataset2**	**Dataset3**	**Dataset4**	**Dataset5**	**Dataset6***
	**Number of cells**	8067	713	7865	8412	5247	19481
**Processing steps**	**Create object**	1.77	0.10	0.57	0.46	0.28	2.45
	**Normalization**	0.29	0.11	0.36	0.31	0.37	9.23
	**Identify HVGs**	1.80	1.06	2.30	2.30	1.97	
	**Data scaling**	0.47	0.26	0.58	0.44	0.38	17.70
	**PCA**	11.22	0.99	13.27	8.61	6.75	39.50
**Joint analysis**	**Joint distance calculation**	26.70	0.54	66.73	72.61	43.12	140.91
	**t-SNE**	31.57	1.32	34.22	38.63	18.33	145.90
	**Clustering**	6.04	0.17	6.47	7.29	2.60	42.56

*This is an integrated dataset from 10 individual datasets. Batch effects among all 10 datasets were removed by Seurat 3, runtime of this step is: 10.06 mins.

Table S6. Runtime (minutes) of kernel step of LinQ-View on massive single cell datasets with different cell numbers (number of ADT feature = 20), related to Figure 6.

**Cell Number**	48170	39264	30076	20799	11424
**Joint distance calculation**	39.39	22.54	12.41	5.81	1.69

Table S7. Parameters used in generating simulated dataset 1. We use absolute values in case of negative values, related to STAR Methods.

	Cluster 1		Cluster 2		Cluster 3		Cluster 4		Cluster 5		Cluster 6	
	Mean	SD	Mean	SD	Mean	SD	Mean	SD	Mean	SD	Mean	SD
Feaure1	0.5	0.5	0	0.5	0.5	0.5	0	0.5	0.5	0.5	0	0.5
Feaure2	3	0.5	3	0.5	3	0.5	3	0.5	3	0.5	3	0.5
Feaure3	1	0.5	0	0.5	2	1.5	0.5	0.5	2	0.5	2	0.5
Feaure4	0.5	0.5	2	1	2	0.5	0	0.5	1.5	0.5	3	0.5
Feaure5	2.5	0.5	0	0.5	3	0.5	3	1	0	0.5	3	1
Feaure6	3	0.5	0	0.5	0.5	0.5	3	1	2.5	1	0.5	0.5
Feaure7	2.5	0.5	2	0.5	0	0.5	3	0.5	2.5	1	2.5	1
Feaure8	0	0.5	2	0.5	3	0.5	3	0.5	2.5	1	3	1
Feaure9	0	0.5	2	0.5	0.5	0.5	3	0.5	0	0.5	3	1
