# Supplementary material for: Improved integration of single-cell transcriptome and surface protein expression by LinQ-View
Source: Cell Rep Methods. 2021 Jul 23;1(4):100056. doi: 10.1016/j.crmeth.2021.100056 (PMC9017149; doi:10.1016/j.crmeth.2021.100056)
Supplement: Document S2. Article plus supplemental information [file mmc2.pdf]

Cell Reports
Methods

Article

Improved integration of single-cell transcriptome and surface protein expression by LinQ-View

Graphical abstract

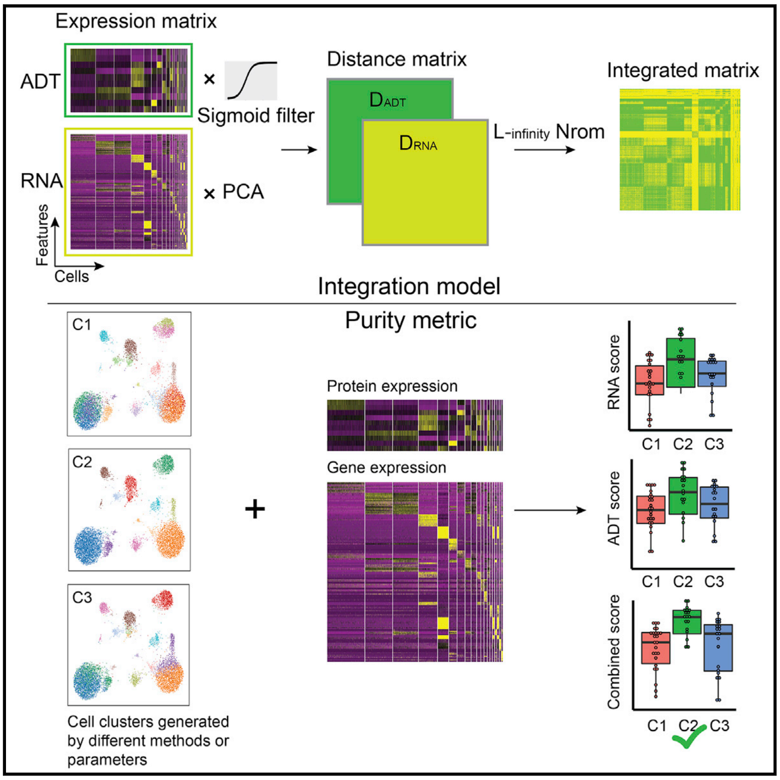

Highlights

- LinQ-View integrates mRNA and protein expression data to reveal cell heterogeneity
- LinQ-View prevents single dominant ADT features from affecting clustering
- LinQ-View presents a quantitative purity metric for CITE-seq data
- LinQ-View is specialized in handling CITE-seq data with fewer ADT features

Authors

Lei Li, Haley L. Dugan, Christopher T. Stamper, ..., Jun Huang, Aly A. Khan, Patrick C. Wilson

Correspondence

aakhan@uchicago.edu (A.A.K.), wilsonp@uchicago.edu (P.C.W.)

In brief

Li et al. present LinQ-View, a computational workflow that provides an effective solution for integrating multiple modalities of CITE-seq data and quantitative assessment of cluster purity. LinQ-View could be helpful for multimodal analysis and purity assessment of CITE-seq datasets that target specific cell populations.

Li et al., 2021, Cell Reports Methods *1*, 100056
August 23, 2021 © 2021 The Author(s).
https://doi.org/10.1016/j.crmeth.2021.100056

CellPress

Cell Reports Methods

CellPress
OPEN ACCESS

Article

Improved integration of single-cell transcriptome and surface protein expression by LinQ-View

Lei Li,[1,12] Haley L. Dugan,[2,12] Christopher T. Stamper,[2,12] Linda Yu-Ling Lan,[2] Nicholas W. Asby,[3] Matthew Knight,[1] Olivia Stovicek,[1] Nai-Ying Zheng,[1] Maria Lucia Madariaga,[4] Kumaran Shanmugarajah,[4] Maud O. Jansen,[5] Siriruk Changrob,[1] Henry A. Utset,[1] Carole Henry,[1] Christopher Nelson,[6] Robert P. Jedrzejczak,[7,8] Daved H. Fremont,[6] Andrzej Joachimiak,[7,8,9] Florian Krammer,[10] Jun Huang,[3] Aly A. Khan,[11,*] and Patrick C. Wilson[1,2,13,*]

[1]University of Chicago Department of Medicine, Section of Rheumatology, Chicago, IL 60637, USA
[2]Committee on Immunology, University of Chicago, Chicago, IL 60637, USA
[3]Pritzker School of Molecular Engineering, University of Chicago, Chicago, IL 60637, USA
[4]Department of Surgery, University of Chicago, Chicago, IL 60637, USA
[5]Section of Hospital Medicine, University of Chicago Medical Center, Chicago, IL 60637, USA
[6]Department of Pathology and Immunology, Washington University School of Medicine, St. Louis, MO 63110, USA
[7]Center for Structural Genomics of Infectious Diseases, Consortium for Advanced Science and Engineering, University of Chicago, Chicago, IL 60637, USA
[8]Structural Biology Center, X-ray Science Division, Argonne National Laboratory, Lemont, IL 60439, USA
[9]Department of Biochemistry and Molecular Biology, University of Chicago, Chicago, IL 60637, USA
[10]Department of Microbiology, Icahn School of Medicine at Mount Sinai, New York, NY 10029, USA
[11]Department of Pathology, University of Chicago, Chicago, IL 60637, USA
[12]These authors contributed equally
[13]Lead contact
*Correspondence: aakhan@uchicago.edu (A.A.K.), wilsonp@uchicago.edu (P.C.W.)
https://doi.org/10.1016/j.crmeth.2021.100056

MOTIVATION Multimodal single-cell sequencing enables multiple aspects for characterizing the dynamics of cell states and developmental processes. Properly integrating information from multiple modalities is a crucial step for interpreting cell heterogeneity. Here, we present LinQ-View, a computational workflow that provides an effective solution for integrating multiple modalities of CITE-seq data for downstream interpretation. LinQ-View balances information from multiple modalities to achieve accurate clustering results and is specialized in handling CITE-seq data with routine numbers of surface protein features.

SUMMARY

Multimodal advances in single-cell sequencing have enabled the simultaneous quantification of cell surface protein expression alongside unbiased transcriptional profiling. Here, we present LinQ-View, a toolkit designed for multimodal single-cell data visualization and analysis. LinQ-View integrates transcriptional and cell surface protein expression profiling data to reveal more accurate cell heterogeneity and proposes a quantitative metric for cluster purity assessment. Through comparison with existing multimodal methods on multiple public CITE-seq datasets, we demonstrate that LinQ-View efficiently generates accurate cell clusters, especially in CITE-seq data with routine numbers of surface protein features, by preventing variations in a single surface protein feature from affecting results. Finally, we utilized this method to integrate single-cell transcriptional and protein expression data from SARS-CoV-2-infected patients, revealing antigen-specific B cell subsets after infection. Our results suggest LinQ-View could be helpful for multimodal analysis and purity assessment of CITE-seq datasets that target specific cell populations (e.g., B cells).

INTRODUCTION

In the past decade, advances in single-cell sequencing have ushered in a new era of discovery in biology and medicine. Improved sensitivity, reliability, throughput, and sequencing depth have provided researchers immense power to study diverse biological systems at single-cell resolution (Cao et al., 2017; Hashimshony et al., 2012; Klein et al., 2015; Macosko et al., 2015; Picelli et al., 2013; Ramsköld et al., 2012; Rosenberg et al., 2018; Tang et al., 2009; Zheng et al., 2017). Recent technological advances have enabled the simultaneous measurement of multiple cell properties in combination with single-cell

This is an open access article under the CC BY license (http://creativecommons.org/licenses/by/4.0/).

RNA sequencing (scRNA-seq), allowing for an unprecedented view of cell heterogeneity. Cellular indexing of transcriptomes and epitopes by sequencing (CITE-seq) and RNA expression and protein sequencing assay are capable of measuring the expression of surface protein markers alongside the transcriptome at single-cell resolution (Peterson et al., 2017; Stoeckius et al., 2017). Because much of foundational immunology has been elaborated through the use of specific lineage-restricted cell-surface proteins to define immune cell populations, direct estimation of protein expression might enable improved methods for delineating cell populations.

To gain a better understanding of immune functions at single-cell resolution, the integration of surface protein expression and transcriptional profiling data are critical. Existing single-cell analysis software packages are typically designed for the analysis of unimodal data and provide limited integration of additional profiling modalities, such as cell-surface protein expression data. In CITE-seq, protein expression is estimated through the use of antibody-derived tags (ADTs), which promise to better characterize immune cell heterogeneity (Peterson et al., 2017; Stoeckius et al., 2017; Stuart and Satija, 2019). Recently, a few methods have been published or pre-released for integrative analysis of CITE-seq data (Argelaguet et al., 2018, 2020; Hao et al., 2020; Kim et al., 2020; Shen et al., 2009, 2013; Wang et al., 2014). Although these tools are valuable, improvements to the workflow and imbalances between transcriptome and surface protein expression are warranted. In addition, there is a lack of a quantitative metric of cluster purity for CITE-seq data. Thus, there remains a need for efficient methods capable of integrating transcriptome and surface protein expression for single-cell CITE-seq data that allow downstream analyses with deeper resolution.

To overcome these challenges, we developed a single-cell analysis toolkit called “LinQ-View” for integrative analysis of CITE-seq data. LinQ-View integrates information from transcriptome and cell-surface protein expression levels by fusing pairwise distance matrices with an L_∞ norm. On the basis of the integrative kernel, we developed a quantitative metric (purity score) to assess the purity of single-cell populations in both the transcriptome modality and the surface protein modality of CITE-seq data. Because the resulting matrix is also a distance matrix, subsequent downstream analyses can seamlessly utilize the protein- and transcriptome-linked views of single cells. By applying our integrated method to several public benchmark datasets, we demonstrated that LinQ-View is not only effective and efficient, but also superior on CITE-seq datasets with routine numbers of surface protein features, applicable to many research groups who focus on distinct areas of biology. We have made LinQ-View available on a public data repository for users worldwide at https://wilsonimmunologylab.github.io/LinQView.

RESULTS

LinQ-View combines single-cell mRNA and protein expression profiling data and enables multimodal downstream analysis

One possible representation of single-cell profiling data are based on pairwise comparisons of cells. Specifically, mRNA or protein expression profiles might be chosen as feature representations, and pairwise distances (or dissimilarities) can be calculated between all cells. The primary idea behind LinQ-View for CITE-seq is to calculate the dissimilarity representations for each single-cell profiling modality (i.e., mRNA and surface protein expression) and then combine the dissimilarities from each modality. We reason that combining dissimilarity representations might help to naturally emphasize different information from each modality and maximally capture and infer cell heterogeneity (Figure 1).

To calculate and integrate dissimilarity matrices, we first calculated pairwise Euclidean distances among cells from preprocessed gene or protein expression profiling data. To eliminate potential biases introduced from random noise in ADT signals, we applied a sigmoid filter to distances calculated from protein expression profiles. Second, because the feature scale and dimensions differ between gene and protein expression profiles, we rescaled each pairwise dissimilarity matrix by using a linear transformation to minimize the average sum of squared differences between the dissimilarity matrices. Finally, we integrated the dissimilarity matrices by using the L_∞ norm. Broadly, this is equivalent to choosing the maximal distance between any pair of cells on the basis of either protein or gene expression. In conclusion, we simply maximize the cell-cell dissimilarity by using information from both single-cell mRNA and protein expression profiling data.

To quantitatively evaluate clustering quality, we developed a measurement to assess the cluster purity on both modalities of CITE-seq (Figure 1). Because the RNA modality is on the scale of the whole genome, whereas features of the ADT modality are highly variable and dataset dependent, we adopted an entropy-based metric (ROGUE score) for assessing the purity of single-cell populations by using transcriptome profiles from a previous study (Liu et al., 2020), and developed a quantitative metric for assessing the purity of surface protein profiles. In brief, our ADT scoring method defines the sum of the standard deviations of all ADT features as the diversity index of a group of cells, and quantifies the purity of a specific cluster by comparing its diversity index against the diversity index of the entire dataset (see STAR Methods for details). The value range of both RNA and ADT scores is between 0 and 1, and a higher score indicates higher purity. To quantify cluster purity for CITE-seq data with different numbers of ADT features, we designed an adjustable parameter termed the “rank” of the ADT score. The purpose of the rank is to avoid a few highly variable ADT features being masked by groups of much less variable ADT features. Further, we combined purity scores for multiple modalities using a geometric mean function for an overall assessment of clustering quality. The purity scoring for CITE-seq proposed in this study allows users to confidently evaluate the quality of their clusters and efficiently determine the best clustering algorithm for optimized resolution (Figure 1).

Finally, because the resulting cell-cell dissimilarity matrix is equivalent to a distance matrix, LinQ-View is compatible with most conventional clustering methods, for example, k-means, hierarchical clustering, Louvain, and Fuzzy c-means (Blondel et al., 2008; Butler et al., 2018; Dunn, 1973), and dimension reduction methods, for example, multidimensional scaling

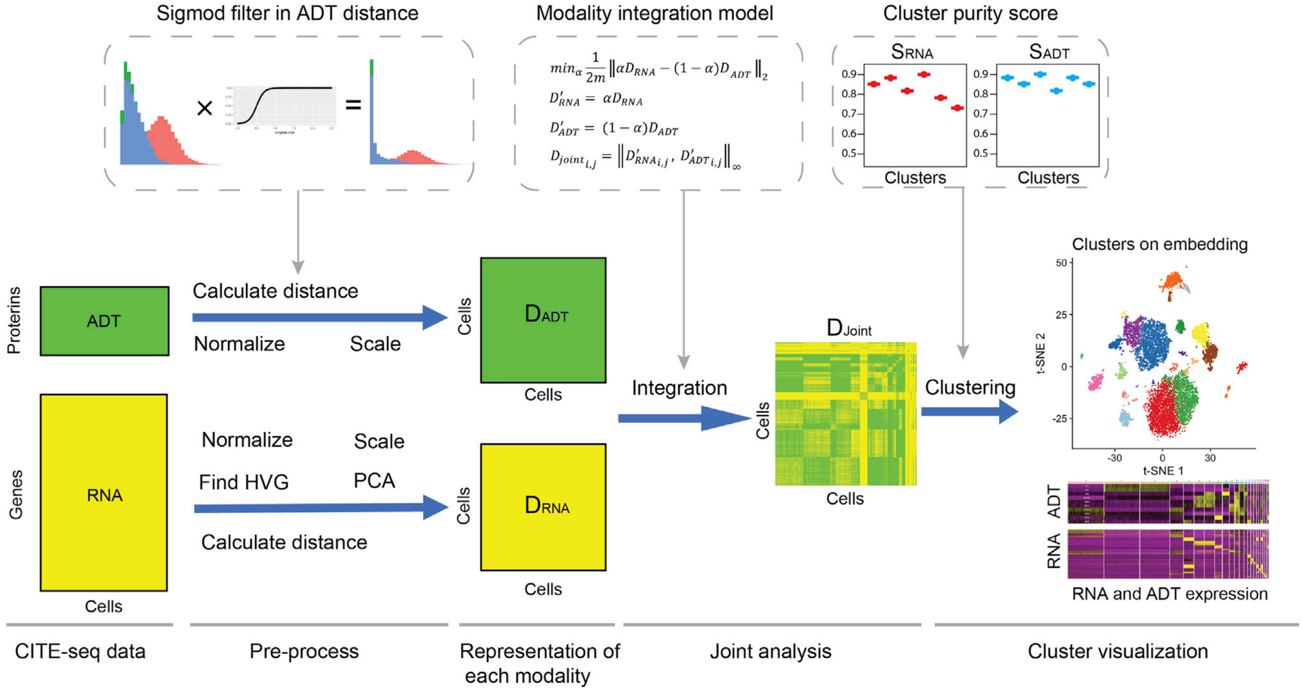

Figure 1. Kernel process of the LinQ-View toolkit

We developed a linear workflow in LinQ-View. Raw expression data of each modality were processed by several pre-processing steps and then were used to calculate cell-cell distances. Specifically, the ADT distances were processed by using a sigmoid filter. Two distance matrices were scaled by a linear transformation and integrated into a joint distance matrix by using an L-infinity norm model. Cell heterogeneity was then inferred from the joint distance matrix. Purity scores for RNA and ADT modalities were used to assess the quality of clustering results and determine proper clustering methods and parameters. Abbreviation are as follows: HVG, highly variable gene; PCA, principal-components analysis.

(MDS), t-distributed stochastic neighbor embedding (t-SNE), and uniform manifold approximation and projection (UMAP) (Buja et al., 2008; McInnes et al., 2018; Van Der Maaten, 2014). The resulting dissimilarity matrix is also compatible with various batch effects correction methods, for example, mutual nearest neighbors (MNN), canonical correlation analysis (CCA), and Seurat anchors (Butler et al., 2018; Haghverdi et al., 2018; Stuart et al., 2019).

Sigmoid filtering prevents variations in a single dominant ADT feature from affecting clustering results in CITE-seq data with limited ADT features

Differing from measurements of RNA modality, which are on the whole-genome scale, ADT modalities are dependent upon the reagents, such as antibodies used in staining. Due to monetary considerations or a specific research focus, most CITE-seq datasets include only a few dataset-specific antibodies that researchers are interested in. Within datasets of this scale, the number of ADT features is usually much smaller than the number of RNA features. Therefore, variations in a single ADT feature have a much higher impact on the distance calculation than those in a single gene. This phenomenon could result in a failure to distinguish cells that have only a few genes differentially expressed when using both modalities and is particularly pronounced for CITE-seq datasets with fewer ADT features.

To demonstrate this phenomenon, we show that results generated directly from RNA- and ADT-distance matrices without a sigmoid filter failed to correctly distinguish memory and naive $CD4^+$ T cell subsets (Figure 2A; Dataset 1). As there are only 10 ADT features measured in this dataset, variations in ADT features become dominant and mask the real pattern in RNA modality (a few differentially expressed genes). To eliminate such bias in ADT features, we introduced a sigmoid filter into the distance calculation of the ADT modality. In brief, we added a penalty to the difference of each ADT feature between any cell pair to reduce the influence of variation of each single ADT feature. The penalty is a sigmoid function, which keeps larger distances essentially constant while further reducing smaller distances. As a result, the sigmoid function filters out minor differences from ADT modalities. Results using the same dataset showed that the LinQ-View model with the sigmoid filter successfully distinguished two $CD4^+$ T cell subsets (Figure 2B). We found that, without the sigmoid filter, the majority of the distance between these two $CD4^+$ T cell subsets is contributed by the ADT modality (ADT, 71.2%; RNA, 28.8%), whereas with the sigmoid filter, the distance between these two $CD4^+$ T subsets is more influenced by the RNA modality (ADT, 43.6%; RNA, 56.4%) (Figure 2C). These results demonstrate that the sigmoid filter is able to correctly distinguish the two $CD4^+$ T subsets that have a minor difference in gene expression without being affected by variation in ADT features. Effectively, the sigmoid filter reduces the weights of small distances from the ADT modality, minimizing the interference from variations of a few ADT features.

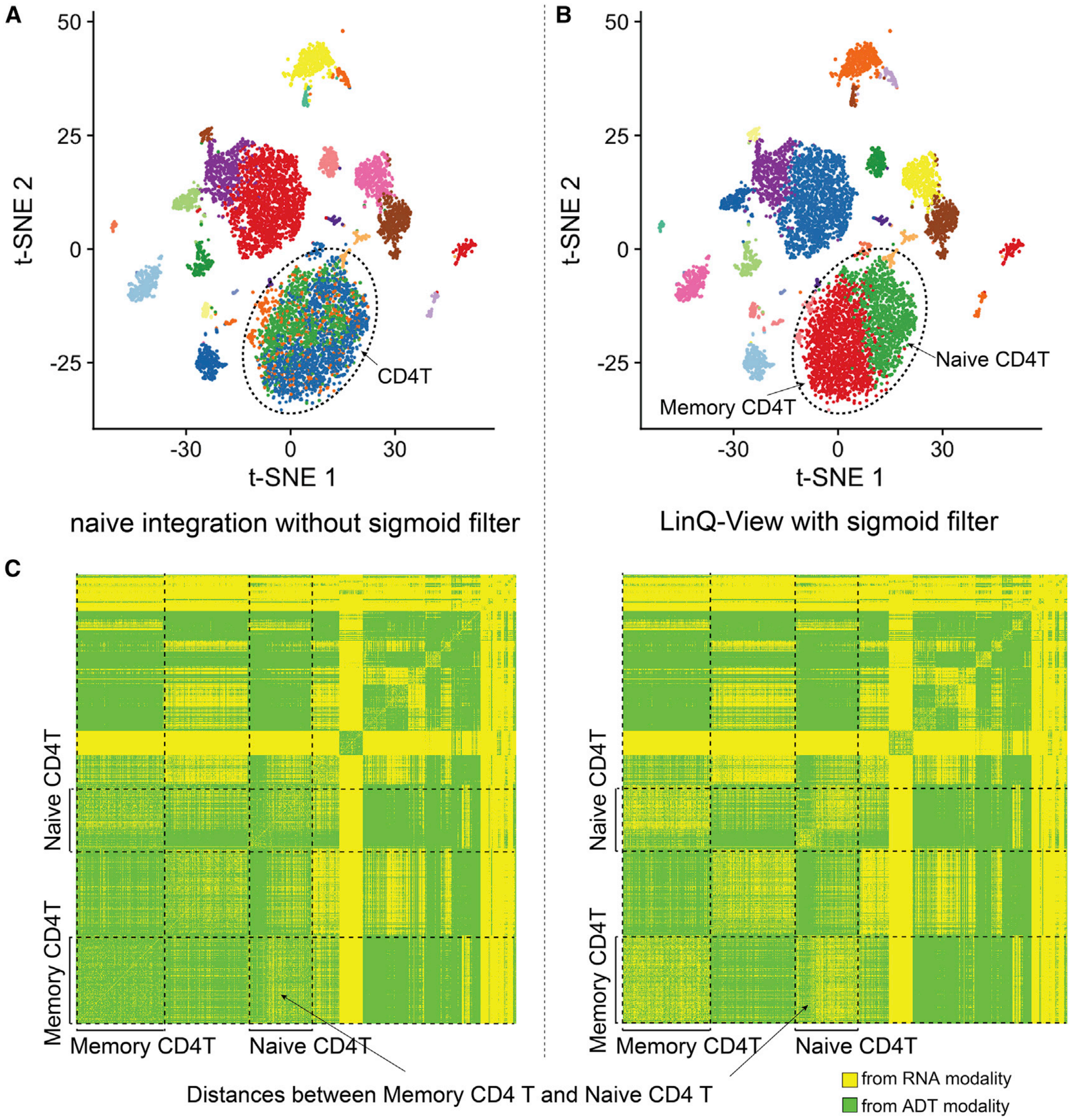

Figure 2. The sigmoid filter prevents variations in a single dominant ADT feature from affecting clustering results

We investigated the effectiveness of the sigmoid filter in preventing variations in a few individual dominant ADT features from affecting clustering results using a real CITE-seq dataset (Dataset 1).

(A and B) Clustering using the LinQ-View model with (B) and without (A) the sigmoid filter. Two models achieve identical results on the majority of the dataset except for CD4$^+$ T cells. A naive integration without the sigmoid filter failed to distinguish naive CD4$^+$ T and memory CD4$^+$ cell subsets (indicated by a dashed circle).

(C) RNA modality contribution of distance between memory CD4$^+$ T and naive CD4$^+$ T cells with and without sigmoid filtering.

CITE-seq purity scoring accurately quantifies the purity of cell clusters

We further tested the effectiveness of our purity scoring method in quantifying cluster purity on both real and simulated datasets. Given that the effectiveness of ROGUE scoring has been demonstrated in a previous study (Liu et al., 2020), we focused on a variance-model-based ADT scoring method. First, we investigated the performance of the ADT scoring method under different ranks by using a real CITE-seq dataset (Dataset 1). Results showed that ADT scores with a proper rank (rank = 2 or 3) can balance the highly variable groups (e.g., clusters 12, 15, 17) in the context of less variable groups, and accurately quantify the differences among groups with different variation levels; ADT scores with too high of a rank (rank = 4, 5) emphasize the difference between highly variable groups (e.g., cluster 12, 15, 17) and other groups, and ignores the difference among mid- to less variable groups (e.g., clusters 0–4, 7–10, 18–22); ADT scores with a rank that is too low (rank = 1) fail to emphasize the overall variation because of the lowest value range (Figures 3A and 3B). Taken together, these results demonstrate that ADT scoring

CellPress
OPEN ACCESS

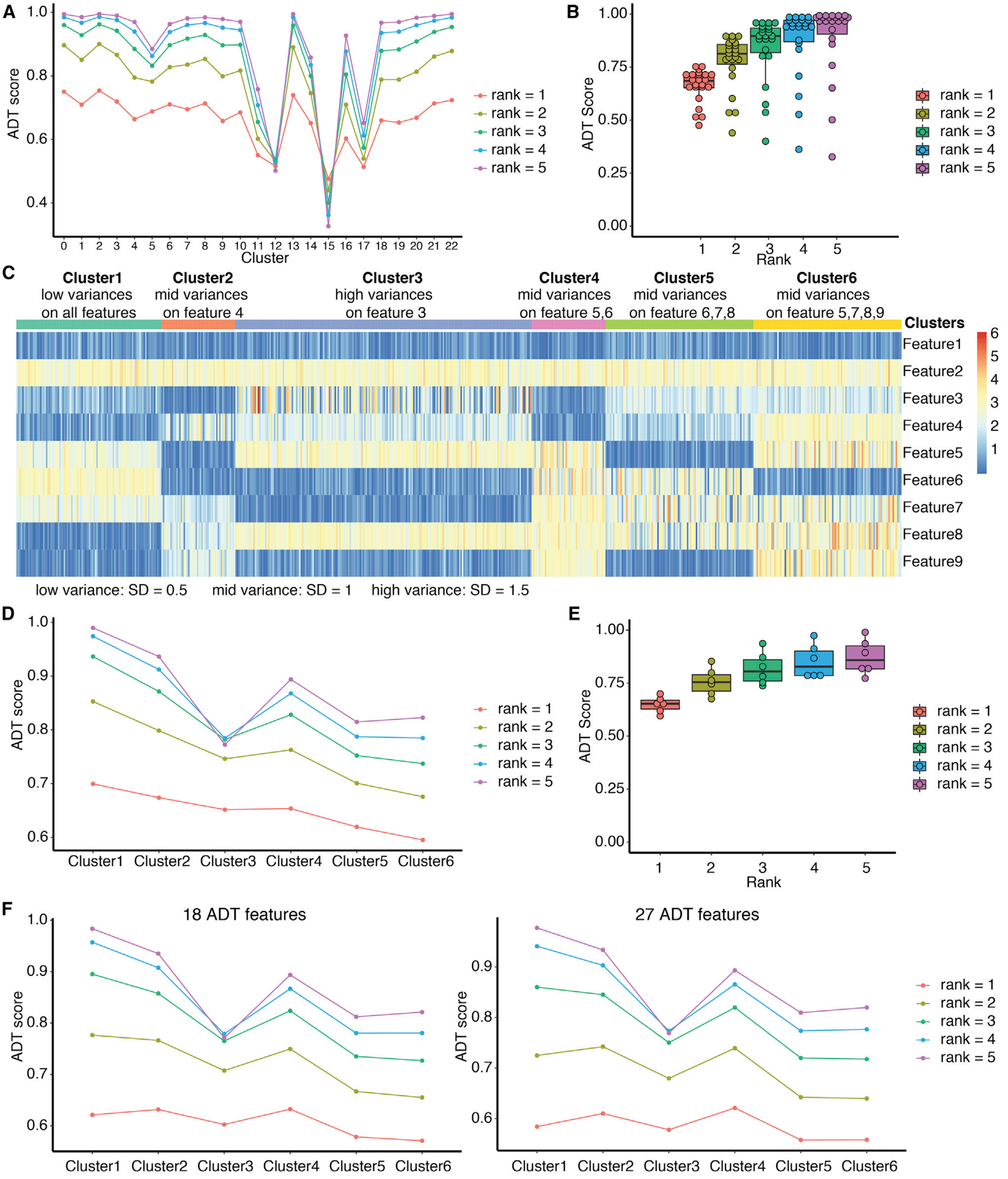

Figure 3. Performance of ADT scoring on both real and simulated datasets

(A) ADT scores under different ranks.

(B) Distributions of ADT scores under different ranks shown by boxplot.

(legend continued on next page)

can highlight a few highly variable groups from other groups, whereas lower ranked ADT scores are less sensitive to variability and generate scores with only minor differences between maximum and minimum scores.

To further investigate ADT scoring, we generated a simulated dataset to demonstrate its performance on clusters with (1) different variation levels and (2) different feature sizes. The simulated dataset comprises six clusters and nine features (Figure 3C). We designed the six clusters with different variation levels to better test the effectiveness of ADT scoring on quantifying ADT variation. Briefly, cluster 1 has low variation on all features, cluster 3 has high variation on one feature; and clusters 2, 4, 5, and 6 have moderate variation on one, two, three, or four features, respectively (see STAR Methods for details). Using this approach, an effective ADT scoring system should be able to accurately quantify the different variation levels of all clusters. Our results showed that under all test ranks, our ADT scoring method was able to accurately quantify the variation level of six clusters: ADT scores decreased in order of the number of variation features per cluster: clusters 1, 2, 4, 5, and 6 (Figures 3D and 3E). The ADT scores of cluster 3 (high variation on one feature) were always lower than those of cluster 2 (mid variation on one feature). We also observed that the higher rank model preferred to highlight variation in a single feature (rank = 5, cluster 3), enabling sensitive detection from a large number of features (Figure 3D).

To demonstrate the ability of ADT scoring to capture variation in single or a few features among a large number of features, we added 9 and 18 negative features with low variation to the existing simulated dataset to generate two new simulated datasets (see STAR Methods for details). We then applied ADT scoring to the two new datasets and compared results among the three simulated datasets. Low-ranked ADT scores failed to accurately quantify the variations of clusters from a high number of features (Figure 3F). These results suggest a higher rank is required by datasets with more ADT features: rank = 2 and 3 performed best on the 9 feature dataset, rank = 3 and 4 performed best on the 18 feature dataset, and rank = 4 and 5 performed best on the 27 feature dataset. In conclusion, testing ADT scores on both real and simulated datasets suggested that ADT scoring is able to accurately quantify the variation level of clusters, and the rank of the ADT score should be increased as the number of feature numbers increase. Furthermore, LinQ-View uses a geometric mean to combine the purity scores of two modalities for an overall assessment of cluster purity.

LinQ-view efficiently reveals NK and CD4$^+$ T cell subpopulations

Clustering and visualization of scRNA-seq data can help to identify rare and intermediate subpopulations. We sought to visualize and evaluate cell clustering by using LinQ-View on a public CITE-seq benchmark dataset (Dataset 1, see STAR Methods for details). Visualization of LinQ-View-processed samples revealed all modality-exclusive cell clusters compared with unimodal transcriptome- or surface protein-based analyses. Using unimodal analyses, we could distinguish two subgroups of natural killer (NK) cells (CD8$^-$ NK and CD8$^+$ NK) only by ADT-based methods and two subgroups of CD4$^+$ T cells (memory and naive CD4$^+$ T cells) only by RNA-based methods (Figure 4A). In particular, the two ADT-exclusive NK cell subsets had identical expression levels of the CD8A gene but distinct expression levels of CD8 protein (Figures 4B and 4C), and therefore could be distinguished only by using information from protein expression. The two RNA-exclusive CD4$^+$ T cells had slight differences in transcriptome expression and exhibited no difference on 10 tested surface proteins (Figure 4D), and could be distinguished only by using transcriptome expression. However, all four modality-exclusive subsets were resolved with LinQ-View (Figure 4A).

Using the CITE-seq purity score proposed in this study, we also evaluated the quality of LinQ-View clustering results, along with two unimodal clustering results. We found that both LinQ-View and RNA clustering achieved high RNA scores (ROGUE score), whereas ADT clustering achieved lower RNA scores (Figure 4E). Furthermore, several ADT clusters (ADT clusters 6, 9, 13, and 17) had a very low ROGUE score (<0.6), indicating that the gene expression profiles within those clusters were highly diverse. Comparison of ADT scores showed that ADT clustering achieved the highest ADT purity score, followed by LinQ-View clustering, and RNA clustering obtained the lowest score (Figure 4E). More specifically, purity scores of the NK cell subset identified by RNA clustering (RNA cluster 4) were 0.9598 (RNA) and 0.6903 (ADT), whereas purity scores of two NK cell subsets identified by LinQ-View (LinQ-View clusters 5 and 6) were 0.9553 and 0.9434 (RNA) and 0.7822 and 0.8279 (ADT). The purity scores of these NK cell subsets showed that LinQ-View distinguished NK cells into two subsets with high RNA purity scores and increased ADT scores at the same time. ADT clustering performed poorly in the purity test because there were several clusters with diverse gene expression (ADT clusters 6, 9, 13, and 17). Furthermore, we compared LinQ-View with unimodal methods on several public CITE-seq datasets (Figures S1 and S2).

Batch effects correction for multimodal data by using LinQ-View

One of the most challenging aspects of analyzing scRNA-seq data are the processing of multiple samples that have been produced in different batches. To evaluate the compatibility of LinQ-View with previously developed batch effects correction methods, we sought to demonstrate seamless integration with batch correction methods by performing LinQ-View analysis on two datasets of peripheral blood mononuclear cells (PBMCs) from healthy donors (Dataset 2, PBMC 1K, and Dataset 3, PBMC 10K). We performed multimodal analysis on two independent runs: one without batch effects correction as a negative

(C) Heatmap of a simulated dataset.
(D) ADT scores under different ranks of clusters of the simulated dataset.
(E) Distributions of ADT scores under different ranks of clusters of the simulated dataset.
(F) ADT scores under different ranks of clusters of two simulated datasets with 18 features and 27 features.

CellPress
OPEN ACCESS

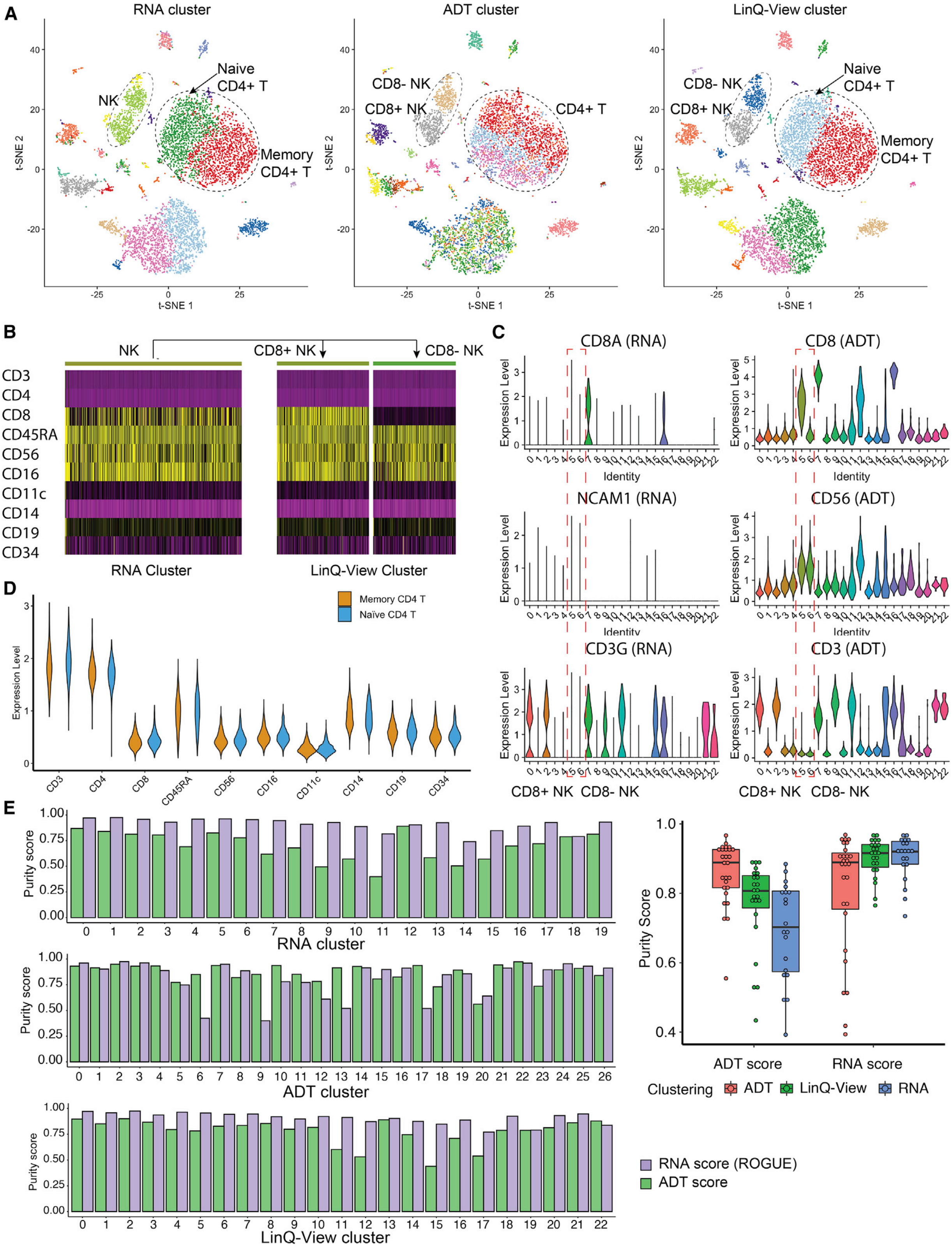

(legend on next page)

control and another with batch effects correction through Seurat 3 (Stuart et al., 2019). Analysis with batch effects correction correctly identified 21 cell groups from the two PBMC datasets, whereas the negative control group identified 24 cell groups, in which four clusters (clusters 11, 12, 16, and 22) were affected by batch effects and were mainly derived from the PBMC 1K dataset (Figures 5A and 5B). Our results showed that LinQ-View is compatible with conventional batch effects correction methods that are designed for unimodal data and is able to identify expected cell populations.

Comparison of LinQ-View with existing multiomics methods in cell heterogeneity identification

To evaluate the effectiveness and robustness of LinQ-View, we compared the use of LinQ-View dissimilarity matrices with three representative published (or pre-released) methods: SNF, MOFA2, and Seurat 4. We compared two unimodal and four multimodal methods on our benchmark dataset. The results of the two unimodal methods, labeled as "RNA cluster" and "ADT cluster," were used as baselines to reveal two CD4$^+$ T cell subsets and two NK cell subsets on t-SNE embedding. The four multimodal clustering methods, LinQ-View, SNF, MOFA2, and Seurat 4, are labeled as "LinQ-View cluster," "SNF cluster," "MOFA2 cluster," and "Seurat 4 cluster," respectively. Using this approach, we demonstrated that LinQ-View and Seurat 4 correctly identified two NK cell subsets and the other two multimodal methods failed to separate any of these subsets at the same resolution (Figure 6A). Notably, MOFA2 successfully distinguished the two NK cell subsets at a higher resolution of 1.5 (Figure S3A). The SNF method failed to correctly separate any of these subgroups at increased resolution (Figure S3B).

We then assessed the cluster quality of different methods by using purity scores (Figures 6B and S3C). Clusters generated by LinQ-View, Seurat 4, and MOFA2 had a higher RNA score than those generated by SNF. MOFA2 (resolution = 0.9), Seurat 4, and SNF all generated one cluster with very low RNA scores, indicating improper clustering. For ADT purity, all methods achieved high ADT scores, and Seurat 4 and LinQ-view achieved the highest. Focusing on two NK cell subsets, all three methods achieved similar RNA scores, whereas LinQ-View and Seurat 4 achieved higher ADT scores than MOFA2 (Figure S3D and Table S1), indicating that LinQ-View, MOFA2, and Seurat 4 are capable of capturing unique patterns of ADT modality. Furthermore, we assessed the quality of subsets of CD4$^+$ T cells generated by these methods. LinQ-View, Seurat 4, and MOFA2 (at resolution = 1.5) identified two, five, and four clusters from CD4$^+$ T cells, respectively (Table S2). Purity test results showed that all methods obtained high overall RNA and ADT scores, whereas Seurat 4 generated a cluster with very low RNA and ADT scores (Seurat cluster 10, Figure 6C). In addition to cluster purity, a further investigation of ADT and RNA expression profiles for these CD4$^+$ T cell subsets generated by three multimodal methods demonstrated that LinQ-View correctly identified naive and memory CD4$^+$ T cell subsets, whereas MOFA2 and Seurat 4 generated a few clusters with similar RNA and ADT expression profiles (Figures 6D and 6E, Tables S3 and S4, see STAR Methods for details), suggesting LinQ-View reveals more accurate clusters for specific cell subsets. Furthermore, we also applied the comparison onto multiple public CITE-seq datasets to demonstrate the generalizability of the LinQ-View method (Figures S4 and S5, see STAR Methods for details).

By comparing the time efficiency of these methods, we demonstrated that LinQ-View and Seurat 4 had a much higher time efficiency than MOFA2, SNF, and CiteFuse (see STAR Methods for details). To better understand the limitations and application range of LinQ-View, we then tested LinQ-View on multiple single-cell datasets with different sizes (Tables S5 and S6). Results suggested that LinQ-View is capable of handling large but not massive single-cell datasets, and the best application range for LinQ-View is between 0 and 50K cells with fewer than 50 ADT features. This application is ideal for users who are specialized in studying distinct cell populations, such as B cells or T cells, which present limited numbers of biologically relevant ADT features.

LinQ-View identifies unique B cell subsets from SARS-CoV-2-infected patients

We have shown the effectiveness of LinQ-View in distinguishing major cell populations (e.g., T cells, B cells, and NK cells) on several public datasets (Figures 4, S1, S2, S4, and S5). To further demonstrate the utility of LinQ-View, we applied this pipeline to peripheral blood CD19$^+$ B cells from 10 SARS-CoV-2-infected COVID-19 patients (Dataset 6). LinQ-View successfully identified several unique B cell subsets with similar transcriptional expression but distinct profiles for the five tested surface proteins. RNA clusters 0 and 1 (indicated by dashed circle with label "a"), two naive-like B cell subsets, were further divided into four subsets by LinQ-View: they are LinQ-View clusters 0, 3, 4, and 5 (Figure 7A). LinQ-View cluster 0 is negative to all tested ADTs (non-responder group), cluster 3 is positive to all tested ADTs (polyreactive group), cluster 4 represents an HLA-DR$^+$ group, and cluster 5 is CD62L$^+$ (Figure 7B). These four subsets are indistinguishable on RNA-based t-SNE because they have highly similar transcriptional expression. In addition, RNA cluster 2 (indicated by dashed circles with label "b") was divided into two subsets: LinQ-View cluster 2, which is HLA-DR$^-$, and cluster 10, which is HLA-DR$^+$ (Figure 7B). These two subsets were also

Figure 4. LinQ-View achieves more precise cell clustering than conventional unimodal methods

We applied LinQ-View and two unimodal analyses (RNA cluster and ADT cluster) on a CITE-seq dataset (Dataset 1).

(A) Clustering results of three methods were visualized on the same t-SNE embedding. Two NK cell subsets and two CD4$^+$ T cell subsets (naive and memory) are indicated by dashed circles.

(B) Expression profiles of 10 tested surface proteins of a single NK cell subset identified by RNA clustering, and two NK cell subsets identified by LinQ-View.

(C) CD8a, NCAM1, and CD3G gene transcription levels and CD8, CD56, and CD3 protein expression levels for two NK subsets (indicated by red dashed boxes).

(D) Expression profiles of 10 tested surface proteins of two CD4$^+$ T cell groups identified by RNA clustering and LinQ-View clustering.

(E) Comparison of purity scores among two unimodal clusterings and LinQ-View clustering.

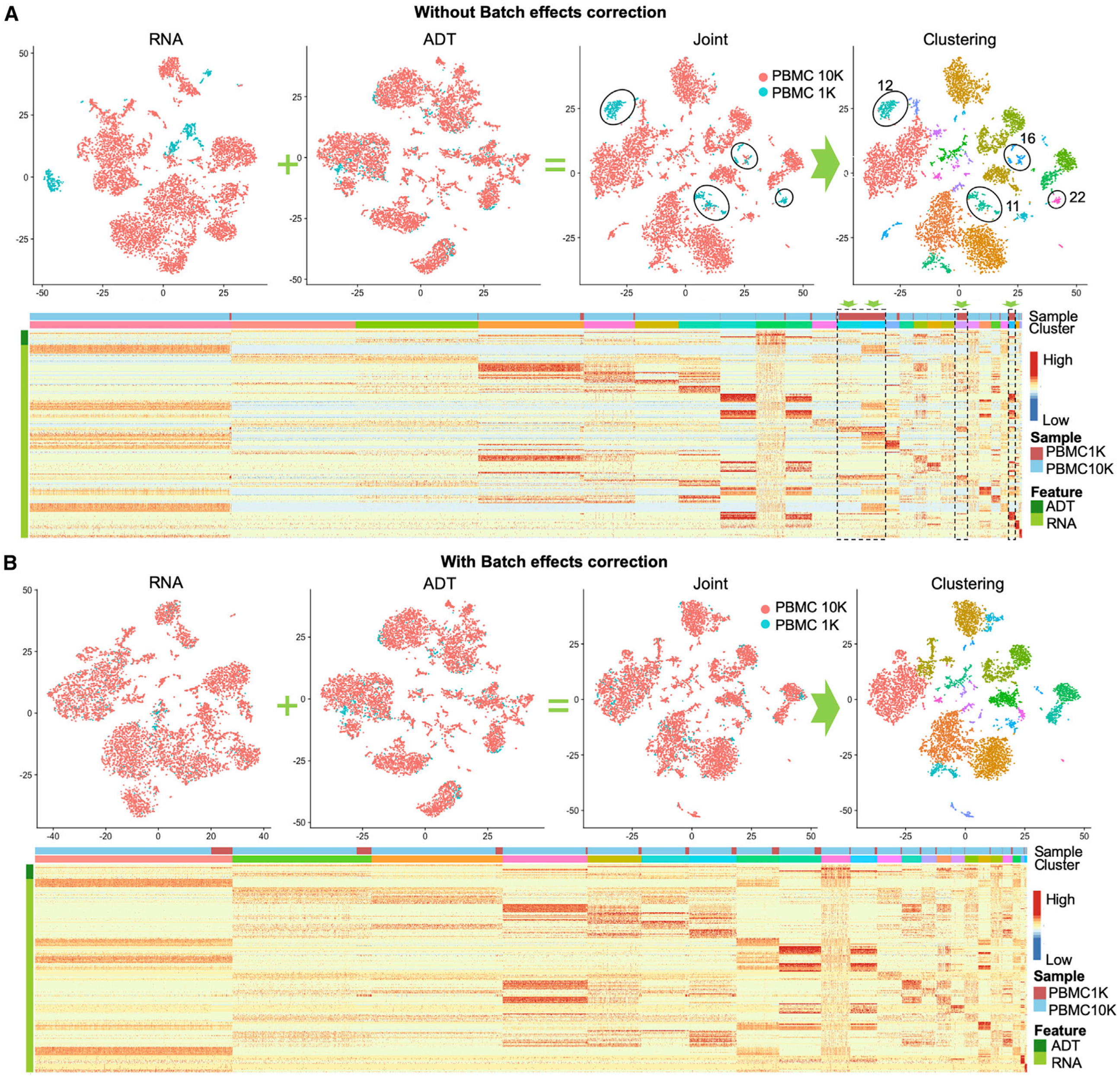

Figure 5. Batch effect correction for multimodal data with LinQ-View analysis

We applied LinQ-View on two CITE-seq datasets (PBMC 1K, Dataset 2; PBMC 10K, Dataset 3). Batch effects were removed by Seurat 3, and two modalities were integrated by LinQ-View. Heatmaps of ADT and RNA for cell populations identified from LinQ-View analysis with batch effect correction are shown on the bottom of (A) and (B). From left to right, four t-SNE embeddings were generated using RNA, ADT, joint, and joint dissimilarity matrix, respectively.

(A) A negative control without batch effect removal. Four cell populations affected by batch effects are indicated by green arrows and black dashed rectangles in the heatmaps.

(B) LinQ-View analysis with batch effect correction.

indistinguishable by transcriptional expression (Figure 7A). Furthermore, LinQ-View identified three unique B cell subsets with distinct ADT profiles (LinQ-View clusters 1, 8, and 11) from RNA clusters 3, 5, and 6 (Figure 7A, indicated by dashed circles with label "c"). LinQ-View cluster 1 was negative to all tested ADTs, cluster 8 was positive for all tested ADTs, and cluster 11 was HLA-DR^{+} (Figure 7B). As indicated by the dashed circles in t-SNE embeddings, cells in group a (LinQ-View clusters 0, 3, 4, and 5), group b (LinQ-View clusters 2 and 10), and group c (LinQ-View clusters 1, 8, and 11) were mixed together on RNA t-SNE and could not be distinguished by using RNA only, even with higher resolution.

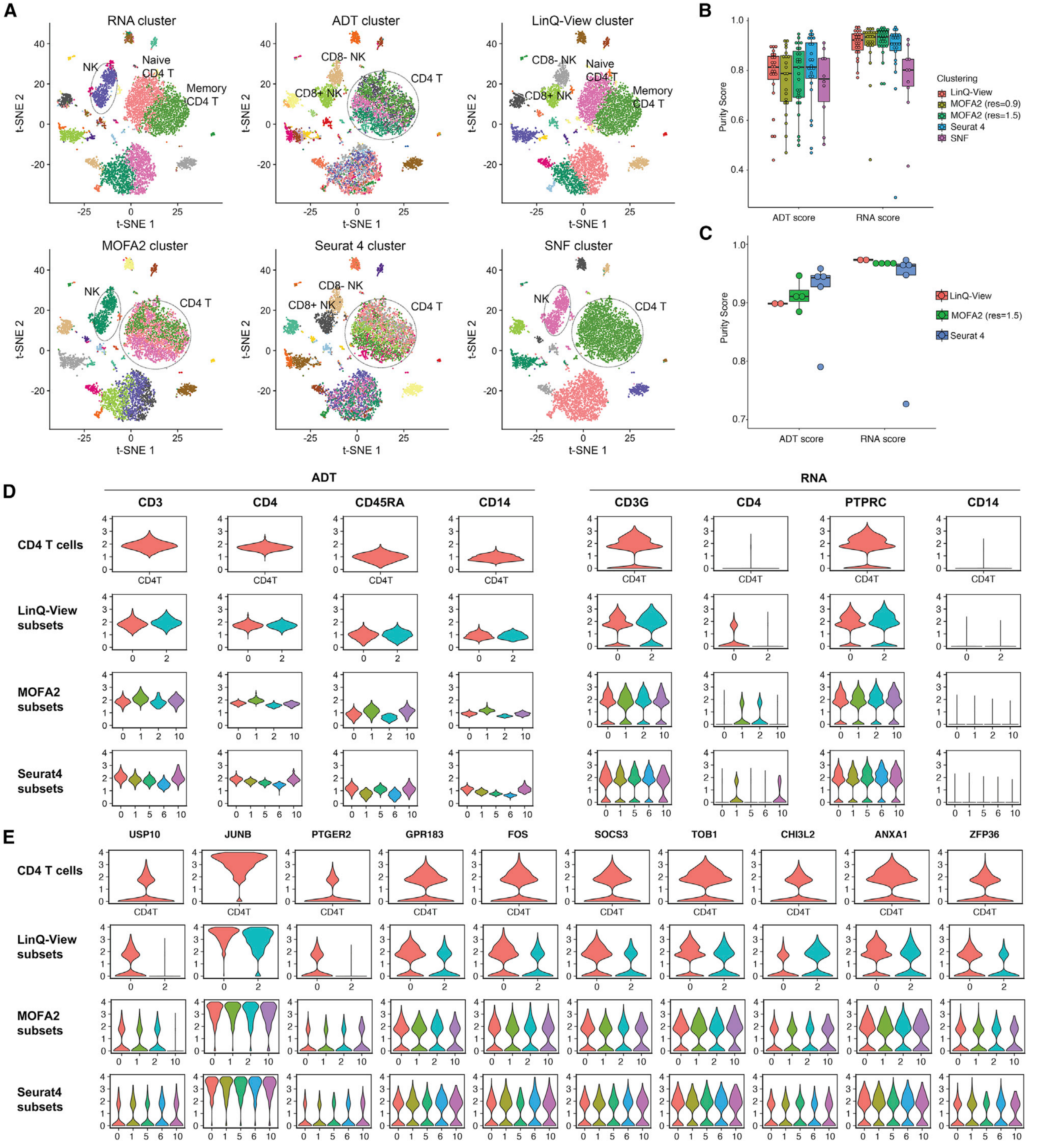

Figure 6. Comparison of cell heterogeneity identification among LinQ-View, MOFA2, Seurat 4, and SNF methods

We compared the LinQ-View method with three multimodal methods, MOFA2, Seurat 4, and SNF, on a CITE-seq dataset. The resolution for Louvain clustering was set to 0.9 if there is no indication.

(A) Clustering using two unimodal and four multimodal methods. Two CD4^{+} T cell subgroups and two NK cell subgroups were highlighted in the t-SNE embedding.

(B) Comparison of purity scores among four multimodal clustering methods.

(legend continued on next page)

Among these unique B cell subsets identified by LinQ-View, clusters 4, 10, and 11 exhibited HLA-DR protein expression and cluster 5 exhibited CD62L protein expression, distinguishing them from transcriptionally similar subsets, which indicates significant phenotypic differences. Notably, some B cells expressed B cell receptors that non-specifically bound to multiple antigens. Clusters 3 and 8 exhibited strong binding to all tested ADTs and COVID-19 probes, suggesting that these two clusters might be composed of polyreactive B cells with non-specific binding features. Polyreactivity has been identified as a common feature of the antigen-specific B cell repertoire, and is defined by an antibody's ability to bind multiple self and foreign antigens, likely because of increased conformational flexibility (Mouquet and Nussenzweig, 2012). We verified the polyreactive nature of a sampling of monoclonal antibodies (mAbs) cloned from cells that exhibited binding to multiple unrelated COVID-19 probes by performing a highly standardized polyreactivity ELISA (Guthmiller et al., 2020). We found that over half of all sampled mAbs were indeed polyreactive (Figure S6A). We next confirmed whether cells exhibiting non-specific binding were reactive to the streptavidin-PE-oligo (SA-PE-oligo) used to conjugate the COVID-19 probes. Ninety percent of sampled mAbs also reacted to SA-PE-oligo, confirming the non-specific nature of these cells (Figure S6B). Given that clusters 3 and 8 displayed normal percentages of mitochondrial gene expression and similar transcriptional expression profiles compared with other B cells, they were indistinguishable for conventional transcriptome-based unimodal methods (Figures 7C and 7D). However, due to the non-specific nature of these cells and our inability to assess whether and how they participate meaningfully in an immune response, we concluded that these clusters should be excluded from analysis. Future studies are warranted to investigate the role of polyreactive B cells in antibody responses to COVID-19.

By investigating immunoglobulin repertoires from these subsets, we identified additional differences on the basis of variable (V) gene locus, isotype, and degree of somatic hypermutation (SHM), which could further discern the identity of these B cell subsets (Figure 7E). Clusters 0, 2, 3, 4, 5, 10, 12, and 14 had the lowest median number of SHMs (0) and highest IgM/D proportion (>95%) and are thus most likely to be naive-like B cells. In contrast, clusters 6, 7, and 9 displayed an intermediate number of mutations and increased class switching to IgA/G (20%–70%) and so are most likely class-switched memory B cells from more recent immune responses to the SARS-CoV-2 proteins (Figure 7E). Clusters 1, 8, 11, and 13 displayed an increased median number of mutations and increased IgA/G proportion (30%–80%) and are most likely longer-lived memory B cells cross-reacting to the SARS-CoV-2 proteins or that had reacted early in the immune response and so have become more terminally differentiated. We also clustered B cells into six "probe hit" groups according to their COVID-19 antigen probe binding signals by using an existing approach (Dugan et al., 2021). These results revealed that spike-specific B cells were enriched in three memory-like B cell subsets (clusters 6, 7, and 9), with an increased number of SHMs, further supporting the notion that clusters 6, 7, and 9 are derived from a recent immune response to the SARS-CoV-2 proteins. Through this analysis, we were able to detect functional heterogeneity in the B cells that react to SARS-CoV-2 antigens after COVID-19 infection. Studies in our group are ongoing to better understand this functional heterogeneity (Dugan et al., 2021).

DISCUSSION

In this study, we use a simple and intuitive approach based on pairwise distances to represent variations between cells in multiple modalities. By applying LinQ-View to several CITE-seq datasets, we prove the effectiveness of harnessing variations in pairwise distances for maximally inferring cell heterogeneity. To assess the purity of single-cell populations using surface protein expression, we proposed a variance-based purity metric and demonstrated that this ADT scoring was capable of accurately quantifying the purity of single-cell populations on the basis of their ADT variability, and thus could be used to determine the best clustering algorithms for optimal resolution. We provided an empirical formula for users to choose the best rank for their data according to the number of ADT features found in their datasets. Notably, the geometric mean function utilized in our scoring method can be easily extended to datasets with more modalities in the future.

Compared with other multimodal methods, LinQ-View has unique advantages when analyzing CITE-seq data with a routine number (e.g., fewer than 50) of ADT features. Although the latest technology is now capable of measuring up to 228 ADT features in a single experiment, and it is likely that this number will grow in the near future, CITE-seq performed on less than 30,000 cells with a medium number of ADT (e.g., 20–50) will still be a valuable tool, especially for groups with smaller budgets or focusing on specific cell populations. As advances in CITE-seq technology significantly lower the barrier of entry, allowing more laboratories to adopt CITE-seq to investigate their research questions and verify their hypotheses, entry-level CITE-seq will be able to satisfy their research needs. Furthermore, many laboratories focus on only specific cell populations, for example, B cells or T cells, which are relatively small in number and have few associated ADT features of interest. Therefore, a small panel of ADT features of interest is more economical than a full panel of all ADT features for such research topics. Considering both performance and time efficiency, Seurat 4 is the best option for massive CITE-seq datasets with a high number of ADT features, and LinQ-View is the best option on large datasets with a limited number of ADT features (e.g., <50). In conclusion, LinQ-View provides an effective and efficient option, especially for CITE-seq data with limited ADT features for the R community.

(C) Comparison of purity scores in distinguishing CD4$^+$ T cells.
(D) Comparison of subsets of CD4$^+$ T cells generated by LinQ-View, MOFA2, and Seurat 4 on the expression of four ADT markers and their corresponding genes.
(E) Comparison of subsets of CD4$^+$ T cells generated by LinQ-View, MOFA2, and Seurat 4 on the expression of 10 differentially expressed genes between naive CD4$^+$ T and memory CD4$^+$ T subsets identified by RNA-based clustering.

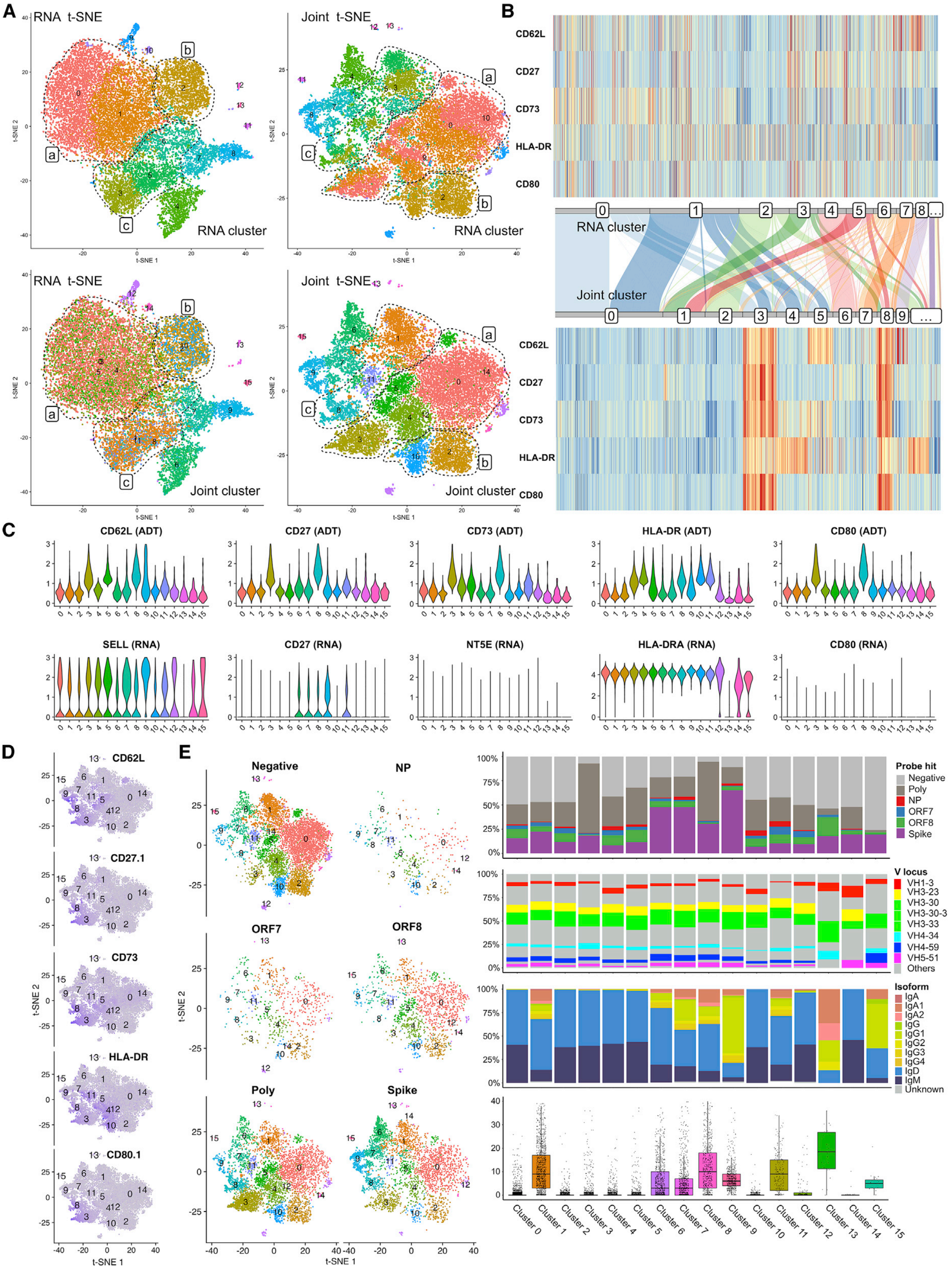

(legend on next page)

The ultimate goal of single-cell methods is to elucidate both temporal and spatial aspects of cells, including spatial distribution, growth history, and interactions with other cells/molecules. In the future it is likely that more modalities will be accessed within a single experiment. To date, six modalities, including transcriptome, surface protein, T cell receptor (TCR) $\alpha\beta$, TCR $\gamma\delta$, sample identity from cell hashtag, and sample identity from single-guide RNA, can be identified by the expanded CRISPR-compatible CITE-seq for single immune cells (Mimitou et al., 2019). However, such multimodal profiling technologies pose new challenges for integrative computational analysis. A robust and extendable system is critical for handling the growing number of modalities. LinQ-View captures variations among cells by pairwise distances, which can be readily extended to multiple new modalities.

Limitations of study

The integration model in LinQ-View calculates distances between all pairs of cells, and then combines them into an integrated distance matrix. Because the memory usage and complexity of pairwise distance calculation grow quadratically as the size of datasets increases, the current design of LinQ-View has a limitation in time efficiency with increasing cell numbers. We have tested the time efficiency of LinQ-View on multiple datasets and have suggested the best application range of this method.

STAR★METHODS

Detailed methods are provided in the online version of this paper and include the following:

- KEY RESOURCES TABLE
- RESOURCE AVAILABILITY
  - ○ Lead contact
  - ○ Materials availability
  - ○ Code availability
- METHOD DETAILS
  - ○ Datasets
  - ○ Simulated datasets for ADT modality
  - ○ Pipeline design
  - ○ Cell quality control
  - ○ Integrating transcriptome and cell surface proteins
- PURITY SCORE OF CELL CLUSTERS FOR CITE-SEQ DATA
  - ○ Parameter setting in model comparison
  - ○ Time efficiency test
  - ○ Data pre-processing
  - ○ ADT and RNA expression for CD4+ T cell subsets generated by three multimodal methods on Dataset 1
  - ○ Comparison of LinQ-View with existing multi-omics methods on multiple CITE-seq datasets
  - ○ Time efficiency test of existing multimodal methods
- QUANTIFICATION AND STATISTICAL ANALYSIS

SUPPLEMENTAL INFORMATION

Supplemental information can be found online at https://doi.org/10.1016/j.crmeth.2021.100056.

ACKNOWLEDGMENTS

This project was funded in part by the National Institute of Allergy and Infectious Diseases (NIAID); National Institutes of Health (NIH) grants U19AI082724 (P.C.W.), U19AI109946 (P.C.W.), and U19AI057266 (P.C.W.); and NIAID Centers of Excellence for Influenza Research and Surveillance (CEIRS) grant HHSN272201400005C (P.C.W.). N.W.A. was supported by the Multi-disciplinary Training Program in Cancer Research (MTCR), NIH T32 CA009594. A.J. and R.P.J. were supported by federal funds from the NIAID, NIH, and Department of Health and Human Services under contract HHSN272201700060C. F.K was funded by NIAID CEIRS contract HHSN272201400008C, Collaborative Influenza Vaccine Innovation Centers (CIVIC) contract 75N93019C00051, and the generous support of the JPB Foundation, the Open Philanthropy Project (2020-215611), and other philanthropic donations. D.F. and C.N. were supported by NIAID contracts HHSN272201700060C and 75N93019C00062. We would like to thank Dr. Congting Ye for his assistance and suggestions for this project.

AUTHOR CONTRIBUTIONS

L.L. designed the model, implemented the package, performed computational analyses of single-cell data, and wrote the manuscript. H.L.D. and C.S. collected samples, designed and performed experiments, and wrote the manuscript. L.Y-L.L. performed experiments and revised the manuscript. N.W.A. generated VDJ, 5′transcriptome, and feature libraries; performed Illumina sequencing; and revised the manuscript. M.K. helped in model design and revised the manuscript. N.-Y.Z. collected samples, expressed recombinant SARS-CoV-2 proteins, and generated mAbs. O.S. performed serum ELISAs and assisted in mAb generation. M.L.M., M.O.J., and K.S. coordinated the convalescent COVID-19 clinical study and collected patient samples. S.C. collected samples, performed ELISAs, and expressed recombinant SARS-CoV-2 proteins. H.A.U. collected samples and expressed recombinant SARS-CoV-2 proteins. C.H. revised the manuscript. C.N., R.P.J., D.F., A.J., and F.K. provided recombinant SARS-CoV-2 proteins. J.H. provided funding and resources for N.A. to perform sequencing and revised the manuscript. A.A.K. and P.C.W. supervised the work and wrote the manuscript.

DECLARATION OF INTERESTS

The authors declare no competing interests.

Figure 7. LinQ-View analysis identifies unique B cell subsets from COVID-19 cohorts

(A) B cell subsets identified by RNA only (top two plots) and LinQ-View analysis (bottom two plots), displayed on RNA t-SNE (left two plots) and on LinQ-View t-SNE (right two plots). Three major groups of B cell subsets are indicated by dashed circles with labels a, b, and c.

(B) Protein expression of RNA clusters and LinQ-View clusters. Only the eight largest RNA clusters and the nine largest LinQ-View clusters are indicated by their IDs due to limited space. A Sankey diagram shows the relationships between RNA clusters and LinQ-View clusters.

(C) Expression levels of five tested cell-surface proteins and their corresponding genes for 16 cell clusters identified by LinQ-View.

(D) Visualization of cell-surface protein expression on t-SNE embedding.

(E) COVID-19 antigen probe binding and B cell repertoire of B cell subsets identified by LinQ-View. Cells were clustered into six different probe hit groups according to their probe binding profiles. Four bar plots, from top to the bottom, show the proportion of probe hit, the proportion of V gene usage, the proportion of isoform, and the number of somatic hypermutation of each cluster, respectively. The four bar plots share the same x axis.

Received: February 22, 2021
Revised: May 12, 2021
Accepted: June 25, 2021
Published: July 23, 2021

REFERENCES

Argelaguet, R., Arnol, D., Bredikhin, D., Deloro, Y., Velten, B., Marioni, J.C., and Stegle, O. (2020). MOFA+: a statistical framework for comprehensive integration of multi-modal single-cell data. Genome Biol. *21*, 1–17.

Argelaguet, R., Velten, B., Arnol, D., Dietrich, S., Zenz, T., Marioni, J.C., Buettner, F., Huber, W., and Stegle, O. (2018). Multi-Omics Factor Analysis—a framework for unsupervised integration of multi-omics data sets. Mol. Syst. Biol. *14*, e8124.

Blondel, V.D., Guillaume, J.-L., Lambiotte, R., and Lefebvre, E. (2008). Fast unfolding of communities in large networks. J. Stat. Mech. Theor. Exp. *2008*, P10008.

Buja, A., Swayne, D.F., Littman, M.L., Dean, N., Hofmann, H., and Chen, L. (2008). Data visualization with multidimensional scaling. J. Comput. Graphical Stat. *17*, 444–472.

Butler, A., Hoffman, P., Smibert, P., Papalexi, E., and Satija, R. (2018). Integrating single-cell transcriptomic data across different conditions, technologies, and species. Nat. Biotechnol. *36*, 411–420.

Cao, J., Packer, J.S., Ramani, V., Cusanovich, D.A., Huynh, C., Daza, R., Qiu, X., Lee, C., Furlan, S.N., Steemers, F.J., et al. (2017). Comprehensive single-cell transcriptional profiling of a multicellular organism. Science *357*, 661–667.

Dugan, H.L., Stamper, C.T., Li, L., Changrob, S., Asby, N.W., Halfmann, P.J., Zheng, N.-Y., Huang, M., Shaw, D.G., and Cobb, M.S. (2021). Profiling B cell immunodominance after SARS-CoV-2 infection reveals antibody evolution to non-neutralizing viral targets. Immunity *54*, 1290–1303.e7.

Dunn, J.C. (1973). A Fuzzy relative of the ISODATA process and its use in detecting compact well-separated clusters. J. Cybernetics *3*, 32–57.

Guthmiller, J.J., Lan, L.Y.-L., Fernández-Quintero, M.L., Han, J., Utset, H.A., Bitar, D.J., Hamel, N.J., Stovicek, O., Li, L., and Tepora, M. (2020). Polyreactive broadly neutralizing B cells are selected to provide defense against pandemic threat Influenza viruses. Immunity *53*, 1230–1244.e5.

Haghverdi, L., Lun, A.T., Morgan, M.D., and Marioni, J.C. (2018). Batch effects in single-cell RNA-sequencing data are corrected by matching mutual nearest neighbors. Nat. Biotechnol. *36*, 421.

Hao, Y., Hao, S., Andersen-Nissen, E., Mauck, W.M., Zheng, S., Butler, A., Lee, M.J., Wilk, A.J., Darby, C., and Zagar, M. (2020). Integrated analysis of multimodal single-cell data. Cell *184*, 3573–3587.

Hashimshony, T., Wagner, F., Sher, N., and Yanai, I. (2012). CEL-seq: single-cell RNA-seq by multiplexed linear amplification. Cell Rep. *2*, 666–673.

Kim, H.J., Lin, Y., Geddes, T.A., Yang, J.Y.H., and Yang, P. (2020). CiteFuse enables multi-modal analysis of CITE-seq data. Bioinformatics *36*, 4137–4143.

Klein, A.M., Mazutis, L., Akartuna, I., Tallapragada, N., Veres, A., Li, V., Peshkin, L., Weitz, D.A., and Kirschner, M.W. (2015). Droplet barcoding for single-cell transcriptomics applied to embryonic stem cells. Cell *161*, 1187–1201.

Liu, B., Li, C., Li, Z., Wang, D., Ren, X., and Zhang, Z. (2020). An entropy-based metric for assessing the purity of single cell populations. Nat. Commun. *11*, 1–13.

Macosko, E.Z., Basu, A., Satija, R., Nemesh, J., Shekhar, K., Goldman, M., Tirosh, I., Bialas, A.R., Kamitaki, N., Martersteck, E.M., et al. (2015). Highly parallel genome-wide expression profiling of individual cells using nanoliter droplets. Cell *161*, 1202–1214.

McInnes, L., Healy, J., and Melville, J. (2018). UMAP: uniform manifold approximation and projection for dimension reduction. arXiv, 1802.03426.

Mimitou, E.P., Cheng, A., Montalbano, A., Hao, S., Stoeckius, M., Legut, M., Roush, T., Herrera, A., Papalexi, E., and Ouyang, Z. (2019). Multiplexed detection of proteins, transcriptomes, clonotypes and CRISPR perturbations in single cells. Nat. Methods *16*, 409–412.

Mouquet, H., and Nussenzweig, M.C. (2012). Polyreactive antibodies in adaptive immune responses to viruses. Cell Mol. Life Sci. *69*, 1435–1445.

Peterson, V.M., Zhang, K.X., Kumar, N., Wong, J., Li, L., Wilson, D.C., Moore, R., McClanahan, T.K., Sadekova, S., and Klappenbach, J.A. (2017). Multiplexed quantification of proteins and transcripts in single cells. Nat. Biotechnol. *35*, 936–939.

Picelli, S., Bjorklund, A.K., Faridani, O.R., Sagasser, S., Winberg, G., and Sandberg, R. (2013). Smart-seq2 for sensitive full-length transcriptome profiling in single cells. Nat. Methods *10*, 1096–1098.

Ramsköld, D., Luo, S., Wang, Y.-C., Li, R., Deng, Q., Faridani, O.R., Daniels, G.A., Khrebtukova, I., Loring, J.F., Laurent, L.C., et al. (2012). Full-length mRNA-Seq from single-cell levels of RNA and individual circulating tumor cells. Nat. Biotechnol. *30*, 777–782.

Rosenberg, A.B., Roco, C.M., Muscat, R.A., Kuchina, A., Sample, P., Yao, Z., Graybuck, L.T., Peeler, D.J., Mukherjee, S., Chen, W., et al. (2018). Single-cell profiling of the developing mouse brain and spinal cord with split-pool barcoding. Science *360*, 176–182.

Shen, R., Olshen, A.B., and Ladanyi, M. (2009). Integrative clustering of multiple genomic data types using a joint latent variable model with application to breast and lung cancer subtype analysis. Bioinformatics *25*, 2906–2912.

Shen, R., Wang, S., and Mo, Q. (2013). Sparse integrative clustering of multiple omics data sets. Ann. Appl. Stat. *7*, 269.

Stoeckius, M., Hafemeister, C., Stephenson, W., Houck-Loomis, B., Chattopadhyay, P.K., Swerdlow, H., Satija, R., and Smibert, P. (2017). Simultaneous epitope and transcriptome measurement in single cells. Nat. Methods *14*, 865.

Stuart, T., Butler, A., Hoffman, P., Hafemeister, C., Papalexi, E., Mauck, W.M., Hao, Y., Stoeckius, M., Smibert, P., and Satija, R. (2019). Comprehensive integration of single-cell data. Cell *177*, 1888–1902.e21.

Stuart, T., and Satija, R. (2019). Integrative single-cell analysis. Nat. Rev. Genet. *20*, 257–272.

Tang, F., Barbacioru, C., Wang, Y., Nordman, E., Lee, C., Xu, N., Wang, X., Bodeau, J., Tuch, B.B., Siddiqui, A., et al. (2009). mRNA-Seq whole-transcriptome analysis of a single cell. Nat. Methods *6*, 377–382.

Van Der Maaten, L. (2014). Accelerating t-SNE using tree-based algorithms. J. Machine Learn. Res. *15*, 3221–3245.

Wang, B., Mezlini, A.M., Demir, F., Fiume, M., Tu, Z., Brudno, M., Haibe-Kains, B., and Goldenberg, A. (2014). Similarity network fusion for aggregating data types on a genomic scale. Nat. Methods *11*, 333.

Wang, X., Yao, H., Zhang, Q., Ren, X., and Zhang, Z. (2019). Direct comparative analysis of 10X genomics chromium and smart-seq2. bioRxiv, 615013.

Wolf, F.A., Angerer, P., and Theis, F.J. (2018). SCANPY: large-scale single-cell gene expression data analysis. Genome Biol. *19*, 15.

Zheng, G.X., Terry, J.M., Belgrader, P., Ryvkin, P., Bent, Z.W., Wilson, R., Ziraldo, S.B., Wheeler, T.D., McDermott, G.P., and Zhu, J. (2017). Massively parallel digital transcriptional profiling of single cells. Nat. Commun. *8*, 1–12.

STAR★METHODS

KEY RESOURCES TABLE

REAGENT or RESOURCE	SOURCE	IDENTIFIER
Software and algorithms		
R v3.6.3	The R Foundation	https://www.r-project.org/
RStudio v1.2.1335	RStudio	https://www.rstudio.com/
LinQ-View v0.99	This paper	https://wilsonimmunologylab.github.io/LinQView/
Seurat v3.9.9	Hao et al., 2020	https://satijalab.org/seurat/
dplyr v1.0.2	Hadley Wickham	https://dplyr.tidyverse.org/
ggplot2 v3.3.2	Hadley Wickham	https://ggplot2.tidyverse.org/
Umap v0.2.6.0	McInnes et al., 2018	https://github.com/lmcinnes/umap
Rtsne v0.15	Van Der Maaten, 2014	https://github.com/jkrijthe/Rtsne
ROGUE	Liu et al., 2020	https://github.com/PaulingLiu/ROGUE

RESOURCE AVAILABILITY

Lead contact
Further information and requests for resources should be directed to and will be fulfilled by the Lead Contact, Patrick C. Wilson (wilsonp@uchicago.edu).

Materials availability
This study did not generate new unique reagents.

Code availability
LinQ-View is implemented in R and freely available from the Wilson Lab GitHub (https://github.com/WilsonImmunologyLab/LinQView). Documents and tutorials are available from GitHub pages (https://wilsonimmunologylab.github.io/LinQView)

METHOD DETAILS

Datasets
Human multimodal datasets were obtained from public resources and previously published reports (Datasets 1-6).

Dataset 1: a dataset of 8,617 cord blood mononuclear cells (CBMCs), produced with CITE-seq (Stoeckius et al., 2017). NCBI GEO accession number is GSE100866. Notably, three ADT features were excluded in downstream analysis (CCR5, CCR7, CD10).

Dataset 2: a dataset of 713 peripheral blood mononuclear cells (PBMCs) from a healthy donor stained with TotalSeq-B antibodies. Data is available from 10x Genomics https://support.10xgenomics.com/single-cell-gene-expression/datasets/3.0.0/pbmc_1k_protein_v3 .

Dataset 3: a dataset of 7,865 peripheral blood mononuclear cells (PBMCs) from a healthy donor stained with TotalSeq-B antibodies. Data is available from 10x Genomics https://support.10xgenomics.com/single-cell-gene-expression/datasets/3.0.0/pbmc_10k_protein_v3.

Dataset 4: a dataset of 8,412 cells from a dissociated Extranodal Marginal Zone B- Cell Tumor (MALT: Mucosa-Associated Lymphoid Tissue) stained with TotalSeq-B antibodies. Data is available from10x Genomics https://support.10xgenomics.com/single-cell-gene-expression/datasets/3.0.0/malt_10k_protein_v3 .

Dataset 5: a dataset of 5,247 peripheral blood mononuclear cells (PBMCs) from a healthy donor stained with TotalSeq-B antibodies. Data is available from 10x Genomics https://support.10xgenomics.com/single-cell-gene-expression/datasets/3.1.0/5k_pbmc_protein_v3 .

Dataset 6: ten datasets of peripheral blood $CD19^+$ B cells that were collected from SARS-CoV-2-infected subjects and stained with TotalSeq-C antibodies. The data are from clinical trial NCT04340050 and registered at ClinicalTrials.gov. Data has been published with a prior study (Dugan et al., 2021) and is available from Mendeley data: https://data.mendeley.com/datasets/3jdywv5jrv/3. Subjects used in this study: S116, S166, S24, S376, S48, S141, S171, S305, S469, and S92. We measured the expression of five cell surface proteins and binding to seven COVID-19 protein probes by flow cytometry: spike protein, spike receptor binding domain (RBD), open reading frames 7a and 8 (ORF7a, ORF8), nucleoprotein (NP) and non-structural proteins (NSPs),

B cell immunoglobulin genes, as well as 5' gene expression in the same immune cells simultaneously. Methodology for antigen probe generation and B cell sorting is described previously(Dugan et al., 2021). We used a pre-titrated cell staining panel that contained oligo-conjugated antibodies against CD62L, CD27, CD73, HLA-DR, and CD80.

Simulated datasets for ADT modality

To better assess the effectiveness of the ADT score proposed in this study, we generated three simulated datasets for ADT modality.

Simulated dataset 1

Simulated dataset 1 is composed of 9 features and 6 clusters. We generated random numbers by following a norm distribution to simulate normalized ADT data. We control the mean and standard deviation (SD) for each cluster and feature to generate data points for clusters with different variation levels. We define SD = 0.5 as low variance, SD = 1 as mid variance, and SD = 1.5 as high variance. We use absolute values in case of negative values. We set the number of cells (data points) for cluster 1–6 to 100, 50, 200, 50, 100, and 100 respectively. Means and SDs for each cluster and each feature has been listed in Table S7.

Simulated dataset 2

Simulated dataset 2 is based on simulated dataset 1. We simply added 9 negative features with low variance (Feature 1 of simulated dataset 1) to simulated dataset 1 to increase the number of features without changing the variance level of all clusters.

Simulated dataset 3

Simulated dataset 3 is based on simulated dataset 1. We simply added 18 negative features with low variance (Feature 1 of simulated dataset 1) to simulated dataset 1 to increase the number of features without changing the variance level of all clusters.

Pipeline design

The LinQ-View pipeline is composed of two major stages: pre-processing and joint analysis. The pre-process workflow was inspired by Seurat and other prior studies (Butler et al., 2018; Stuart et al., 2019; Wolf et al., 2018). As shown in Figure 1, pre-processing steps include cell quality control, unwanted gene removal (optional), data normalization (for both transcriptome and surface protein data), data scaling (for both transcriptome and surface protein data), highly variable gene (HVG) identification, linear dimension reduction (principal component analysis, PCA), and determination of the number of principal components (PC). Next, joint analysis was performed, such as calculating cell-cell pair-wise distances for each modality, joint distance calculation, cell population clustering, non-linear dimension reduction and visualization. The default cell population clustering method in LinQ-View is a community detection algorithm implemented in Seurat, and the LinQ-View toolkit is compatible with other clustering algorithms as well. Similarly, for non-linear dimension reduction, in addition to two default algorithms (t-SNE and UMAP), users may use any algorithm. Furthermore, other modalities, e.g. TCR, BCR and cell hashtags, can be applied to the identified cell populations to investigate feature (gene and protein) enrichment or feature-cluster correlation.

Cell quality control

Cell quality control removes all unwanted cells, such as dying cells, cycling cells and cell doublets from the current analysis. Two commonly used cell quality indicators are the number of detected genes and proportion of mitochondrial genes. Too few detected genes may indicate poor library or sequencing quality, whereas too many detected genes may indicate cycling cells or multiplets. In this study, all cells that have less than 200 genes detected or more than 2500 genes detected have been excluded. In addition, a high proportion of mitochondrial genes may indicate dying cells. Determining an appropriate threshold for number of detected genes is easy, but determining the threshold for proportion of mitochondrial genes is relatively difficult. The threshold for the number of detected genes will vary for different single cell techniques due to differences in sequencing depth and sensitivity; as a result, previous studies have summarized empirical values for different techniques (Wang et al., 2019). For the threshold proportion of mitochondrial genes, it is standard to set an arbitrary low threshold to filter out all unwanted cells (e.g. 5% for 10X data). The fixed threshold works for most cases but may remove too many cells in some specific cases. Here, we propose a strategy called soft threshold to determine the threshold of the proportion of mitochondrial genes. A soft threshold was set to the 95th percentile of the current dataset distribution, and the soft threshold was subject to a sealing point as the maximum threshold in the case of particularly poor cell quality. Of note, a recent study suggested that the proportion of mitochondrial genes may vary for different single-cell techniques(Wang et al., 2019). Its results showed that proportions of mitochondrial genes from Smart-seq/Smart-seq2 data (TPM) are much higher (in average 4-fold) than those from 10x data. In this study, a 10% sealing was applied to all datasets.

Integrating transcriptome and cell surface proteins

In this study, we performed joint analysis by integrating cell-cell pairwise distance matrixes from transcriptome expression and surface protein signals. The cell-cell pairwise distance matrices were calculated separately for each modality (in this instance, transcriptome expression and surface protein signals). Because the number of ADT features (usually from 10–50) is typically much less than the number of RNA features (usually 1000–2000 highly variable genes), variants in a single ADT feature have a much higher impact on the distance calculation than those in a single gene. This phenomenon may result in a failure to distinguish some cell groups that only have a few genes differentially expressed (e.g. memory and naïve CD4$^+$ T cells) when using both modalities, because the random noise in ADT features can mask the signal pattern in so few differentially expressed genes. To eliminate the potential bias in ADT features, we introduced a sigmoid filter into the distance calculation of the ADT modality.

We define the sigmoid filter as:

$$f(x) = \frac{1}{1+e^{-n(x-k)}}$$

where x denotes the original distances, n and k are hyperparameters of sigmoid function, and e is Euler's number (we set $e \approx 2.72$ in practice). Then the filtered distances $F(x)$ will be

$$F(x) = xf(x) = \frac{x}{1+e^{-n(x-k)}}$$

and the original equation for Euclidian distance is defined as:

$$D_E(P,Q) = \|P-Q\|_F = \sqrt[2]{\sum_{i=1}^{N}(p_i-q_i)^2}$$

Here, $P=(p_1,p_2,p_3,\ldots,p_N)$ and $Q=(q_1,q_2,q_3,\ldots,q_N)$ are vectors of two cells P and Q; p_i and q_i are the normalized value of the i-th ADT feature of cell P and Q; and N is the number of ADT features; and $\|\bullet\|_F$ denotes the Frobenius Norm. The sigmoid filtered Euclidian distance $D_{E(sigmoid)}$ is defined as:

$$D_{E(sigmoid)}(P,Q) = \|F(P-Q)\|_F = \|(P-Q)f(P-Q)\|_F$$

$$= \sqrt[2]{\sum_{i=1}^{N}[(p_i-q_i)f(p_i-q_i)]^2} = \sqrt[2]{\sum_{i=1}^{N}\left(\frac{|p_i-q_i|}{1+e^{-n(|p_i-q_i|-k)}}\right)^2}$$

After testing with real data and simulated data, the suggested values of parameters n and k were set to $n=10$ and $k = 0.5$. The sigmoid filter is able to eliminate the potential bias from random noise in ADT data. Some sub-cell groups with only a few differentially expressed genes, e.g. memory and naïve $CD4^+$ T cells, could be identified by joint analysis after ADT data was scaled by the sigmoid filter (Figure 2).

In addition, after investigating the distribution of each ADT signal, we proposed a more precise way to distinguish small distances between negative and positive signals (most likely to be accurate distances) from those within negative/positive (most likely due to noise). Similar to the way users can define positive/negative in flow cytometry, users can define a threshold c (suggested value = 1) for each ADT feature to distinguish the positive and negative values. For a small distance (< threshold c), $d = |v_1 - v_2|$, we define $v_{min} = \min(v_1,v_2)$,$v_{max} = \max(v_1,v_2)$. Then, the distance between negative and positive can be easily identified if $v_{min}<c$ and $v_{max}>c$. Finally, distances between negative and positive will be kept as original, and others within negative/positive distances (indicated by green and blue areas below the red dashed line, respectively) will be scaled to a lower level to eliminate the potential bias from noise. In conclusion, we applied a sigmoid filter in our distance calculations to reduce the impacts of minor differences of ADT in the joint model, avoiding potential bias from ADT noise.

After sigmoid filtered Euclidian distances are constructed for ADT data, then the scaled distances for RNA (D'_{RNA}) and ADT (D'_{ADT}) were calculated by the following equations:

$$D'_{RNA} = \alpha D_{RNA}$$

$$D'_{ADT} = (1-\alpha)D_{ADT}$$

where D_{RNA} and D_{ADT} are Euclidian distance matrices calculated from transcriptome expression (RNA) and surface protein signal (ADT). We determine an optimized value of linear operator α by solving the following convex problem:

$$min_{\alpha}\frac{1}{2m}\|\alpha D_{RNA} - (1-\alpha)D_{ADT}\|_2 \qquad subject\ to\ \alpha\in[0,1]$$

where $m = N(N-1)/2$, and N is the number of cells. This convex optimization problem can be solved by a gradient descent algorithm.

After scaling two distances into the same level, we introduced an L_∞ norm model to calculate the joint distance:

$$D_{joint_{i,j}} = \|D'_{RNA}i,j\ ,\ D'_{ADT}i,j\|_\infty = \max\left(D'_{RNA}i,j\ ,\ D'_{ADT}i,j\right), \quad i,j\subset C$$

where $D_{joint_{i,j}}$, $D'_{RNA}i,j$, $D'_{ADT}i,j$ are joint distance, scaled RNA distance, scaled ADT distance between cell i and j, respectively. $\|.\|_\infty$ denotes L_∞ norm. C is the set of all cells.

In addition, the contributions of each modality can be quantified using an L_∞ norm model. The contribution of each modality was roughly quantified by the ratio of the number of distances from this modality to the total number of distances.

$$Contribution_{RNA} = \frac{m - \| D_{joint} - D'_{RNA} \|_0}{m} * 100\%$$

$$Contribution_{ADT} = \frac{m - \| D_{joint} - D'_{ADT} \|_0}{m} * 100\%$$

Here D_{joint}, D'_{RNA}, D'_{ADT} are the lower triangles of the $N \times N$ matrix, so the total number of distances is $m = N(N-1)/2$. $\|.\|_\infty$ denotes L_0 norm, which is equal to the number of non-zero elements.

Of note, by comparing with other conventional norm models (e.g. an L_1 norm model and L_2 norm model), we find that the L_∞ norm model generates more accurate and robust results than any other norm models. In addition, since distances from multiple modalities were scaled into the same level, too few ADT numbers can cause high individual feature weights, resulting in potential errors from ADT random noise. The L_∞ model is able to overcome this challenge, which is difficult for L_1 or L_2 norm model. A comparison between the L_∞ model and the L_1 model demonstrated that the L_∞ model correctly distinguished memory and naïve CD4 T cell subsets whereas the L_1 model failed. After that, cell clustering and non-linear dimension reduction based on joint distances will be able to represent variations in both transcriptome expression and surface protein expression. In summary, the L_∞ norm model takes valuable information for cell heterogeneity identification from both modalities, and is able to avoid potential bias in distance integration caused by too few ADT numbers.

PURITY SCORE OF CELL CLUSTERS FOR CITE-SEQ DATA

To comprehensively assess the purity of multi-modal cell subsets, we developed two purity scores, and , for both modalities in CITE-seq data. In detail, we adopted ROGUE score from a previous study to assess the purity of single cell populations on transcriptome modality, and developed a quantitive metric to assess the purity of single cell populations on surface protein modality. As demonstrated in a previous study, the ROGUE score is able to accurately quantify the purity of single cell populations using transcriptome data. For a given cell population, the ROGUE score calculates the entropy reduction (ds) of each gene using an expression entropy model, and then quantifies the purity of this cluster by a statistic measurement:

$$S_{RNA} = ROGUE = 1 - \frac{\sum_{sig} ds}{\sum_{sig} ds + K}$$

ds denotes entropy reduction of each gene; $\sum_{sig} ds$ denotes the summary of significant *ds*. *K* is a pre-defined parameter and is dependent on single cell sequencing technology.

Distinct from transcriptome modality, which is at the whole genome scale level, the number of features in the surface protein modality of CITE-seq is determined by users and is usually limited, disabling the application of the ROGUE score on the surface protein modality of CITE-seq. We developed a score to access the purity of single cell populations on the surface protein modality by evaluating the variation level among all ADT features of given populations. We firstly calculate the summary of standard deviations among all ADT features of entire dataset as a benchmark. Then we assess the purity of each clusters in this dataset by calculating:

$$S_{ADT} = 1 - \frac{\sum SD^n}{\sum SD^n + K_{ADT}}$$

SD denotes standard deviation of each ADT feature for a given group; *n* denotes a non-negative (n = 1,2,3…) rank that usually goes higher along the raise of number of ADT features and can be chosen by users. An empirical formula (floor of natural logarithm of ADT feature number) could be helpful in choosing rank: rank=floor(ln(N)), N is the number of ADT features. K_{ADT} denotes the summary of standard deviations among all ADT features of entire dataset using the same rank. It is the benchmark variation level of the entire dataset. In this way, S_{ADT} for a population will be between 0 and 1. Similar to S_{RNA}, a higher score indicates better purity. S_{ADT} equals 0.5 if the variation level of a cluster is the same as that of the entire dataset.

We also developed a geometric mean function to combine the two purity scores for an overall assessment of cluster purity. Notably, this metric is also expandable to datasets with multiple modalities in the future by simply increase the rank to number of modalities of new datasets.

$$S = \sqrt{S_{ADT} S_{RNA}}$$

In practice, users can calculate and compare purity scores for multiple clustering results on the same dataset in order to determine the best clustering algorithm and optimized resolution.

Parameter setting in model comparison

We performed clustering analysis using the same algorithm (Louvain clustering, implemented in the Seurat package) under the same resolution (0.9) for all 4 multimodal methods. For easier comparison and identification of cell subsets across different methods, we visualized the results of all six methods on the same t-SNE embedding (LinQ-View t-SNE). Method-specific clustering and

visualization of three multimodal methods can be found in the supplemental information (Figure S7). This dataset was pre-processed by following conventional pre-processing procedures: we performed log normalization for RNA data and CLR normalization for ADT data, selected 2000 highly variable genes for RNA data, performed PCA analysis for RNA data and selected the top 20 PCs for further analysis. Notably, three ADT features were excluded from this analysis (CCR5, CCR7, CD10). More specifically, for the LinQ-View method, Louvain clustering was performed on a joint distance matrix. The joint distance matrix was generated under a default parameter setting. For the SNF model, we set the number of neighbors (K) to 20, a hyperparameter alpha to 0.5 and the number of iterations (T) to 10 as suggested in SNF tutorial. For MOFA2, we adopted default model parameter settings using a ''get_default_model_options'' function and set the number of factors to 15. The mofa2 model was trained on 2000 highly variable genes and all ten ADT features. Then we ran the MOFA2 model using a ''run_mofa'' function and the model converged in 155 iterations. For Seurat 4, the number of PCs for the RNA modality was set to 20 and the number of PCs for the ADT modality was set to 5.

Time efficiency test

All time efficiency tests were performed on a 2019 version iMac with 3.1 GHz Intel Core i5 processor and 64 GB 2400 MHz DDR4 memory, with operating system version macOS Mojave (10.14.6). R environment version is 3.6.3 (for LinQ-View and SNF) and 4.0.3 (for MOFA2 and Seurat 4).

Data pre-processing

Pre-processing steps were adapted from Seurat (Butler et al., 2018; Stuart et al., 2019). Transcriptome data was processed by cell quality control, unwanted gene removal (optional), data normalization, data scaling, highly variable gene (HVG) identification, linear dimension reduction (PCA) and determination of the number of PC. Surface protein data was processed by data normalization and data scaling.

ADT and RNA expression for CD4+ T cell subsets generated by three multimodal methods on Dataset 1

We investigated the ADT and RNA expression profiles for the CD4+ T cell subsets generated by three multimodal methods. Differences among subsets generated by MOFA2 and Seurat4 on CD3, CD4, CD45RA and CD14 were not significant or had a low fold change in expression difference, and these subsets showed no difference in corresponding genes (Figure 6D). This indicates that the CD4+ T cell subsets generated by MOFA2 and Seurat 4 are most likely being compromised by small variability in the expression of a few ADT features rather than real gene or protein expression patterns. An investigation of the expression of 10 differently expressed genes between naïve and memory CD4+ T cells showed that the subsets identified by MOFA2 and Seurat4 show no difference in these gene markers (Figure 6E). Furthermore, the gene markers identified from 5 Seurat clusters were highly similar, suggesting that there is no significant difference among these subsets in gene expression (Tables S3 and S4). Taken together, single dominant ADT features are commonly found in existing CITE-seq data which have a limited number of ADT features (i.e. < 50). Interference from the ADT modality having an adverse effect on the results is common with existing methods, because these methods generate ADT variances directly from normalized ADT counts or PCA results of ADT modality without any further processing. Our results show that the sigmoid filter and model we proposed in this paper works well on CITE-seq data with limited ADT features by preventing variations in a single dominant ADT feature from impacting clustering results.

Comparison of LinQ-View with existing multi-omics methods on multiple CITE-seq datasets

To demonstrate the generalization of LinQ-View method on CITE-seq data, especially CITE-seq data with routine numbers of surface protein features, we further compared LinQ-View with two commonly used multimodal methods: MOFA2 and Seurat 4. In this comparison, we applied all three methods onto two public benchmark CITE-seq datasets generated by 10X genomics (Dataset 2 and 3). All clustering was performed using the Louvain algorithm under the same resolution (0.6 for dataset 2, 0.8 for dataset 3). Purity score rank for ADT modality was set to 3 for both datasets. For easier comparison and locating cell subsets across different methods, we visualized the results of all three methods on the same t-SNE embedding (LinQ-View t-SNE). Parameter settings for all three methods are as same as described in ''Parameter setting in model comparison'' section.

Using this approach, LinQ-View, MOFA2, and Seurat 4 identified 7, 7, and 8 cell clusters from dataset 2, respectively (Figure S4A). Purity scores of cell clusters generated by three methods showed that all three methods achieved high overall purity scores (Figure S4B). MOFA2 achieved highest RNA score but lowest ADT score, and Seurat 4 achieved highest ADT score but lowest RNA score, indicating the imbalance weighting between ADT and RNA modalities. More specifically, MOFA2 weights RNA modality higher than ADT modality, and Seurat 4 weights ADT modality higher than RNA modality on tested dataset. Notably, besides these statistical differences, there are non-trivial differences among the clustering results of the three methods. For easier demonstration, we labeled two groups of cells using red and blue dashed line and label ''a'' and ''b' (Figure S4A). For group ''a'' cells, LinQ-View divided them into four clusters (cluster 1, 2, 4, and 5), MOFA2 divided them into three clusters (cluster 0, 2, and 5), and Seurat 4 divided them into five clusters (cluster 1, 2, 4, 6, and 7). A further investigation of surface protein marker expression demonstrated that LinQ-View clusters have distinct surface protein expression patterns: cluster 1 is CD3+CD4+CD45RO+, cluster 2 is CD3+ CD45RO+, cluster 4 is CD3+CD4+CD45RA+, and cluster 5 is CD16+CD56+CD45RA+ (Figure S4C). Seurat 4 generated similar results as LinQ-View among cell group ''a''. However, there is a minor difference between LinQ-View clusters 1 and 4 and Seurat 4 clusters 2 and 4. Surface protein expression of these clusters showed that LinQ-View clustering is better because LinQ-View cluster 1 and 4 has much more

distinct expression of CD45RA than Seurat 4 cluster 2 and 4. Furthermore, MOFA2 failed to detect the difference of surface protein expression among these cells and generated three clusters with similar surface protein expression patterns. For group "b" cells, Seurat 4 grouped those cells as cluster 6 whereas LinQ-View identified the difference from those cells and clustered them into clusters 5 and 6 (Figure S4A). A further investigation of surface protein and gene marker expression demonstrated that LinQ-View clusters 5 and 6 have distinct protein expression (e.g. CD56, and CD4) and gene expression (e.g. GNLY, NKG7, LST1, and AIF1) (Figure S4D). Notably, MOFA2 also clustered these cells into two individual clusters (clusters 5 and 6), despite that MOFA2 cluster 5 also improperly included some cells from other clusters.

On dataset 3, LinQ-View, MOFA2, and Seurat 4 identified 26, 19, and 19 cell clusters, respectively (Figure S5A). Purity scores of cell clusters generated by three methods showed that all three methods achieved high overall purity scores (Figure S5B). Similarly to dataset 2, MOFA2 achieved highest RNA score but lowest ADT score, and Seurat 4 achieved highest ADT score but lowest RNA score, indicating the imbalance weighting between ADT and RNA modalities. Similarly, LinQ-View, MOFA2, and Seurat 4 also generated different cell clusters on some groups of cells. For easier demonstration, we labeled two groups of cells using red and blue dashed line and label "a" and "b' (Figure S5A). For group "a" cells, LinQ-View and Seurat 4 identified two clusters (LinQ-View cluster 8 and 9, Seurat 4 cluster 14 and 15). A further investigation of surface protein and gene marker expression demonstrated that LinQ-View clusters 8 and 9 have distinct expression profiles on surface protein CD25 and a few gene markers (e.g. TCL1A, IGHD, YBX3, IGHG4, and S100A6) (Figure S5C). However, MOFA2 failed to detect those gene expression differences and clustered these cells into one cluster (MOFA2 cluster 5). For group "b" cells, LinQ-View clustered these cells into two clusters (LinQ-View cluster 1 and 11), MOFA2 clustered these cells into two clusters (MOFA2 cluster 3 and 8), and Seurat 4 clustered these cells into three clusters (Seurat 4 cluster 8, 9, and 11) (Figure S5D). A further investigation of surface protein and gene marker expression demonstrated that the two LinQ-View clusters (clusters 1 and 11) have distinct expression profiles on two surface protein markers (CD25 and CD127) and a few gene markers (e.g. ANXA1, IL7R, SELL, FOS). However, clusters of group "b" cells identified by Seurat 4 and MOFA2 have similar expression profiles on these surface protein and gene markers, indicating these two methods failed to properly integrate information from two modalities.

Taken together, comparison on datasets 2 and 3 demonstrated that all three methods are able to integrate multiple modalities of CITE-seq data and correctly identify major cell populations, moreover, LinQ-View is able to balance contribution between two modalities and reveal more accurate clusters with distinct protein and gene expression profiles compared to other two methods. Combined with the comparison on dataset 1 in the main text, we demonstrated that LinQ-View identified cell subsets accurately and consistently on multiple CITE-seq datasets, and proved the generalizability and applicability of LinQ-View method.

Time efficiency test of existing multimodal methods

We assessed the time efficiency of these multimodal methods. Using the same test dataset (8067 cells after quality control), SNF required 3–4 hours whereas the LinQ-View model ran in approximately 30 seconds. CiteFuse (using the SNF model as kernel) took more than 8 hours, most likely due to the lack of dimension reduction before modality integration. Thus, in addition to poor performance in multimodal integration, time efficiency test results demonstrate that SNF and CiteFuse are not compatible with large-scale single cell CITE-seq datasets. In addition, MOFA2 required 6 minutes to train the MOFA2 model, and Seurat 4 only took 20 seconds on finding multi-model neighbors using the same dataset. These results demonstrated that LinQ-View and Seurat 4 not only performed better in integrating RNA and ADT modalities, but also had a much better time efficiency than MOFA2. However, because distances were computed between all pairs of cells, the model design of LinQ-View has a limitation on time efficiency with increasing cell number. For example, Seurat 4 is able to handle a massive single cell data set of 170K cells with 228 ADT features whereas all of the other existing methods (including LinQ-View) failed. Taken together, time efficiency test results suggested Seurat 4 has the highest efficiency, especially with massive datasets (>50K cells); LinQ-View is efficient on large size datasets (<50K cells); MOFA2 is about 10 times slower than the previous two methods and is able to handle medium size datasets (<20K cells) in a reasonable timeframe; and the SNF model and CiteFuse are too time-consuming to process even medium size datasets.

Notably, we observed that the time efficiency of MOFA2 is highly variable and dataset-dependent when applied MOFA2 on multiple CITE-seq datasets. Runtime of model training step of MOFA2 on datasets 1, 2, 3, 4, and 5 are 385.48, 12.59, 49.17, 59.22, and 72.67 seconds, respectively. Considering dataset 1 (8067 cells, 10 ADTs), dataset 3 (7865 cells, 17 ADTs) and dataset 4 (8412 cells, 17 ADTs) are similarly sized, our results suggest that time efficiency of MOFA2 is highly dataset-dependent.

QUANTIFICATION AND STATISTICAL ANALYSIS

The boxplots and medians were generated using an R package "ggplot2" (Figures 3B, 3E, 4E, 6B, 6C, and 7E). The upper and bottom edges of the box indicate the 25th percentile (Q1) and 75th percentile (Q3), respectively. The range between Q1 and Q3 is defined as the interquartile range (IQR). The horizontal bar in the box indicates the median (Q2). The top edge of the upper vertical line indicates the maximum (Q3+1.5IQR) and the bottom edge of the lower vertical line indicates the minimum (Q1-1.5IQR). The violin plots were generated using an R package "Seurat" (Figures 4C, 4D, 6D, and 6E). We identified differentially expressed genes and proteins between two groups of cells using a Wilcoxon Rank Sum test implemented in "FindAllMarkers" function in an R package "Seurat".

Cell Reports Methods, Volume *1*

Supplemental information

Improved integration of single-cell transcriptome and surface protein expression by LinQ-View

Lei Li, Haley L. Dugan, Christopher T. Stamper, Linda Yu-Ling Lan, Nicholas W. Asby, Matthew Knight, Olivia Stovicek, Nai-Ying Zheng, Maria Lucia Madariaga, Kumaran Shanmugarajah, Maud O. Jansen, Siriruk Changrob, Henry A. Utset, Carole Henry, Christopher Nelson, Robert P. Jedrzejczak, Daved H. Fremont, Andrzej Joachimiak, Florian Krammer, Jun Huang, Aly A. Khan, and Patrick C. Wilson

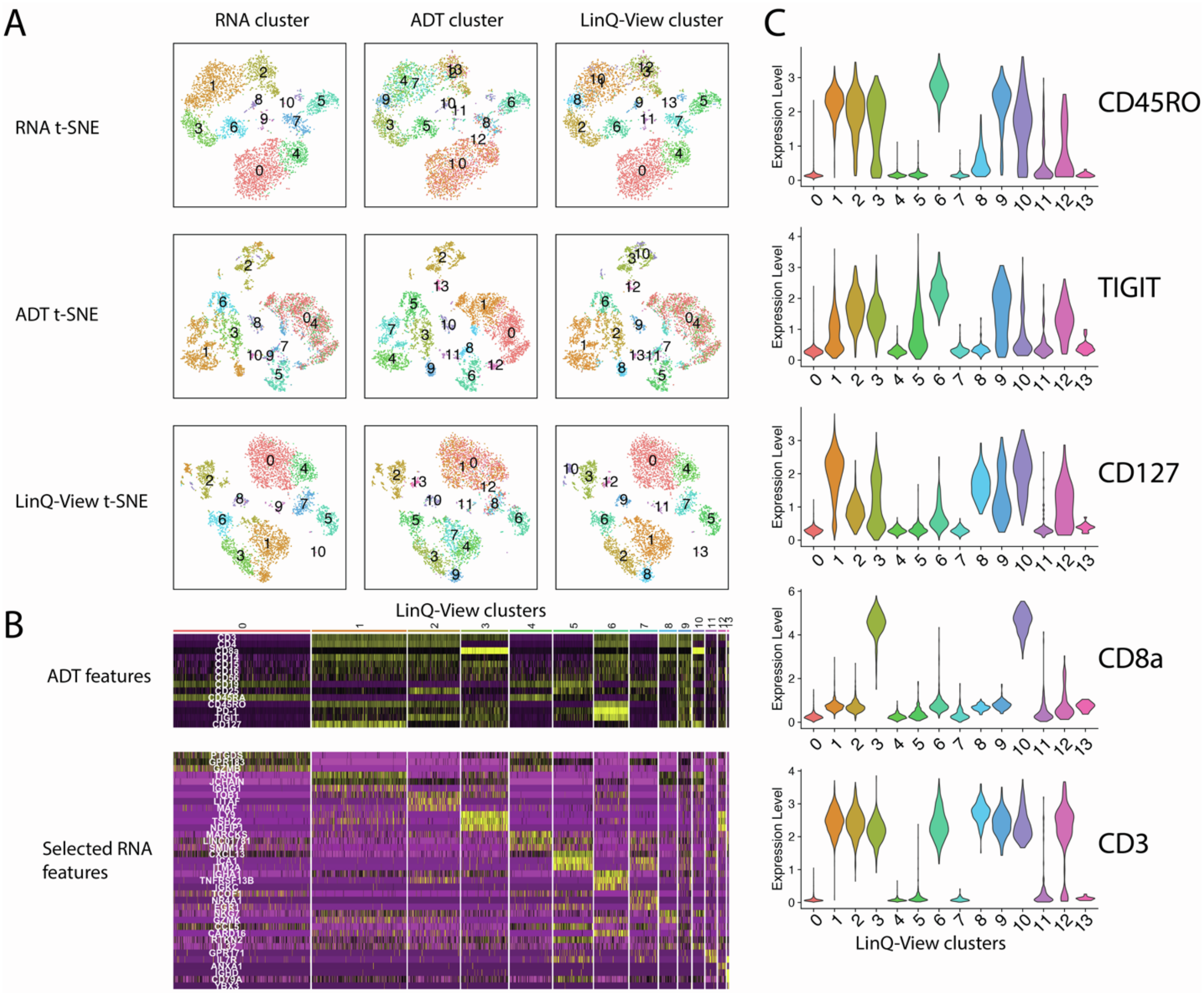

Figure S1. **LinQ-View analysis achieves better cell clustering then conventional unimodal methods by integrating both transcriptome and surface protein signals (Dataset 4), related to Figure 4**. (**A**) All combinations with three different clustering sources and three different cell embeddings are shown in a 3X3 grid. Those clusters were identified using Seurat 3 under a resolution = 0.6 for all three clustering sources (RNA, ADT and LinQ-View). Cells in RNA cluster 1 were divided into two clusters (1, 8, 10) by the LinQ-View method, and cells in RNA cluster 2 were divided into three clusters (3, 12) by the LinQ-View method. LinQ-View clusters 8 can be identified using RNA by increasing resolution to 1.2, whereas LinQ-View cluster 10 and 12 cannot be distinguished by RNA. (**B**) Gene expression profiles on both surface protein level and transcriptome level for cell clusters based on RNA+ADT using LinQ-View. (**C**) Protein expression levels for cell groups identified by LinQ-View method. Cluster 10 has distinct CD8a expression from cluster 1 and 8, cluster 3 and 12 have distinct expression of CD8a protein.

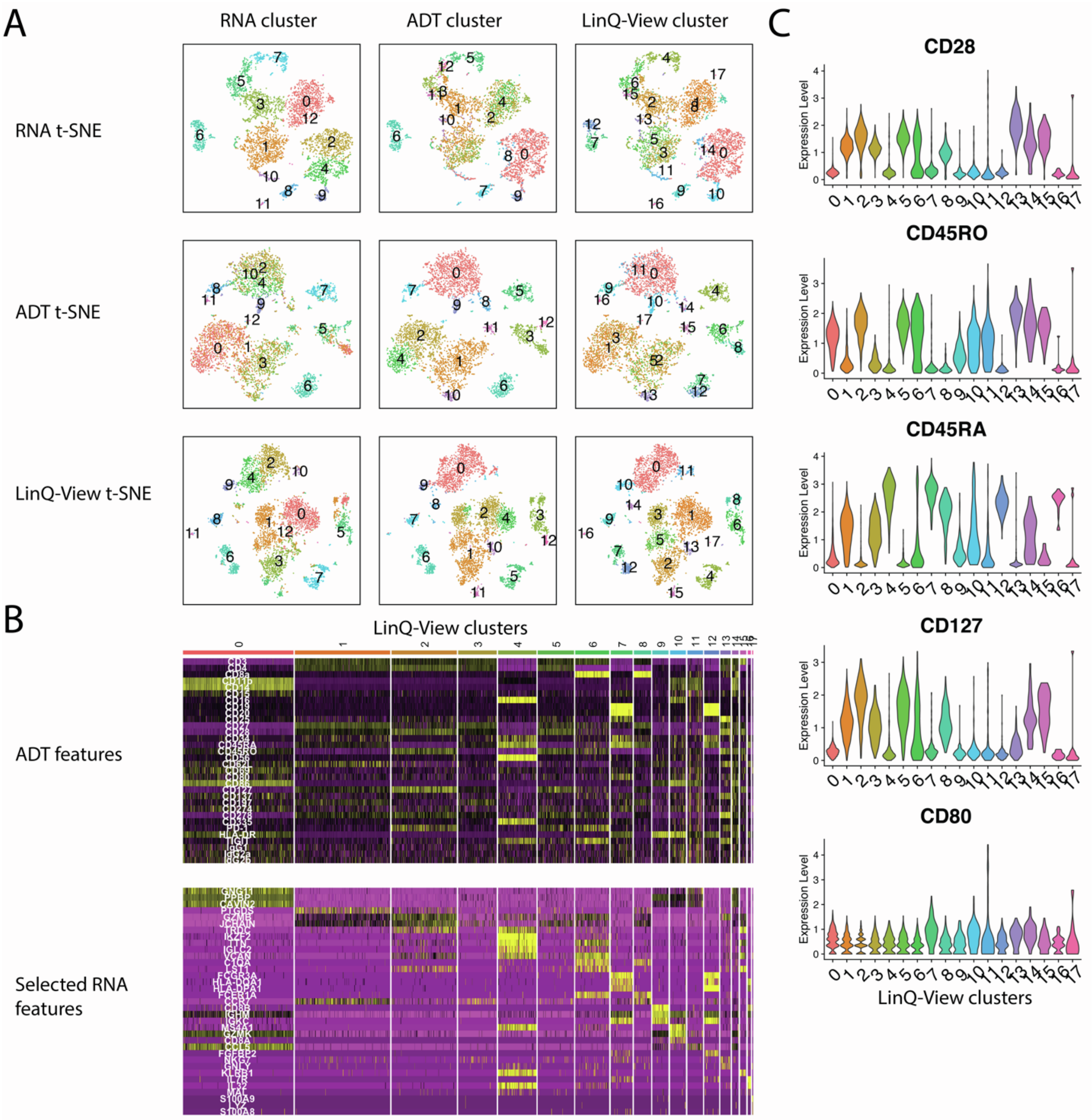

Figure S2. LinQ-View achieves better cell clustering then conventional unimodal methods by integrating both transcriptome and surface protein signals (Dataset 5) , related to Figure 4. (**A**) All combinations with three different clustering sources and three different cell embeddings are shown in a 3X3 grid. Those clusters were identified using Seurat 3 under a resolution = 0.6 for all three clustering sources (RNA, ADT and LinQ-View). As indicated by red and blue circles, cells in RNA cluster 1 were divided into two clusters (3, 5) by the LinQ-View method. (**B**) Gene expression profiles on both surface protein level and transcriptome level for cell clusters based on RNA+ADT using LinQ-View. (**C**) Protein expression levels for cell groups identified by LinQ-View method. Cluster 3 and 5 have distinct expression of CD45RO and CD45RA protein.

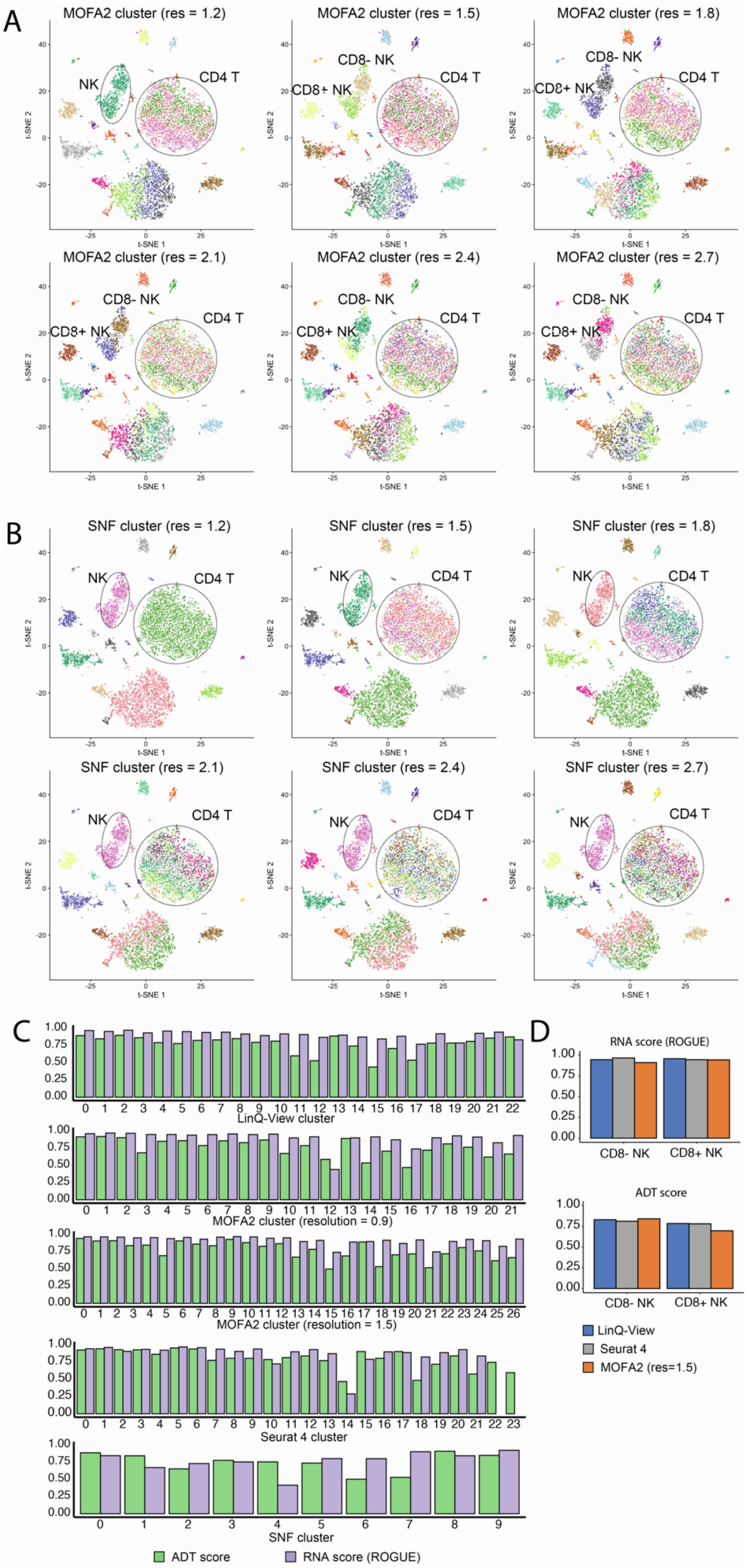
A
MOFA2 cluster (res = 1.2)
MOFA2 cluster (res = 1.5)
MOFA2 cluster (res = 1.8)
MOFA2 cluster (res = 2.1)
MOFA2 cluster (res = 2.4)
MOFA2 cluster (res = 2.7)
NK
CD8- NK
CD8+ NK
CD4 T
t-SNE 1
t-SNE 2
B
SNF cluster (res = 1.2)
SNF cluster (res = 1.5)
SNF cluster (res = 1.8)
SNF cluster (res = 2.1)
SNF cluster (res = 2.4)
SNF cluster (res = 2.7)
C
LinQ-View cluster
MOFA2 cluster (resolution = 0.9)
MOFA2 cluster (resolution = 1.5)
Seurat 4 cluster
SNF cluster
ADT score
RNA score (ROGUE)
D
RNA score (ROGUE)
ADT score
CD8- NK
CD8+ NK
LinQ-View
Seurat 4
MOFA2 (res=1.5)

Figure S3. **Comparing four existing multimodal methods, related to Figure 6.** (**A**) Performance of "MOFA2" method on distinguishing $CD4^+$ T-cell subgroups and NK cell subgroups under a series of increased resolution. (**B**) Performance of "SNF" method on distinguishing $CD4^+$ T-cell subgroups and NK cell subgroups under a series of increased resolution. (**C**) Purity scores among four multimodal clustering methods. (**D**) Comparison of purity scores in distinguishing NK cells among three multimodal methods.

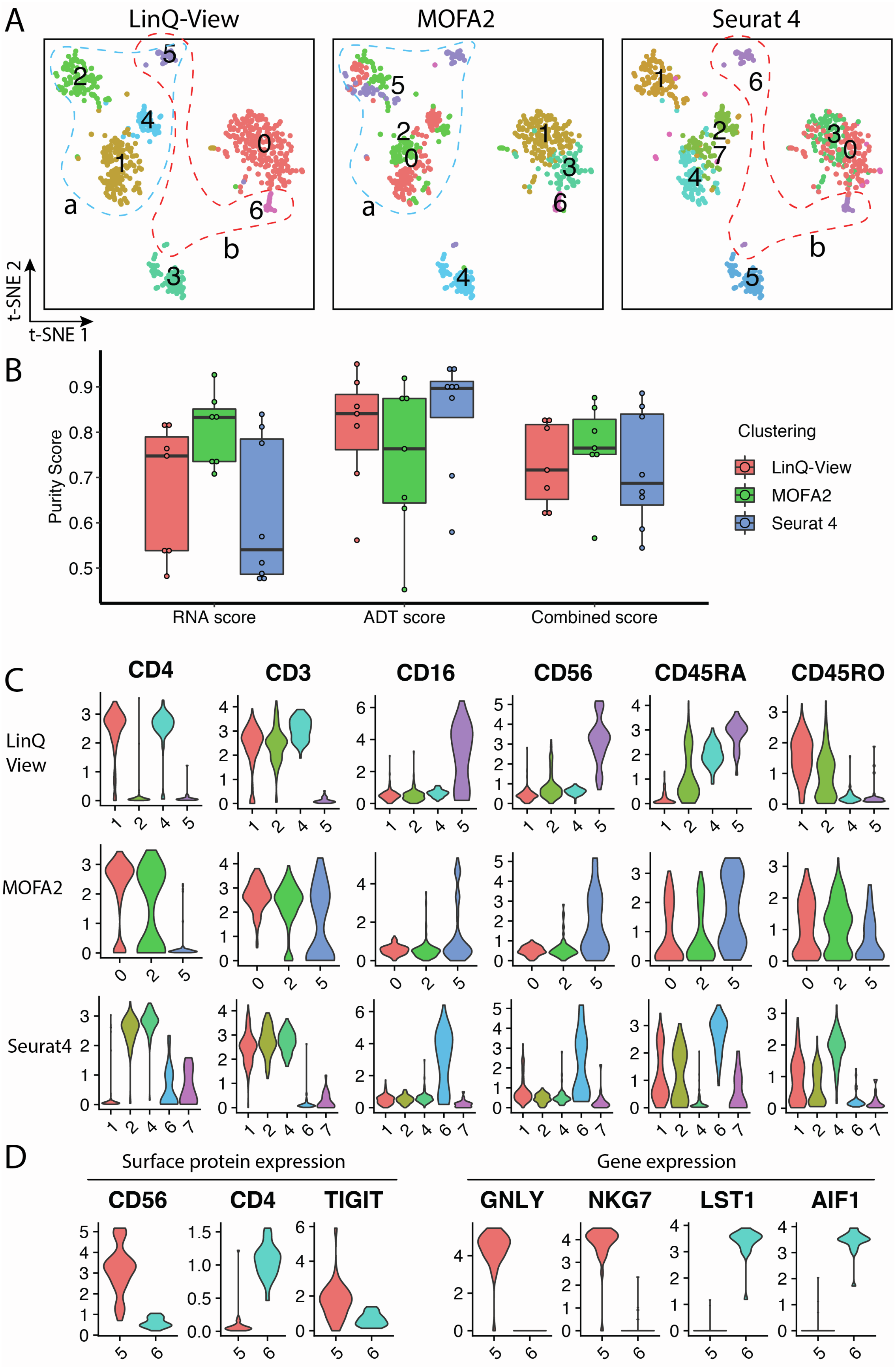
A
LinQ-View
MOFA2
Seurat 4
t-SNE 2
t-SNE 1
B
Purity Score
RNA score
ADT score
Combined score
Clustering
LinQ-View
MOFA2
Seurat 4
C
CD4
CD3
CD16
CD56
CD45RA
CD45RO
LinQ View
MOFA2
Seurat4
D
Surface protein expression
Gene expression
CD56
CD4
TIGIT
GNLY
NKG7
LST1
AIF1

Figure S4. Comparison of cell heterogeneity identification among LinQ-View, MOFA2, and Seurat 4 on dataset 2, related to Figure 6. We compared the LinQ-View method with two existing multimodal methods: MOFA2 and Seurat 4. All methods were applied on a public CITE-seq benchmark dataset (Dataset 2). Resolution for Louvain clustering was set to 0.6. (A) Clustering results of three multimodal methods. All clustering was visualized on the same (LinQ-View) t-SNE embedding. Two groups of cells that will be further discussed were indicated by dashed lines and labels. (B) Purity scores of cell clusters generated by three methods. (C) Comparison of clusters of the group “a” cells generated by LinQ-View, MOFA2, and Seurat4 on the expression of six ADT markers. (D) Comparison of clusters of group “b” cells generated by LinQ-View, MOFA2, and Seurat4 on the expression of three ADT markers and four gene markers.

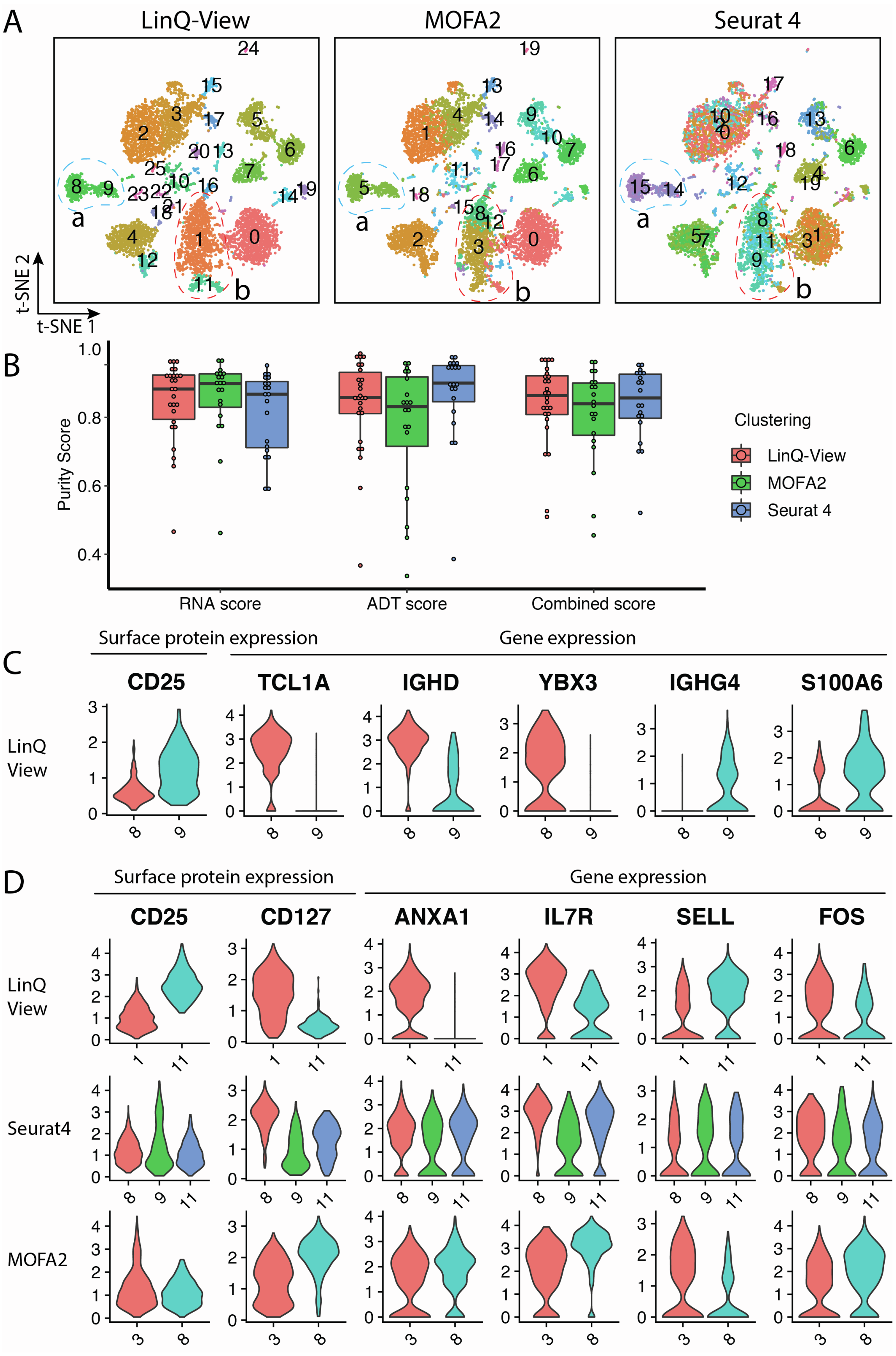
A
LinQ-View
MOFA2
Seurat 4
t-SNE 2
t-SNE 1
B
Purity Score
Clustering
LinQ-View
MOFA2
Seurat 4
RNA score
ADT score
Combined score
C
Surface protein expression
Gene expression
CD25
TCL1A
IGHD
YBX3
IGHG4
S100A6
LinQ View
D
Surface protein expression
Gene expression
CD25
CD127
ANXA1
IL7R
SELL
FOS
LinQ View
Seurat4
MOFA2

Figure S5. Comparison of cell heterogeneity identification among LinQ-View, MOFA2, and Seurat 4 on dataset 3, related to Figure 6. We compared the LinQ-View method with two existing multimodal methods: MOFA2 and Seurat 4. All methods were applied on a public CITE-seq benchmark dataset (Dataset 3). Resolution for Louvain clustering was set to 0.8. (A) Clustering results of three multimodal methods. All clustering was visualized on the same (LinQ-View) t-SNE embedding. Two groups of cells that will be further discussed were indicated by dashed lines and labels. (B) Purity scores of cell clusters generated by three methods. (C) Comparison of clusters of the group “a” cells generated by LinQ-View, MOFA2, and Seurat4 on the expression of one ADT marker and five gene markers. (D) Comparison of clusters of group “b” cells generated by LinQ-View, MOFA2, and Seurat4 on the expression of two ADT markers and four gene markers.

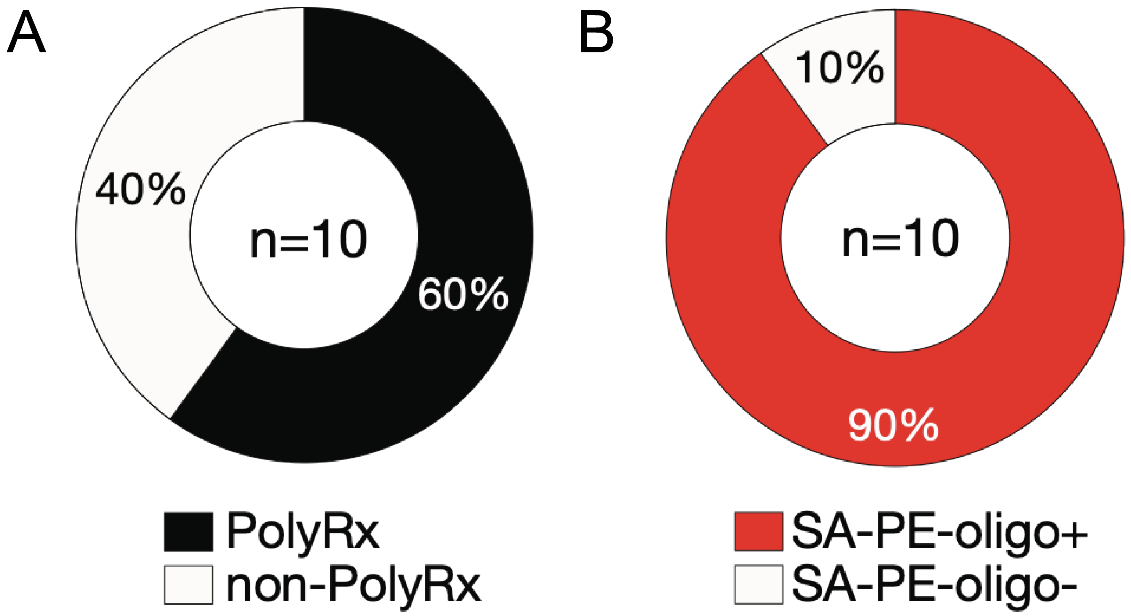

Figure S6. **Experimental characterization of non-specific nature of polyreactive B cells, related to Figure 7.** Monoclonal antibodies (mAbs) identified as multi-probe reactive were tested by ELISA against a panel of standardized antigens (dsDNA, insulin, flagellin, KLH, LPS and cardiolipin in a polyreactivity ELISA assay (A) as well as streptavidin-PE-oligo (SA-PE-oligo) used to conjugated the sorting probes (B). Numbers in the center of each pie chart indicate number of mAbs tested.

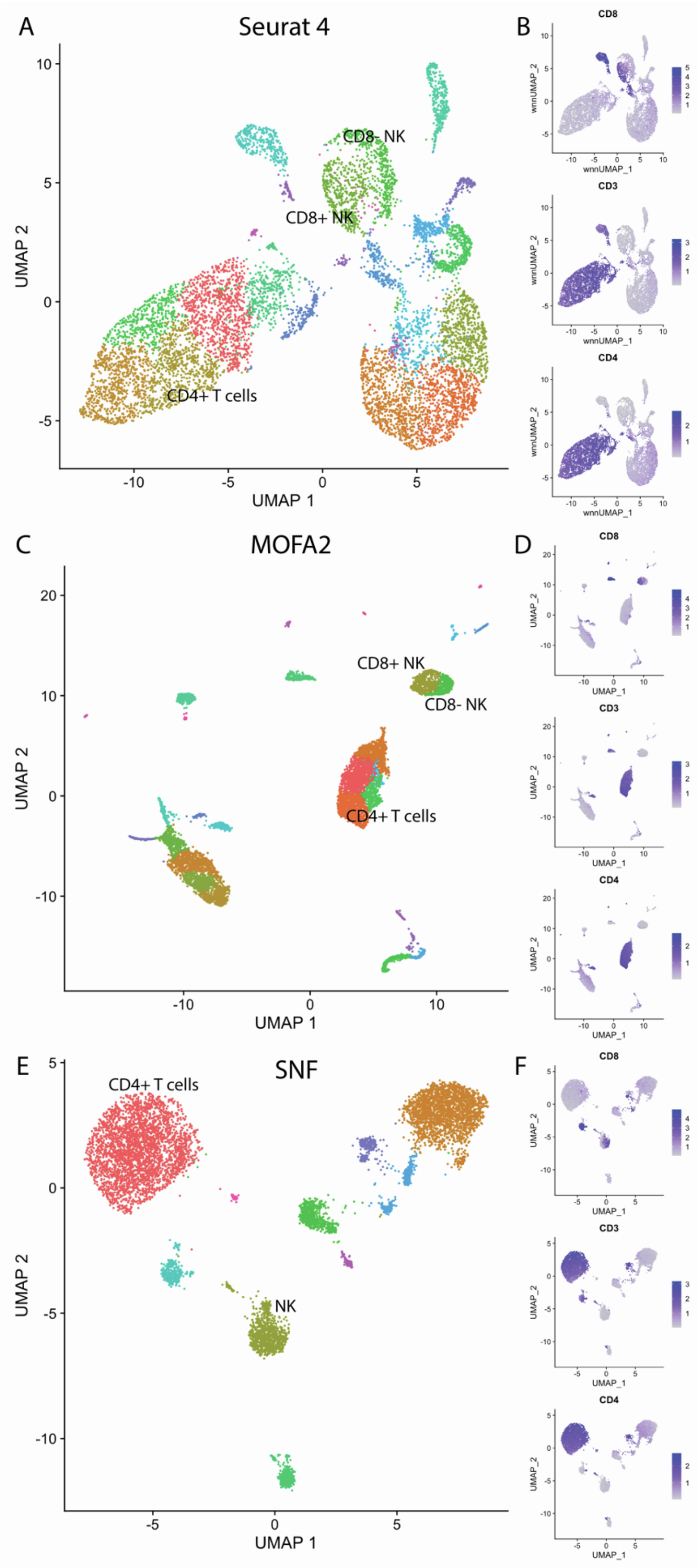
A
Seurat 4
CD8- NK
CD8+ NK
CD4+ T cells
UMAP 2
UMAP 1
B
CD8
CD3
CD4
wnnUMAP_2
wnnUMAP_1
C
MOFA2
CD8+ NK
CD8- NK
CD4+ T cells
UMAP 2
UMAP 1
D
CD8
CD3
CD4
UMAP_2
UMAP_1
E
SNF
CD4+ T cells
NK
UMAP 2
UMAP 1
F
CD8
CD3
CD4
UMAP_2
UMAP_1

Figure S7. Cell clusters generated by three existing multimodal methods, Seurat 4, MOFA2, and SNF, were visualized on their optimized UMAP embedding, related to Figure 6. (**A**) Cell subsets generated by Seurat 4 were visualized on wnnUMAP embedding. (**B**) Expression of CD8, CD3 and CD4 proteins on Seurat 4 wnnUMAP embedding. (**C**) Cell subsets generated by MOFA2 were visualized on UMAP embedding. (**D**) Expression of CD8, CD3 and CD4 proteins on MOFA2 UMAP embedding. (**E**) Cell subsets generated by SNF were visualized on UMAP embedding. (**F**) Expression of CD8, CD3 and CD4 proteins on SNF UMAP embedding.

Table S1. Assessing the quality of NK subsets generated by three different multimodal methods using RNA and ADT purity scores, related to Figure 6.

	Method	CD8+ NK	CD8- NK	Average
RNA score	LinQ-View	0.9552	0.9434	0.9493
	MOFA2(res=1.5)	0.9431	0.9646	0.9539
	Seurat4	0.9408	0.9082	0.9245
ADT score	LinQ-View	0.7822	0.8279	0.8051
	MOFA2(res=1.5)	0.6945	0.8390	0.7668
	Seurat4	0.7787	0.8089	0.7938

Table S2. Assessing the quality of CD4 T cell subsets generated by three different multimodal methods using RNA and ADT purity scores, related to Figure 6.

Method	RNA score	ADT score	Cluster ID
LinQ-View	0.971	0.8965	0
	0.9756	0.9005	2
MOFA2(res=1.5)	0.97	0.9463	0
	0.9679	0.908	1
	0.9649	0.9131	2
	0.9681	0.8847	10
Seurat4	0.9474	0.9273	0
	0.966	0.9467	1
	0.973	0.9584	5
	0.9628	0.9427	6
	0.7267	0.7903	10

Table S3. Top 20 gene markers identified among 5 cell subsets identified by Seurat4 from $CD4^+$ T cells. Gene markers were identified using “FindAllMarkers” function from the entire dataset, related to Figure 6.

Gene	p_val	avg_log2FC	pct.1	pct.2	p_val_adj	Cluster
IL7R	1.69E-194	1.276101	0.867	0.363	3.46E-190	0
LDHB	2.76E-175	1.094745	0.957	0.593	5.67E-171	0
IL32	9.81E-169	1.047846	0.902	0.412	2.01E-164	0
LTB	5.47E-158	1.022945	0.98	0.597	1.12E-153	0

ITM2A	2.15E-150	1.167231	0.786	0.368	4.40E-146	0
CD3E	3.52E-145	1.123032	0.78	0.358	7.21E-141	0
TRAC	8.33E-143	1.108343	0.768	0.339	1.71E-138	0
CD3D	7.51E-136	1.005698	0.855	0.43	1.54E-131	0
NOSIP	3.26E-133	1.045382	0.832	0.455	6.68E-129	0
TRBC2	1.25E-116	0.975419	0.858	0.496	2.55E-112	0
TXNIP	1.02E-103	0.857804	0.926	0.668	2.10E-99	0
SELL	6.87E-101	0.871167	0.853	0.526	1.41E-96	0
SOX4	1.22E-99	0.993059	0.68	0.336	2.51E-95	0
CD27	3.44E-88	0.926274	0.548	0.241	7.05E-84	0
LEF1	1.52E-86	0.924831	0.553	0.248	3.11E-82	0
CD3G	7.20E-86	0.886763	0.603	0.282	1.48E-81	0
PIK3IP1	1.28E-83	0.917302	0.517	0.227	2.62E-79	0
PRKCQ-AS1	1.14E-72	0.905752	0.45	0.194	2.34E-68	0
CAMK4	7.53E-67	0.862052	0.415	0.178	1.54E-62	0
MYC	6.25E-56	0.878191	0.393	0.179	1.28E-51	0
IL7R	2.72E-187	1.309592	0.884	0.368	5.58E-183	1
LDHB	5.83E-161	1.090877	0.964	0.598	1.20E-156	1
LTB	1.07E-154	1.099006	0.986	0.602	2.19E-150	1
ITM2A	1.60E-152	1.254165	0.817	0.37	3.28E-148	1
CD3D	3.28E-148	1.068008	0.898	0.431	6.72E-144	1
TRAC	9.55E-148	1.21783	0.803	0.341	1.96E-143	1
CD3E	3.18E-138	1.04337	0.82	0.359	6.52E-134	1
IL32	2.25E-137	1.024577	0.894	0.419	4.61E-133	1
TRBC2	1.49E-108	0.949362	0.879	0.498	3.06E-104	1
NOSIP	4.11E-108	0.960899	0.828	0.461	8.43E-104	1
LEF1	3.63E-92	0.973727	0.589	0.249	7.44E-88	1
CD27	3.19E-87	0.948812	0.571	0.243	6.53E-83	1
SELL	2.18E-85	0.865475	0.839	0.532	4.47E-81	1
CD3G	1.01E-84	0.909714	0.622	0.284	2.07E-80	1
OCIAD2	1.46E-77	0.917021	0.622	0.313	2.99E-73	1
CAMK4	3.68E-77	0.929688	0.455	0.176	7.55E-73	1
PIK3IP1	6.22E-75	0.869511	0.527	0.229	1.27E-70	1
SOX4	7.63E-71	0.884298	0.651	0.344	1.56E-66	1
CORO1B	7.60E-68	0.91084	0.603	0.321	1.56E-63	1
MYC	3.89E-56	0.870035	0.41	0.18	7.98E-52	1
RPL3	2.76E-104	0.848651	1	0.864	5.65E-100	5
LTB	1.44E-103	1.025716	0.993	0.614	2.96E-99	5
IL7R	1.04E-97	1.192832	0.824	0.389	2.12E-93	5

IL32	4.99E-94	0.977211	0.889	0.435	1.02E-89	5
LDHB	1.99E-93	1.000393	0.935	0.612	4.08E-89	5
TRAC	1.52E-89	1.14572	0.765	0.358	3.12E-85	5
CD3E	8.33E-89	1.117801	0.778	0.377	1.71E-84	5
TRBC2	3.12E-85	1.044435	0.88	0.511	6.39E-81	5
CD3D	7.14E-84	0.969653	0.857	0.449	1.46E-79	5
ITM2A	2.41E-77	1.009084	0.769	0.388	4.95E-73	5
SOX4	4.18E-63	1.01867	0.676	0.352	8.56E-59	5
TXNIP	7.69E-61	0.832261	0.909	0.681	1.58E-56	5
CD3G	7.62E-60	0.93437	0.613	0.295	1.56E-55	5
NOSIP	3.10E-59	0.901435	0.776	0.477	6.36E-55	5
OCIAD2	6.58E-53	0.902851	0.619	0.323	1.35E-48	5
CD27	5.55E-51	0.892827	0.541	0.256	1.14E-46	5
LEF1	5.66E-50	0.917793	0.543	0.263	1.16E-45	5
PCED1B-AS1	5.79E-45	0.846703	0.646	0.374	1.19E-40	5
PRKCQ-AS1	3.44E-39	0.86229	0.435	0.207	7.05E-35	5
RHOH	2.42E-36	0.897578	0.48	0.256	4.97E-32	5
IL7R	2.01E-93	1.184367	0.833	0.391	4.12E-89	6
LTB	1.15E-86	1.062477	0.967	0.618	2.37E-82	6
LDHB	1.04E-82	0.990395	0.935	0.614	2.13E-78	6
CD3E	1.85E-76	0.97756	0.787	0.379	3.79E-72	6
IL32	2.43E-76	0.989485	0.862	0.439	4.99E-72	6
TRAC	2.96E-76	1.101981	0.758	0.361	6.08E-72	6
CD3D	1.95E-74	0.979214	0.846	0.452	4.00E-70	6
CD3G	1.32E-65	0.984378	0.644	0.295	2.71E-61	6
TMEM66	4.00E-61	0.828388	0.919	0.723	8.20E-57	6
NOSIP	2.90E-60	0.917321	0.799	0.477	5.94E-56	6
ITM2A	8.02E-58	0.897442	0.744	0.392	1.65E-53	6
TRBC2	1.64E-57	0.860316	0.843	0.515	3.36E-53	6
LEF1	8.91E-57	0.985366	0.577	0.262	1.83E-52	6
TXNIP	4.93E-53	0.835354	0.892	0.684	1.01E-48	6
CD27	2.18E-50	0.919881	0.551	0.257	4.46E-46	6
SELL	6.24E-47	0.810074	0.799	0.547	1.28E-42	6
JUN	4.14E-42	0.946035	0.699	0.427	8.49E-38	6
TRAT1	3.11E-37	0.8267	0.335	0.132	6.38E-33	6
PIK3IP1	1.44E-35	0.868924	0.478	0.244	2.94E-31	6
CORO1B	1.87E-33	0.836868	0.561	0.335	3.84E-29	6
LTB	3.01E-65	1.009752	0.96	0.623	6.18E-61	10
CD3E	5.78E-64	0.978854	0.815	0.384	1.18E-59	10

IL7R	5.32E-63	0.991946	0.828	0.398	1.09E-58	10
LDHB	8.78E-63	0.873163	0.96	0.617	1.80E-58	10
IL32	4.29E-59	0.926231	0.876	0.445	8.79E-55	10
ITM2A	5.23E-55	0.946844	0.791	0.395	1.07E-50	10
TRAC	7.64E-49	0.824591	0.757	0.367	1.57E-44	10
TRBC2	2.45E-45	0.81613	0.873	0.519	5.02E-41	10
CD3D	3.32E-45	0.749844	0.852	0.458	6.81E-41	10
LEF1	5.96E-43	0.871769	0.59	0.266	1.22E-38	10
RP4-594I10.3	5.83E-39	0.912025	0.426	0.167	1.20E-34	10
SELL	2.98E-37	0.776717	0.82	0.549	6.11E-33	10
NOSIP	1.92E-36	0.770026	0.78	0.483	3.94E-32	10
SOX4	1.14E-34	0.830781	0.667	0.359	2.34E-30	10
PRKCQ-AS1	3.13E-32	0.819758	0.474	0.21	6.41E-28	10
CD27	3.69E-32	0.799766	0.54	0.262	7.57E-28	10
OXNAD1	2.23E-30	0.777623	0.397	0.165	4.57E-26	10
CAMK4	4.87E-30	0.767102	0.434	0.192	9.99E-26	10
PCED1B-AS1	7.36E-29	0.760012	0.661	0.379	1.51E-24	10
MAL	5.89E-26	0.748734	0.307	0.12	1.21E-21	10

Table S4. Gene markers identified among 5 cell subsets identified by Seurat4 from $CD4^+$ T cells. Gene markers were identified using "FindAllMarkers" functions from a subset of $CD4^+$ T cells that only contains the 5 clusters (Cluster 0, 1, 5, 6, and 10), related to Figure 6.

Gene	P value	avg_log2FC	pct.1	pct.2	P_val_adj	Cluster
HPGD	1.02E-06	0.3006893	0.206	0.132	2.09E-02	1
KLRB1	3.51E-03	-0.6330162	0.061	0.101	1.00E+00	5
HLA-DRA	1.39E-03	-0.5163531	0.1	0.155	1.00E+00	5
DUSP1	2.77E-04	-0.4573587	0.224	0.306	1.00E+00	5
FOS	3.83E-06	-0.4569581	0.331	0.443	7.84E-02	5
HPGD	6.74E-05	-0.3458329	0.094	0.163	1.00E+00	5
MOUSE-S100a4	2.31E-03	-0.3361525	0.248	0.32	1.00E+00	5
MOUSE-Anxa1	9.39E-05	-0.3332097	0.072	0.135	1.00E+00	5
ANXA1	6.62E-04	-0.287765	0.456	0.537	1.00E+00	5
CLIC1	9.74E-05	-0.2808579	0.15	0.231	1.00E+00	5
TMEM173	1.09E-04	-0.2604576	0.067	0.127	1.00E+00	5
MOUSE-Prdx1	9.43E-03	-0.2554909	0.069	0.106	1.00E+00	5
DEF6	1.46E-04	-0.2546802	0.12	0.197	1.00E+00	5
TCF25	1.74E-04	0.2733553	0.374	0.309	1.00E+00	5
CTD-3184A7.4	3.58E-03	0.2925256	0.194	0.152	1.00E+00	5
ZFP36L1	3.94E-05	-0.3063632	0.114	0.194	8.08E-01	6

TERF2IP	2.01E-04	-0.2876009	0.244	0.328	1.00E+00	6
YBEY	6.02E-04	-0.2689975	0.061	0.113	1.00E+00	6
ARID4A	5.90E-03	0.2536122	0.114	0.078	1.00E+00	6
GIMAP4	1.75E-03	0.2621582	0.358	0.299	1.00E+00	6
KIAA0020	1.60E-03	0.2626491	0.112	0.072	1.00E+00	6
HNRNPH3	1.94E-03	0.2679293	0.238	0.184	1.00E+00	6
WHSC1L1	8.85E-03	0.2696315	0.319	0.279	1.00E+00	6
YTHDC1	1.95E-03	0.2699329	0.189	0.137	1.00E+00	6
NELL2	1.71E-03	0.274412	0.185	0.135	1.00E+00	6
IFITM1	2.65E-04	0.2938378	0.64	0.606	1.00E+00	6
SRSF7	7.14E-04	0.2945516	0.343	0.278	1.00E+00	6
S100A10	2.10E-03	0.3119543	0.459	0.411	1.00E+00	6
PSMD5-AS1	1.13E-03	-0.359732	0.257	0.321	1.00E+00	10
MYC	7.50E-03	-0.3468814	0.339	0.384	1.00E+00	10
NFKBIZ	1.48E-03	-0.2993363	0.407	0.462	1.00E+00	10
CD3G	6.47E-04	-0.2734616	0.571	0.618	1.00E+00	10
SMC4	6.18E-03	-0.2664461	0.228	0.285	1.00E+00	10
SNRPF	4.39E-03	-0.253839	0.291	0.346	1.00E+00	10
HIF1A	3.15E-03	0.2506162	0.14	0.09	1.00E+00	10
HNRNPH1	3.70E-03	0.251075	0.312	0.238	1.00E+00	10
MOB1A	3.48E-04	0.2554952	0.209	0.136	1.00E+00	10
HERPUD1	1.27E-03	0.2600055	0.206	0.143	1.00E+00	10
GRN	9.70E-06	0.2653345	0.138	0.07	1.99E-01	10
RABGGTB	1.38E-04	0.2681924	0.127	0.071	1.00E+00	10
CYBA	1.34E-04	0.2691447	0.757	0.678	1.00E+00	10
NFKBIA	6.02E-03	0.2710587	0.513	0.439	1.00E+00	10
BTN3A1	5.63E-05	0.2739037	0.108	0.055	1.00E+00	10
RPIA	8.00E-04	0.2820089	0.267	0.192	1.00E+00	10
S100A11	3.01E-03	0.2890378	0.164	0.109	1.00E+00	10
CHCHD5	7.66E-05	0.2915036	0.209	0.131	1.00E+00	10
ANXA2	7.32E-04	0.2916279	0.108	0.062	1.00E+00	10
SRGN	2.76E-03	0.3200517	0.479	0.386	1.00E+00	10
MOUSE-Ctsl	7.15E-04	0.3236493	0.132	0.08	1.00E+00	10
CLIC1	1.75E-04	0.3239855	0.294	0.206	1.00E+00	10
MOUSE-Atp5b	7.44E-03	0.3581136	0.159	0.11	1.00E+00	10
MOUSE-Esd	6.64E-04	0.3631582	0.111	0.064	1.00E+00	10
MOUSE-Ranbp1	5.33E-03	0.3678064	0.132	0.087	1.00E+00	10
AP1S2	3.63E-03	0.3708234	0.254	0.193	1.00E+00	10
MOUSE-Tubb5	8.96E-04	0.3902903	0.193	0.128	1.00E+00	10

MOUSE-Ppp1ca	4.76E-05	0.3939693	0.14	0.077	9.76E-01	10
DUSP1	2.93E-04	0.3943928	0.36	0.282	1.00E+00	10
MOUSE-Cdk4	3.59E-03	0.3959323	0.127	0.08	1.00E+00	10
MOUSE-Bsg	1.25E-03	0.402012	0.209	0.142	1.00E+00	10
MOUSE-S100a10	5.08E-03	0.4590124	0.183	0.128	1.00E+00	10
MOUSE-Slc25a4	9.46E-04	0.4598929	0.161	0.103	1.00E+00	10
CD79B	2.23E-03	0.4696106	0.103	0.063	1.00E+00	10
MOUSE-Hsp90ab1	9.83E-04	0.4730225	0.177	0.117	1.00E+00	10
MOUSE-Cstb	1.34E-03	0.485548	0.138	0.086	1.00E+00	10
CTSS	1.83E-04	0.4864184	0.283	0.201	1.00E+00	10
MOUSE-Pkm	3.98E-04	0.4971615	0.124	0.072	1.00E+00	10
MOUSE-Eef1b2	1.11E-03	0.5054604	0.103	0.059	1.00E+00	10
HLA-DQB1	2.09E-15	0.5610536	0.106	0.026	4.29E-11	10
MOUSE-S100a11	1.50E-04	0.5639075	0.103	0.054	1.00E+00	10
MOUSE-Tmsb4x	6.76E-03	0.5825296	0.45	0.367	1.00E+00	10
MOUSE-Rps19	5.29E-05	0.5836074	0.122	0.064	1.00E+00	10
CD79A	3.72E-09	0.597609	0.101	0.036	7.64E-05	10
FCER1G	4.84E-04	0.5978362	0.183	0.119	1.00E+00	10
CST3	3.98E-03	0.6068051	0.201	0.143	1.00E+00	10
MOUSE-Tmsb10	8.72E-03	0.6252044	0.111	0.072	1.00E+00	10
MOUSE-Lgals1	4.26E-03	0.6288699	0.788	0.708	1.00E+00	10
MOUSE-Rplp2	1.32E-04	0.6352816	0.101	0.052	1.00E+00	10
MOUSE-S100a4	8.35E-03	0.6422301	0.368	0.299	1.00E+00	10
MOUSE-Anxa1	2.00E-06	0.6595185	0.201	0.113	4.10E-02	10
MOUSE-Cd63	1.14E-04	0.6966574	0.183	0.112	1.00E+00	10
MOUSE-Rpl26	4.84E-04	0.7055673	0.119	0.068	1.00E+00	10
HLA-DPA1	1.86E-07	0.7218892	0.108	0.046	3.81E-03	10
LGALS1	2.57E-03	0.7264715	0.172	0.12	1.00E+00	10
MOUSE-Rpl23	4.07E-03	0.7302428	0.148	0.098	1.00E+00	10
MOUSE-Rplp1	5.45E-03	0.7746256	0.138	0.091	1.00E+00	10
TYROBP	1.14E-05	0.7779309	0.23	0.141	2.33E-01	10
MOUSE-Rps15	2.97E-05	0.8176073	0.172	0.1	6.09E-01	10
MOUSE-Gm10260	5.06E-05	0.8242067	0.108	0.054	1.00E+00	10
HLA-DPB1	4.80E-15	0.8777517	0.108	0.028	9.84E-11	10
MOUSE-Rpl13a	2.25E-04	0.8910114	0.286	0.196	1.00E+00	10
HLA-DRB1	1.74E-11	0.935431	0.151	0.059	3.56E-07	10
PPBP	6.98E-07	0.9355044	0.108	0.047	1.43E-02	10
MOUSE-Rps11	3.99E-05	0.9652659	0.169	0.099	8.18E-01	10
MOUSE-Pfn1	7.98E-05	1.0513148	0.201	0.126	1.00E+00	10

CD74	1.60E-04	1.1090925	0.505	0.427	1.00E+00	10
LYZ	8.16E-03	1.2885235	0.325	0.266	1.00E+00	10
HLA-DRA	7.86E-09	1.3855268	0.238	0.132	1.61E-04	10

Table S5. Runtime (seconds) of all processing steps of LinQ-View on multiple single cell datasets, related to Figure 6.

**Dataset**	**Dataset name**	**Dataset1**	**Dataset2**	**Dataset3**	**Dataset4**	**Dataset5**	**Dataset6***
	**Number of cells**	8067	713	7865	8412	5247	19481
**Processing steps**	**Create object**	1.77	0.10	0.57	0.46	0.28	2.45
	**Normalization**	0.29	0.11	0.36	0.31	0.37	9.23
	**Identify HVGs**	1.80	1.06	2.30	2.30	1.97	
	**Data scaling**	0.47	0.26	0.58	0.44	0.38	17.70
	**PCA**	11.22	0.99	13.27	8.61	6.75	39.50
**Joint analysis**	**Joint distance calculation**	26.70	0.54	66.73	72.61	43.12	140.91
	**t-SNE**	31.57	1.32	34.22	38.63	18.33	145.90
	**Clustering**	6.04	0.17	6.47	7.29	2.60	42.56

*This is an integrated dataset from 10 individual datasets. Batch effects among all 10 datasets were removed by Seurat 3, runtime of this step is: 10.06 mins.

Table S6. Runtime (minutes) of kernel step of LinQ-View on massive single cell datasets with different cell numbers (number of ADT feature = 20), related to Figure 6.

**Cell Number**	48170	39264	30076	20799	11424
**Joint distance calculation**	39.39	22.54	12.41	5.81	1.69

Table S7. Parameters used in generating simulated dataset 1. We use absolute values in case of negative values, related to STAR Methods.

	Cluster 1		Cluster 2		Cluster 3		Cluster 4		Cluster 5		Cluster 6	
	Mean	SD	Mean	SD	Mean	SD	Mean	SD	Mean	SD	Mean	SD
Feaure1	0.5	0.5	0	0.5	0.5	0.5	0	0.5	0.5	0.5	0	0.5
Feaure2	3	0.5	3	0.5	3	0.5	3	0.5	3	0.5	3	0.5
Feaure3	1	0.5	0	0.5	2	1.5	0.5	0.5	2	0.5	2	0.5
Feaure4	0.5	0.5	2	1	2	0.5	0	0.5	1.5	0.5	3	0.5
Feaure5	2.5	0.5	0	0.5	3	0.5	3	1	0	0.5	3	1
Feaure6	3	0.5	0	0.5	0.5	0.5	3	1	2.5	1	0.5	0.5
Feaure7	2.5	0.5	2	0.5	0	0.5	3	0.5	2.5	1	2.5	1
Feaure8	0	0.5	2	0.5	3	0.5	3	0.5	2.5	1	3	1
Feaure9	0	0.5	2	0.5	0.5	0.5	3	0.5	0	0.5	3	1
